# Identification of Candidate Genes and Physiological Pathways Involved in Gonad Deformation in Whitefish (*Coregonus* spp.) from Lake Thun, Switzerland

**DOI:** 10.3390/ijerph8072706

**Published:** 2011-06-30

**Authors:** David Bittner, Andrew R. Cossins, Helmut Segner, Laurent Excoffier, Carlo R. Largiadèr

**Affiliations:** 1 Computational and Molecular Populations Genetics Lab, University of Bern, Baltzerstrasse 6, 3012 Bern, Switzerland; E-Mails: David.Bittner@eawag.ch (D.B.); laurent.excoffier@iee.unibe.ch (L.E.); 2 Liverpool Microarray Facility, School of Biological Sciences, University of Liverpool, L69 7ZB Liverpool, UK; E-Mail: cossins@liverpool.ac.uk; 3 Centre for Fish and Wildlife Health, University of Bern, Laenggass-Strasse 122, PO-Box 8466, 3001 Bern, Switzerland; E-Mail: helmut.segner@itpa.unibe.ch; 4 Institute of Clinical Chemistry, University Hospital, University of Bern, Inselspital, 3010 Bern, Switzerland

**Keywords:** oligonucleotide microarrays, gene expression, reproductive organ, gene expression profiling, autoimmunity, complement

## Abstract

In 2000, fishermen reported the appearance of deformed reproductive organs in whitefish (*Coregonus* spp.) from Lake Thun, Switzerland. Despite intensive investigations, the causes of these abnormalities remain unknown. Using gene expression profiling, we sought to identify candidate genes and physiological processes possibly associated with the observed gonadal deformations, in order to gain insights into potential causes. Using *in situ*-synthesized oligonucleotide arrays, we compared the expression levels at 21,492 unique transcript probes in liver and head kidney tissue of male whitefish with deformed and normally developed gonads, respectively. The fish had been collected on spawning sites of two genetically distinct whitefish forms of Lake Thun. We contrasted the gene expression profiles of 56 individuals, *i.e.*, 14 individuals of each phenotype and of each population. Gene-by-gene analysis revealed weak expression differences between normal and deformed fish, and only one gene, ictacalcin, was found to be up-regulated in head kidney tissue of deformed fish from both whitefish forms, However, this difference could not be confirmed with quantitative real-time *q*PCR. Enrichment analysis on the level of physiological processes revealed (i) the involvement of immune response genes in both tissues, particularly those linked to complement activation in the liver, (ii) proteolysis in the liver and (iii) GTPase activity and Ras protein signal transduction in the head kidney. In comparison with current literature, this gene expression pattern signals a chronic autoimmune disease in the testes. Based on the recent observations that gonad deformations are induced through feeding of zooplankton from Lake Thun we hypothesize that a xenobiotic accumulated in whitefish via the plankton triggering autoimmunity as the likely cause of gonad deformations. We propose several experimental strategies to verify or reject this hypothesis.

## 1. Introduction

In the year 2000, commercial fishermen reported the sudden appearance of deformed reproductive organs in whitefish (*Coregonus* spp.) from Lake Thun, Switzerland. A detailed first survey of the variation of gonad morphology in whitefish of this lake suggested that 35% of the examined fish had aberrant gonad morphology [1]. A more detailed study [2] indicated that, although a certain proportion of the observed variation in gonad morphology can be attributed to natural variation, the quantity and quality of gonadal deformations in Lake Thun whitefish goes clearly beyond that level of natural variability [1]. Furthermore, Bittner *et al.* [2] also found that these deformations occurred predominantly at particular spawning sites of different whitefish forms that coexist in sympatry in Lake Thun.

Many studies were initiated in search of potential causes for the unusual gonad deformations. Most of them focused on chemical contaminants, in particular on endocrine-disrupting compounds since these substances can alter gonad morphology in fish [3–6]. To date, no unequivocal evidence for chemicals as causative agents have been discovered [7–9]. It appears that neither inbreeding effects nor recent hybridization between genetically distinct whitefish forms are involved [10]. Inheritance of gonad deformations [11] and improper breeding of whitefish at the local hatchery [1] have also been excluded. Thus, despite the intensive research having been conducted to date, the causes of gonad deformations in whitefish from Lake Thun remain unknown with the exception of some rearing experiments that point to a role of the lake plankton [7,11].

In recent years, gene expression profiling by means of microarray analyses has become an increasingly popular tool to assess the impact of potential stressors on biological pathways at the level of mRNA transcription, especially in fish, which are increasingly used as models for studies of environmental genomics [12]. Analyses of the transcriptome provide insight into the molecular control of biological and physiological processes and specifically into response to disease, toxins, environmental challenge and other stressors [13,14]. Many of the stressor exposures for which there exists data have been accomplished in laboratories under controlled conditions. So far, only few microarray studies have been conducted with field samples because of the risk of high noise effects due to variation of sex, genotype, age and intrinsic genetic variability in gene expression in natural populations [15–18] that makes it difficult to sort out specific stressor effects. In these studies, usually a gradient approach was used where local fish populations under stress were compared to less impacted sites or to “reference” sites where the stressor was absent. Thus, European flounder (*Platichthys flesus*) from the polluted Tyne were compared to the unpolluted Alde estuaries in the UK [19]. In another study, transcriptomic profiles of wild *Fundulus* from several superfund sites (highly polluted) and nearby reference sites along the Atlantic coast were compared [16,20,21]. However, to our knowledge, arrays have not been used to date to compare fish from the same environment but displaying different manifestations of a phenotypic trait.

Here, we applied gene expression profiling to identify new candidate genes and physiological processes associated with observed gonad deformations in whitefish from Lake Thun, and thus, to obtain insight into new potential causes. By using an *in-situ* synthesized rainbow trout oligonucleotide array [22], we compared expression levels of 21,492 transcripts in head kidney and liver tissue between male whitefish with normal and deformed gonads. Since we aimed at screening for gene expression patterns pointing to particular xenobiotic substances potentially contained in the lake plankton, we chose to study tissues of the main organs with respect to detoxification processes or excretion of toxic compounds. Liver is an important organ for many detoxification processes and the head kidney is a key excretory organ of toxic compounds in fish. Transcription profiles were analyzed with two complementary approaches. First, in order to identify candidate genes, we used an ANOVA-based gene-by-gene approach (*cf.* [23]) to infer expression differences between groups of fish in pairwise comparisons associated with the deformation of gonads. Secondly, using the Gene Ontology (GO) database, we categorized annotated transcript probes into functionally meaningfully groupings according to molecular functions, biological processes or cellular locations in order to identify physiological pathways that are associated with deformed gonads.

## 2. Experimental Section

### 2.1. Sampling

Lake Thun harbours several distinct sympatric whitefish forms [10]. A large-scale epidemiological survey revealed that gonad deformations occurred predominantly in the summer-spawning “Brienzlig” whitefish form and additionally in one population of the winter-spawning “Albock” form [2]. The two forms display significantly different, though partially overlapping distributions in gill rakers counts, which is an important adaptive and highly heritable morphological trait in whitefish [24].

For microarray analyses, we included only male whitefish in our study in order to avoid additional, sex-related variance in gene expression and because males showed significantly higher frequencies of gonad deformations compared to females [2]. We aimed at comparing the two most distinct gonadal phenotypes: (i) fish with near perfectly symmetrical gonads (normal gonads) and (ii) fish showing adhesions/fusions of testis to the peritoneal wall and the lateral trunk musculature (deformed gonads).

Fish were caught with bottom gill nets and their gonad morphology was macroscopically immediately analyzed. For individuals, which fulfilled our criteria of normal and deformed gonads, liver and head kidney samples were extracted, subsequently transferred into RNA stabilization reagent (RNA Later, Ambion) and stored at −30°C prior to RNA extraction. Two hundred and sixty eight (268) male whitefish were sampled from two Brienzlig spawning sites at depths between 100–200 m in September 2005. Fusions (*i.e.*, deformed gonads) were observed in 52 individuals (19.4%). We pooled samples from the two sites, since the two spawning populations were not significantly different in allele frequencies at 11 microsatellite loci [25]. Furthermore, 231 male whitefish were collected from an Albock spawning site at a depth of about 50m in December 2005 as an independent biological replicate. In this sample, 25 fish (10.82%) with fusions were present.

The age of each sampled fish was determined using the annulus-criteria of [26]. Additionally, the first gill arch was removed in order to determine gill raker counts, which were counted from the anterior left gill arch in the laboratory using a binocular. Furthermore, a small liver sample from each fish was stored in absolute ethanol for subsequent genotyping at 11 microsatellite loci as described in [25].

### 2.2. Experimental Design

In order to control for possible technical and biological effects on individual transcription profiles, in a first step we homogenized the samples with respect to genetic population association and in a second step we matched the composition of the samples with regard to age and gill raker numbers as good as possible. In more detail, assignment tests [27,28] were carried out with the program GENECLASS 2.0 [29] in order to identify outlier genotypes. Individuals with unlikely multilocus genotypes, *i.e.*, P < 0.01 in their respective parental populations were excluded. Outlier individuals with respect to age and gill raker counts were also excluded. Means and standard deviations for the above parameters as well as weight and length measurements of sampled fish are given in Table S1.

The use of the two whitefish forms as independent biological replicates was verified in this study by assessing the expected morphological and genetic differentiation between the two populations. In detail, the Albock and the Brienzlig displayed significant differences in gill raker counts (Table S1) and were highly significantly genetically differentiated, (F_ST_ = 0.136; P < 0.00001; 95% confidence interval: 0.064–0.216). In contrast, no genetic and morphological differentiation between normal and deformed fish within both forms was observed (all P > 0.05; Table S1).

In order to estimate the reproducibility of the array experiments, we replicated the analysis of four head kidney and four liver samples on a different array slide using independently extracted and labelled RNA-samples from the same tissue samples. We observed a high mean squared correlation coefficient (R^2^) between slide replicates of 0.97 for liver tissue and of 0.94 for head kidney tissue, respectively. Reproducibility of spot replicates on a slide was similarly assessed by comparing the raw intensity measurements of spot replicates within all arrays: R^2^ was 0.99 for liver (range: 0.983–0.993) and for head kidney (range: 0.989–0.994), respectively, indicating the high quality of the arrays used in this study.

We used the two-colour microarray-based approach and carried out non-competitive hybridization experiments. In total we analyzed transcription profiles of 112 samples (56 liver and 56 head kidney samples). Liver samples were labelled with Cy3 and head kidney samples with Cy5 and were then co-hybridized on the same array. Since we used 4 × 44 k arrays hybridizations were carried out simultaneously for four different whitefish samples incorporating liver and head kidney samples adding to a total of eight array experiments on each slide. One sample per group (*i.e.*, Albock deformed, Albock normal, Brienzlig deformed, and Brienzlig normal) was present on each slide.

To avoid hidden systematic biases we carefully randomized all stages of the microarray experiment (RNA extraction, cDNA labelling and hybridizations) by processing different sets of samples of the four population groups. To avoid further systematic biases hybridizations and washings were simultaneously carried out for two batches of seven slides. The technical replicate slide was hybridized and washed individually.

### 2.3. Microarrays

Since no microarray designed for *Coregonus* species was available to date, we used an *in situ*-synthesized oligonucleotide array chip originally designed against rainbow trout targets [22]. Owing solely to sequence divergence, the number of features that a single-species microarray can detect in targets from another species is expected to decrease with increasing phylogenetic divergence. The use of a microarray generated from one species to measure gene expression in another is termed “heterologous hybridization” [30]. We tested hybridization of *Coregonus* spp. targets to rainbow trout probes in a preliminary experiment to labelled cDNA from two rainbow trout and two whitefish specimens from liver and head kidney tissues respectively on 22 k custom arrays fabricated by Oxford Gene Technologies (OGT, Oxford, UK) [22]. We found a comparable number of expressed genes in both species in liver and head kidney tissue (Table S2). In addition, log2 transformed raw intensity frequency histograms showed also comparable patterns between the two species as well as for both tissues (Figure S1). These results validate the heterologous use of these probes for *Coregonus* species.

For our array experiment we used 15 Agilent 4 × 44 k custom arrays with four identical arrays containing two copies of the 21,492 unique 60-mer oligonucleotide gene probes (*i.e.*, spot replicate) as well as negative control probes. The performance of these arrays is excellent in that the dynamic range, spot quality and spot-to-spot consistency exceeds that of typical cDNA arrays by some margin [22].

### 2.4. cRNA Synthesis, Hybridizations, Imaging and Feature Extraction

Total RNA was extracted automatically using the RNeasy Plus kit on the QiaCube robot (both Qiagen) using 10mg of frozen tissue stabilized in RNA Later (Ambion). Quality and quantity assessment of the extracted RNA were performed using a NanoDrop ND-1000 UV-VIS Spectrophotometer v.3.2.1 and Bioanalyzer (Agilent). All RNA samples had RIN values of > 8 (average mean RIN for liver = 9.36; average mean RIN for head kidney = 9.34) with the exception of one liver sample (RIN = 7).

Sample preparation, labelling, hybridization, washing, scanning and feature extraction was performed using Agilent’s two-colour microarray-based gene expression analysis v.5.5 protocol. We used Agilent’s Low RNA Input Linear Amplification Kit Plus to generate fluorescent cRNA. Quality (*i.e.*, integrity) of the labelled cRNA was checked with Agilent’s Bioanalyzer using the RNA 6000 Nano LabChip kit. To assess the quantity of cRNA we used the NanoDrop ND-1000 UV-VIS Spectrometer. Hybridizations were carried out overnight for 17 h at 65 °C.

Slides were washed and then scanned immediately on an Agilent DNA microarray scanner (5 μm resolution) to minimize the impact of environmental oxidants on signal intensities. Finally, gene expression levels were quantified using Agilent’s Feature Extraction software v.9.5.3. Median local background signal intensities were subtracted from raw median signal intensities of each spot. Non-uniform spots, non-uniform local backgrounds as well as saturated spots were flagged by Feature Extraction software. For liver, out of the 60 array experiments a mean of 0.09% (range: 0.016–1.047%) of the relevant 42,984 spots per array were flagged. For head kidney slightly more spots, *i.e.*, 0.372% (0.184–1.04%) per array were flagged. For subsequent analysis, the technical replicate slide was excluded.

### 2.5. Normalization and Data Filtering

Supported by raw signal intensity distribution of spot replicates, raw data were log2 transformed. In order to make data comparable across slides, we applied a robust non-linear method for normalization using array signal distribution analysis and cubic splines [31]. We then applied a spot replicate filter, where the absolute distance between pairs of spot replicates was accounted for. In cases where features with absolute distance > 4 times the standard deviation of the population mean between each other were found, the outlying feature was discarded. For liver, a mean of 1.59% features (range 0.61–4.27%) per array and for head kidney, a mean of 0.51% features (range 0.16–1.88%) per array were excluded by spot replicate filtering. Then data were normalized again. As background level of unspecific hybridization, we considered the mean of negative control probes plus two standard deviations in each tissue. Only probes with mean signal intensities above this level in at least one of the groups were considered for further statistical treatment. Additionally, we applied a missing data filter, *i.e.*, features with less than 50% of data in at least one of the groups were excluded from further analyses.

### 2.6. Gene-by-Gene Analysis

In order to identify genes involved in gonad deformation, pairwise hierarchical analysis of variance (ANOVA) between following combinations of the four groups were performed: (i) Albock normal *vs.* Albock deformed; (ii) Brienzlig normal *vs.* Brienzlig deformed; (iii) all normal fish (Albock and Brienzlig pooled) *vs.* all deformed fish and additionally a “form” comparison (iv) all Albock (normal and deformed pooled) *vs.* all Brienzlig. ANOVAs were calculated separately for each gene in both tissues separately. The following nested ANOVA model was considered:

(1) $$y_{ijk}=\mu +a_{i}+b_{ij,}+c_{ijk}$$

where *y_ijk_* is the log2 gene expression intensity for a given gene probe for the *k*-th spot replicate in the *j*-th individual in the *i*-th experimental group. The effects for gonadal phenotype (a), individual (b) and spot replicate (c) are assumed to be independent and random. Therefore total variance can be calculated:

(2) $${\sigma_{T}}^{2}={\sigma_{a}}^{2}+{\sigma_{b}}^{2}+{\sigma_{c}}^{2}$$

where *σ_a_^2^*, *σ_b_^2^*and *σ_c_^2^* are the associated variance components. Note that under this setting, *σ_a_^2^* is also equal to the covariance in gene expression levels between two individuals from the same group [32], and *σ_b_^2^* is the covariance in gene expression levels between two spot replicates from the same individual. Therefore, in a way similar to what is done with genetic data, we can define the following intra-class correlation coefficient:

(3) $$M_{ST}={\sigma_{a}}^{2}/{\sigma_{T}}^{2}$$

*M_ST_* is the correlation in gene expression between two individuals from the same group relative to two individuals from two different groups. *M_ST_* can also be considered as the proportion of total variance in gene expression differences that can be explained due to differences between the groups.

Significance levels of gene-by-gene analysis were estimated using 10,000 permutation tests, as described elsewhere for genetic data [33]. If none of the permutations revealed a mean difference larger than the observed, additional permutations were carried out until at least two permutations were present above criteria (this only accounted for very few probes and was only carried out for comparisons of normal *vs.* deformed fish). Advantage of our ANOVA based approach was to neglect assumptions regarding normal distributions of normalized gene expression levels as well as equal variances between groups and among individuals (see [23]). In order to adjust for multiple hypotheses testing, false discovery rate (FDR) was applied [34].

### 2.7. Gene Ontology Analysis

Information about genes is increasingly being captured and organized in the Gene Ontology (GO) nomenclature [35]. Enrichment analysis identifies GO categories that are enriched among the genes that are differently expressed between groups. Gene Ontology terms for the 21,492 gene probes were obtained by downloading corresponding GO terms from the Gene Ontology Annotation (GOA) database using the quickGO tool [36]. Out of the 21,492 unique gene probes on our array, which in some cases were annotated to identical protein identifiers (ID), 8,269 IDs possessed corresponding GO annotations (all of which were IDs annotated to SwissProt database).

Since gene-by-gene analysis did not yield large sets of significantly different expressed genes between normal and deformed fish (see results) all genes were included in the GO analyses by ranking them according to their *P*-values. Furthermore, in order to focus on the identification of GO categories primarily associated with gonad deformations, we only used co-regulated gene probes (*i.e.*, same direction of regulation of gene expression) of the two within whitefish form comparisons of normal *vs.* deformed fish. The pooled analysis, *i.e.*, the comparison between all normal fish *vs.* all deformed fish was discarded (see discussion). For GO analysis, in order to reduce redundancy due to groups of gene probes having the same protein IDs, we included only the gene probe of each such group into the GO analyses showing lowest combined *P*-values (as calculated by the procedure of Fisher’s Combination [37]).

We used the Wilcoxon rank test procedure implemented in the program FUNC [38] to identify enrichment of particular gene annotations. FUNC uses permutations in order to model the distribution under the null hypothesis that gene associated variables (*i.e.*, *P*-values) are independent of gene annotation for significance testing. We selected the three root ontologies (*i.e.*, GO taxonomies: “biological process”, “molecular function”, “cellular component”) on which the tests were performed and restricted tested categories to those containing a minimum of 20 genes (default value). Results of GO analysis were further narrowed down with a “refinement analysis”, also implemented in FUNC, using only GO categories with arbitrary threshold of *P* < 0.01. This procedure excluded GO categories that were significant solely because they contained significant descendant categories. Consequently, the list of categories was limited to the most specific significant GO terms. Only categories with number of genes < 200 were considered from the resulting list of significant GO groups due to otherwise confusing and unspecific large gene lists.

### 2.8. Hierarchical Clustering

Finally, in order to visualize expression patterns of particular genes involved in gonad deformations, we performed a two-way clustering analysis [39], simultaneously classifying genes and groups based on mean gene expression differences. In detail, we included all genes that were in common among certain GO categories between the two whitefish forms for each tissue respectively (see results). For each gene probe, group means were divided by the global mean across all four groups in order to account for heterogeneity in magnitudes of expression between the different genes. As clustering algorithm, we applied unweighted pair group method with the arithmetic mean (UPGMA) [40] and used centered Pearson correlation coefficients as distance measure. Hierarchical clustering analysis was carried out using the MeV v.4.3.01 software [41].

### 2.9. Real-Time qPCR Analysis

A new real-time PCR assay was established for the Ictacalcin gene. Prior to the design of the assay, sequence analyses were preformed for this gene to obtain detailed sequence information on the regions to be amplified in the real-time PCR reaction. The regions of interest were amplified on a GeneAmp® PCR System 9700 (Applied Biosystems) in a reaction volume of 25 μL, using cDNA from at least one whitefish individual analysed as a template and the following primers sequences: 5′-CACAGGTCCAGCAGGGTATG-3′ and 5′-AAGAACTCATTGCACATCATGG-3′. PCR conditions using Taq polymerase (Qiagen) were the following: Initial activation and denaturation at 95 °C for 10 min, followed by five cycles with denaturation at 95 °C for 1 min, annealing at 55 °C for 30 s, elongation at 72 °C for 1 min, and 25 cycles with denaturation at 95 °C for 30 s, annealing at 55 °C for 30 s and elongation at 72 °C for 1 min with a subsequent final step of 10 min at 72 °C. Terminator Ready Reaction Mix “Big Dye” version 3.1 (Applied Biosystems) and both primers for each gene to sequence both strands in a 10 μL reaction volume. The following thermal profile was used for cycle sequencing: Initial denaturation for 50 s at 90 °C, followed by 25 cycles with denaturation at 90 °C for 10 s, annealing at 50 °C for 10 s and elongation at 60 °C for 4 min. Subsequently, products were cleaned using the DyeEx 96 spin kit (Qiagen) and separated on an ABI Prism 3130XL Genetic Analyser (Applied Biosystems).

For RT-PCR, a custom TaqMan® gene expression assay (Applied Biosystems) was used. The primers for Icatcalcin have been designed as 5′-CCTGAGCAAGGGAGAACTCA-3′ and 5′-CCTTTGCCTGGTCAGTGTTT-3′ and as probe 5′-TCTGCTCAACGCTGAGCTTGGAGAGA-3′. Transcript levels were normalized against 18S, for which primers and TaqMan probes are commercially available (Applied Biosystems). RT-PCR was performed with the 7500 fast real-time PCR system (Applied Biosystems) using 96 wells plates (MicroAmp, fast optical 96-well reaction plate and–MicroAmp optical adhesive film, Applied Biosystems) according to the Custom TaqMan® gene expression assays protocol in duplicate. For the estimation of 18S levels, cDNA was diluted 1:1,000 in RNAse-free water before addition to the 25-μL reaction. As internal standard for every PCR plate, we used a four replicates of of cDNA from a reference individual to determine interassay variability. For each assay, the amplification efficiency was determined based the slopes of standard curves. Relative quantification of the gene expression data was done using the method proposed by Paffl [42].

## 3. Results and Discussion

### 3.1. Gene-by-Gene Analysis

In liver, 7,013 and in head kidney 17,834 gene probes showed signal intensities above background, all of which were considered for statistical analyses. In general, we observed only weak differences in gene expression between normal and deformed fish. The subtle differences were more pronounced in head kidney compared to liver. However, no single gene showed significantly different gene expression between normal and deformed fish after *P*-value adjustments for multiple hypotheses testing with FDR ≤ 5% in both tissues and whitefish forms respectively. In contrast, when comparing transcription profiles between the two whitefish forms 2,764 genes in the liver (39.4%) and 12’820 genes in the head kidney (71.9%) showed significant expression differences (FDR ≤ 5%; Table 1).

At the nominal *P*-value of 0.01 in the liver 78 gene probes with FDR of 88% (*i.e.*, nine gene probes assigning as true positives) were found, but FDR of 100% was observed for the Brienzlig comparison (Table 1a). In the head kidney, in the Albock, 264 gene probes and in the Brienzlig 528 gene probes were observed at the 1% nominal level with FDRs of 68% and 31% respectively (*i.e.*, 84 gene probes assigning as true positives for the Albock and even 364 for the Brienzlig; Table 1) indicating weak differences in gene expression between normal and deformed fish at the level of individual genes.

**Figure 1:**
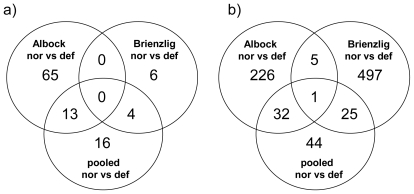

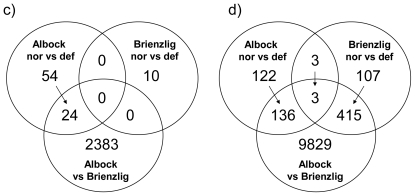
Venn diagrams showing the number of genes differently expressed between whitefish with normal (nor) and deformed (def) gonads. (**a**) and (**b**) show within-form comparisons contrasted to the pooled normal and deformed fish comparison and (**c**) and (**d**) the within-form comparisons contrasted to the comparison between all Albock and all Brienzlig individuals. Only genes with *P*-value < 0.01 for liver (a & c) and head kidney (b & d) are considered.

**Figure 2:**
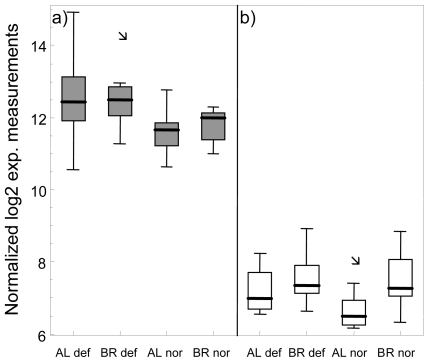
Box plots of log2 normalized gene expression measurements of Ictacalcin in head kidney (**a**) and liver tissue (**b**) of normal (nor) and deformed (def) Albock (AL) and Brienzlig (BR) whitefish. Shown are median (vertical line), 25% and 75% quartiles (box), and minimum and maximum value (error bars). Outliers depicted as small squares represent data points that are more than 1.5 times the interquartile range away from the box.

**Figure 3:**
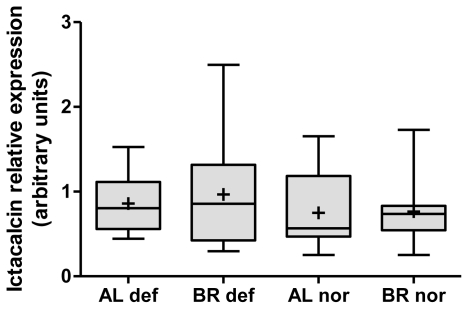
Box plots of relative RT *q*PCR gene expression measurements of Ictacalcin in head kidney tissue of normal (nor) and deformed (def) Albock (AL) and Brienzlig (BR) whitefish. Shown are median (vertical line), mean (cross), 25% and 75% quartiles (box), and minimum and maximum values (error bars).

Investigating congruencies among the two whitefish forms, in the liver, no gene was found at the nominal *P*-value of 0.01 in the three comparisons of normal *vs.* deformed fish and also not in the intersection between the two within-form comparisons (Figure 1a). In contrast, in head kidney, applying the same criteria, one gene, encoding for Icatcalcin, was found in all three comparisons. Another five genes were observed in the intersection of the two within-form comparisons (Figure 1b). Out of these six genes only Ictacalcin was co-regulated in both forms with up-regulation in deformed fish as compared to normal fish. Ictacalcin was also expressed at much higher levels in the head kidney as compared to the liver (Figure 2). In support of this finding two independent gene probes of Ictacalcin accounted for the top and third position by means of *P*-value rankings in the pooled comparison of all normal *vs.* all deformed fish (Table S3). No significant difference in Ictacalcin expression between deformed and normal fish was observed when reanalysing the head kidney samples using RT *q*PCR (Figure 3), although mean and median gene expression levels of deformed fish were higher in deformed fish in both whitefish forms.

**Figure 4:**
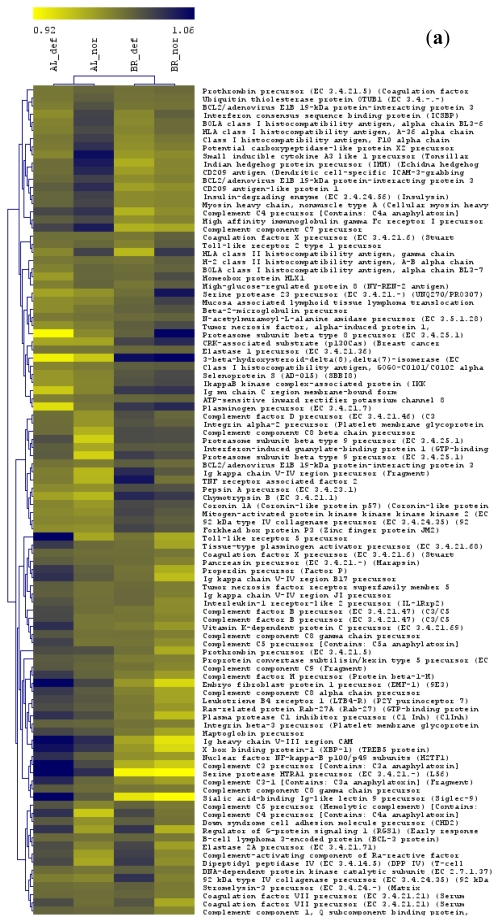

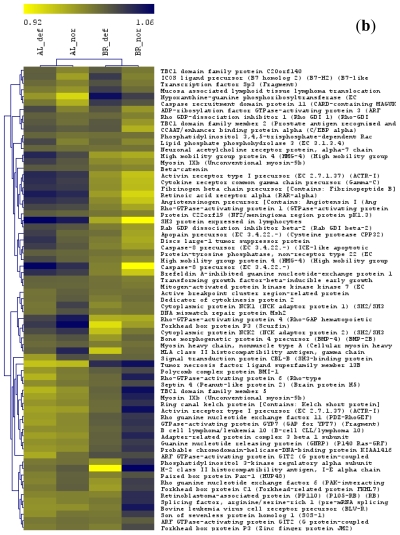
Transcript abundance patterns for 102 genes in the liver (**a**) and 71 genes in the head kidney (**b**) found in common across the significant GO categories in comparisons of deformed and normal whitefish of the Albock and the Brienzlig form, respectively. As clustering algorithm, average linkage (UPGMA) was applied. Centered Pearson correlation coefficients were used as distance measures. The dendrogram on the left groups genes with similar expression and the dendrogram on the top separates populations. The colour bar indicates arbitrary expression units. For each gene probe, the mean gene expression levels of each group divided by the global mean across all four groups are shown. Yellow colour (light) designates low expression and purple colour (dark) represents high expression levels respectively.

When the two within-form comparisons of normal *vs.* deformed fish were contrasted against the “form comparison”, we observed a general increase in the numbers of genes in the intersections of the Venn diagrams (as indicated by arrows; Figure 1c and 1d). In other words, many of the genes that were present in the comparisons of normal *vs.* deformed fish, were also present in the comparison between the two whitefish forms. Therefore, when considering the pooled comparison, a substantial number of genes was affected by the strong differences in gene expression between the two whitefish forms. This finding can be described as a “form effect”. The “form effect” is well illustrated by hierarchical clustering analysis (see below). A large number of genes were present, which showed similar expression differences between normal and deformed fish, but which were expressed on different levels with regard to the two whitefish forms (Figure 4a and 4b).

In order to test, whether the strong differences between the two whitefish forms are caused by a technical bias, we carried out the analysis by normalizing the raw data for each form separately. We found that population normalized intensity measures did not change noticeably as compared to a normalization procedure carried out across both whitefish forms (R^2^ > 0.9999 in both groups). Therefore, based on these results and the above described “form effect” we included only the two within-form comparisons of normal *vs.* deformed fish in GO analysis.

In summary, while gene expression differences between normal and deformed fish were generally weak, strong differences were found, when the two whitefish forms were compared. These strong differences may be explained by ecological and life-history differences between the Brienzlig and Albock whitefish forms (*i.e.*, spatial and temporal differences in spawning as well as feeding ecology), which require complex physiological adaptations. Additionally, the two whitefish forms were genetically distinct (see Experimental Section) and genetic divergence is well-known to affect global transcription profiles (e.g., [23,43]).

An important recent finding provides a potential explanation for the weak differences in gene expression between normal and deformed fish. In a series of long-term rearing experiments in the laboratory [11] showed, that whitefish fed with zooplankton originating from Lake Thun developed gonad deformations at comparable frequencies to wild fish regardless of genetic background (even whitefish from different lakes additionally to Lake Thun were used) and origin of water (even spring water besides water from Lake Thun was used). These results offer strong evidence that the feeding on zooplankton is directly associated with observed gonad deformations. The whitefish sampled for microarray analyses were caught on their spawning sites, *i.e.*, they have not been feeding for some time, maybe even weeks, which was clearly visible by their entirely empty intestinal tracts during the sampling period (D. Bittner; pers. observation). Therefore, we might have measured gene expression levels at a point in time, where an initial possibly stronger response had already faded away.

### 3.2. GO Analysis

Among the gene probes showing signal intensities above background, 3,449 in liver and 7,226 in head kidney had GO annotations and were consistently either up- or down-regulated in deformed compared to normal fish. In an enrichment analysis of GO categories performed on these gene sets revealed an elevated number of co-regulated genes in specific GO categories, *i.e.*, even though single genes were not significantly differently expressed, a combination or group of genes contained within GO categories showed significant difference in gonadal phenotype correlated gene expression. Significant GO categories were identified in all four analyses (*i.e.*, normal *vs.* deformed fish in Albock and Brienzlig for both tissues respectively), after *P*-value adjustment for multiple hypotheses testing (Table S4). After refinement analysis (see Material and Methods section), in both tissues significant GO terms related to the immune system were most prominent (Table 2). These were present in all four analyses (*i.e.*, in both whitefish forms and tissues respectively) with particular representation in the comparison of normal *vs.* deformed Albock in the liver where five out of seven significant GO categories were involved in immunity (Table 2a). Strong overlap of corresponding genes among the immune system categories was found. Additionally, we found strong congruencies of genes between GO categories “proteolysis” of the Albock and “extracellular region” of the Brienzlig in the liver (Table 2a, b) and between GO terms related to GTPase activation and Ras protein signal transduction in both forms in the head kidney (Table 2c, d).

Due to the structure of the gene ontology, a particular gene can be annotated in multiple GO categories simultaneously. In the liver, 252 unique genes were present among the seven significant GO terms in the Albock and 326 unique genes among the four significant GO terms in the Brienzlig out of which 102 genes were found in common between the two whitefish forms (see Figure 4a for gene identities). In the head kidney, 318 unique genes were observed among the twelve significant GO terms in the Albock and 172 unique genes among the six significant GO groups in the Brienzlig out of which again a relatively large number of genes, (71 in total, see Figure 4b for gene identities) were found in common between the two forms. These findings indicated substantial overlap in GO analysis between the two whitefish forms and both tissues, respectively.

Hierarchical clustering analysis of the four groups across the 102 common genes in liver, and the 71 shared genes in head kidney showed a separation of whitefish form rather than gonadal phenotype, *i.e.*, Albock normal clustered with Albock deformed fish and *vice versa*. This reflects the strong global gene expression differences between the two whitefish forms (Figure 4). In liver, most of the particular 102 shared genes were involved in immune system processes and a smaller number in the biological process “proteolysis” and the cellular compartment “extracellular region” (Figure 4a). In head kidney, again many of the 71 shared genes were involved in immunity, but also a large number of genes were related to the molecular function “GTPase activation” and the biological process “Ras protein signal transduction (Figure 4b).

**Figure 5:**
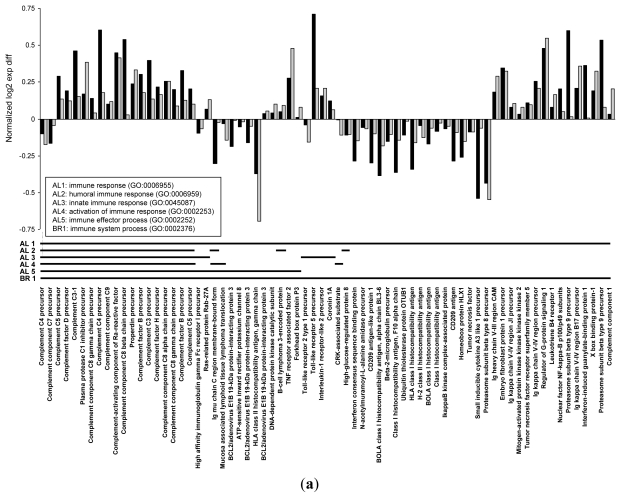

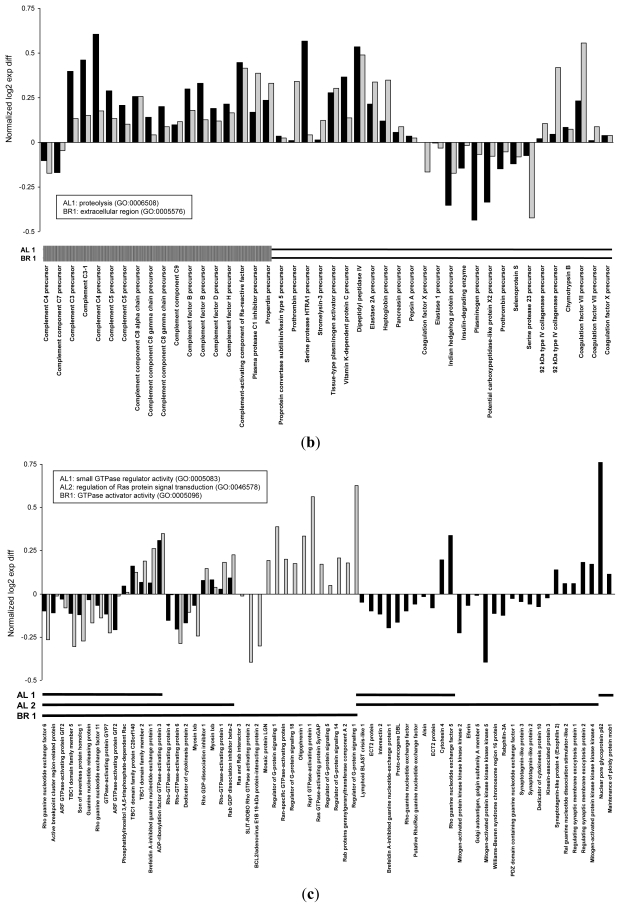

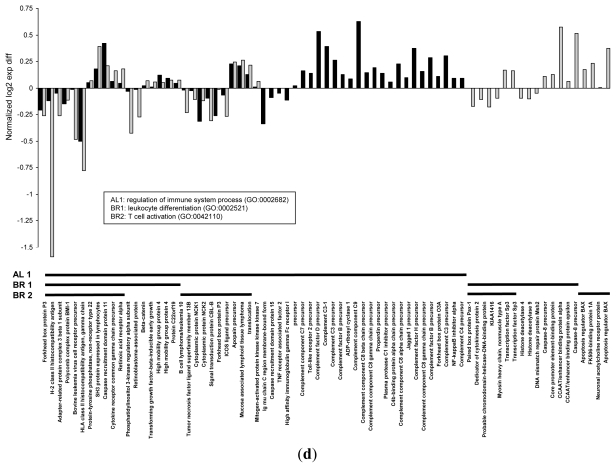
Log2 normalized gene expression differences between normal and deformed fish in Albock (AL; black) and Brienzlig (BR; grey) for shared genes among significant GO categories. (**a**) genes present in immune system related GO categories in the liver; (**b**) genes present in GO terms encoding for “proteolysis” and “extracellular region” also in the liver; (**c**) genes present in immune system related GO terms in the head kidney; and (**d**) genes present in GO categories related to GTPase activation and Ras protein signal transduction also in the head kidney. The presence or absence of a particular gene in the respective GO terms is indicated by black lines. Note that for (a) and (b) only genes present simultaneously in both whitefish forms are shown (*i.e.*, not all genes assigned to specific GO terms). Finally, in (b) the explicit marking of the first 18 genes points out that these genes are also present among the shared genes shown in (a).

In the liver 70 genes were shared among the immune system related GO categories “immune response”, “humoral immune response”, “innate immune response”, “activation of immune response” and “immune effector process” in the Albock (Table 2a), and “immune system process” in the Brienzlig (Table 2b). Among these 70 common genes, 29 were down- and 41 genes were up-regulated in deformed fish. A total of 19 genes were found in common among all six GO categories comprising both whitefish forms. These genes were primarily involved in activation of the complement system (Figure 5a). A continuous up-regulation of these genes was observed with the exception of two genes.

Another major group of immune system related genes encoded for histocompatibility antigens. Relatively large mean expression differences in both forms between normal and deformed fish were further observed for the “proteasome subunit beta type 8 precursor” gene, which plays an important role in antigen processing. All these genes were down-regulated in deformed fish of both forms. In contrast, a few immunoglobulin genes of the heavy or kappa chains were up-regulated (Figure 5a). Furthermore, in the liver, 44 genes were in common between GO terms “proteolysis” in the Albock and “extracellular region” in the Brienzlig. It is interesting that 18 of these 44 genes were in common with the 19 specific genes involved in complement activation, which is indicated in Figure 5b. Additionally, a small group of genes encoding for coagulation factors and collagenase precursors was down-regulated in deformed fish; however, 33 out of the 44 shared genes were up-regulated (Figure 5b).

Head kidney, also displayed a remarkable overlap between the two whitefish forms of genes in GO categories related to the immune system (Table 2c, d). The GO terms concerned were “regulation of immune system process” in the Albock and “leukocyte differentiation” and “T cell activation” in the Brienzlig (Table 2c, d) with 28 shared genes between the two whitefish forms (eleven genes were present in all three GO terms; Figure 5c). In contrast to genes involved in the up-regulation of the complement system in the liver, no clear pattern in the direction of gene expression regulation was observed in head kidney. However, as in the liver, two genes encoding for histocompatibility antigens were down-regulated (Figure 5c). Furthermore, in the head kidney, a second overlap of genes among significant GO categories between the Albock and Brienzlig concerned categories related to “small GTPase regulator activity” and “regulation of Ras protein signal transduction” (Table 2c, d). In more detail, a total of 22 genes were in common among the GO categories “small GTPase regulator activity” and “regulation of Ras protein signal transduction” in the Albock and “GTPase activator activity” in the Brienzlig (Figure 4d). Nine of these 22 shared genes were up- and 13 were down-regulated in deformed fish. Many of these genes encoded for GTPase activating proteins and a tendency in down-regulation of GTPase activating proteins was observed (Figure 5d).

### 3.3. Deviation in Regulation of Immune System Processes

GO analysis revealed subtle but congruent changes in gene expression patterns between normal and deformed fish of both, genetically and ecologically differentiated whitefish forms. This suggests that the observed differences in gene expression are of biological relevance and actually associated with the gonadal deformations in these fish. Significant GO categories associated primarily with the immune system and the results were consistent across both whitefish forms and tissues, respectively.

A dysregulation of immune function is commonly observed in situations where fish are stressed (e.g., [44–46]), and it thus follows that deformed fish might be more stressed than fish with normally developed gonads. However, since gene expression patterns between normal and deformed fish were rather weak and since we do not know the threshold when to consider differences as dysregulation, here, we use the neutral term “deviation in regulation”. Deviation in regulation of gene expression of immune system processes can be classified into three different major immune responses: (i) primary response of the immune system (*i.e.*, first response when organisms are exposed to exogenous factors such as pathogens or toxicants, that involve inflammatory and acute phase proteins as well as chemokines); (ii) secondary response of the immune system (*i.e.*, a response that follows at a later stage, which is an adapted and specifically directed immune response) and iii) self-directed secondary response of the immune system, *i.e.*, autoimmune response.

Microarrays have been used extensively to describe responses to pathogenic agents, *i.e.*, viral, bacterial and parasitic disease processes (as reviewed by [47]) and exposure to environmental contaminants (e.g., [19,48–50]), where deviations in regulation of gene expression patterns of immune system processes were also observed.

The most prominent finding of our analyses concerned the up-regulation of the complement system in deformed fish in the liver. The complement system is a very ancient mechanism of defense that is conserved across many species. Complement is primarily involved in host-pathogen interactions, *i.e.*, defense against bacteria, viruses, fungi and parasites. As part of the innate immune response, complement plays an important role in enhancing the uptake and processing of antigens by antigen-presenting cells. The complement system is composed of a range of soluble plasma proteins that play key roles in innate and adaptive immunity [51]. It is interesting that teleosts, unlike mammals, contain multiple isoforms of complement components, which implies novel roles in fish innate immunity [52]. Furthermore C3/C4 fragments bound to antigen or immune complexes enhance uptake and processing of antigen by antigen-presenting cells, which leads to more effective primary and secondary antibody responses to the antigen [53–55]).

Even though involvement of the complement system points towards potential roles of pathogens, thorough histological investigations have failed to demonstrate any signs of inflammation and/or infection in the gonads of deformed fish [1]. Furthermore, potential involvement of pathogens is unlikely, since the two whitefish forms were sampled independently of each other and at different locations. Such a case would be more than coincidental. Additionally, various exposure studies have always reported much stronger differences in gene expression compared to our data, where pathogens were involved (e.g., 1.8–30 fold differences in transcription of complement genes were observed by [49]. However, contrary to all exposure studies that compared exposed fish to non-exposed fish, in the present study, normal and deformed fish were sampled in the same environment, and thus had likely been equally exposed.

### 3.4. Autoimmune Disease?

Alternatively, the gene expression patterns found in immune system processes in both tissues and in particular the up-regulation of the complement system in the liver could be explained by a chronic autoimmune disease in the testis. In humans, microarrays have been widely used for the study of autoimmune diseases and specific gene expression patterns have been detected [56]. However, data is lacking as to specific hallmarks of gene expression patterns that characterize autoimmune disease, even more so in fish. But autoimmune disease often involves multiple organs [56] and specific GO categories involved in the immune system as well as additional indications in our data fit the idea of an autoimmune activation. In particular following significant GO categories were concerned: “T cell activation”, “GTPase activator activity”, “small GTPase regulator activity”, “regulation of Ras protein signal transduction”, “cell adhesion”, “cell migration”, “extracellular region” and “proteinaceous extracellular matrix” (Table 2).

In comparison with the current literature we found many interesting links of particularities of our data in the context with autoimmunity. Effective immune responses require the appropriate activation and differentiation of peripheral T cells. Defects in appropriate regulation of T cell activation underlie the pathogenesis of many autoimmune disorders in humans [57]. Even though the molecular machinery to control these processes and to prevent autoimmunity is not fully understood, activation of Rho GTPases, is recognized as a key event in the coordination of immune responses and, particularly, in the activation of T cells [57]. These recent findings are very striking in regard to our own results, since “GTPase activation”, which involved Rho GTPase activating proteins, and “T cell activation” were among the identified significant GO categories. However, Rho GTPase activating proteins are molecular switches that also control many other biological processes including complex signaling pathways as well as cytoskeletal reorganization [57].

Cell-cell and cell-matrix adhesion molecules as well as extracellular matrix components are target structures of antibody-mediated autoimmunity. Pathogenic auto-antibodies against these molecules are causally related to disturbances of cell and tissue adhesion [58]. Again our data are consistent with these findings. In liver, we identified related GO terms “extracellular region”, “proteolysis”, “cell adhesion” and “integral to plasma membrane”, whereas in head kidney GO terms “cell migration” and “cell proliferation” were present among the significant categories. Interestingly, proteins of the extracellular matrix, which is part of the extracellular region, have also been shown to play an important role in testis development in male mice [59] and specifically in rainbow trout [60]. In trout, microarray data indicated that the testicular extracellular matrix participates in processes such as growth, adhesion, differentiation, cell migration and patterning, *i.e.*, many processes, which were also evident in our data. In this regard, it is also interesting to note that in mammals the complement system has been shown to participate in gamete development and provides potential bridges through which gametes can interact [61,62]. Developmental regulation of various complement components in reproductive processes was also demonstrated in rainbow trout [60,63,64]. This is particularly interesting when considering that gonad deformations develop right with onset of gonad differentiation [11]. Therefore the gonad deformations could result from dysfunctional cell-cell interactions, *i.e.*, failure in normal cell-cell communication, during development, induced by autoimmunity. Additionally, collagen is well known to play an important role in tissue development. Our data provide a small detail further supporting the autoimmunity hypothesis, since a group of collagen genes were present in GO categories “proteolysis” (Albock) and also in “extracellular region” (Brienzlig).

By contrast, autoimmunity is commonly associated with increased expression of major histocompatibility complex (MHC) antigens and immunoglobulins [65]. This is partly incoherent with our findings, where genes encoding for histocompatibility antigens were consistently down-regulated. However, we analyzed “only” liver and head kidney tissue and not the gonads themselves, the targeted tissue of the possible autoimmune disease, where we would expect up-regulation of histocompatibility antigens according to Herskowitz *et al*. [65].

With regard to the up-regulation of genes of the complement system in the liver, it is important to mention that it is well known that complement pathway is also one of the major means by which the body recognizes tissue injury [66]. Complement activation in the course of systemic autoimmunity leads to tissue damage in a number of ways including direct lysis of cell, modification of cell function and by contributing to the formation of immune complexes [67] and thus is thought to contribute significantly to end organ damage [68]. In mammals, it has been shown that auto-antibodies in combination with intracellular antigens can trigger cell injury at cell surface by activating the complement system [67]. On the other hand, there are many mechanisms by which the complement system is activated and it is also involved in many other processes (see above).

Although rare, especially in fish, autoimmunity has previously been demonstrated, e.g., in vaccinated farmed Atlantic salmon (*Salmo salar*), where the presence of auto-antibodies was confirmed [69]. Adhesion of internal organs and tissue damage was found in all vaccinated fish in this study. Interestingly, lesions of testis characteristic of autoimmune attack were also shown in rainbow trout passively immunized with anti-sperm antibodies, which involved the presence of complement components [70]. Mature sperm (and their antigens) develop through the process of spermatogenesis, which arise only in adult organisms, *i.e.*, long after tolerance of non-specific immune system has been established. In the testis, Sertoli cells establish intercellular junctions that are essential for spermatogenesis and the blood-testis barrier is formed by tight junctions between these Sertoli cells. The presence of a blood-testis barrier is thought to provide an environment in which the appearance of specific antigens (possibly also auto-antigens) is facilitated [71]. As mentioned above, it has been demonstrated that autoimmune lesions in the testis could be induced experimentally, which, besides rainbow trout, was also the case in Atlantic salmon by injecting autologous or allogenic testis material into adult fishes [72]. These findings suggest that the blood-testis barrier may have broken down during autoimmune response to the testis, which was confirmed in the study by Lou and Takahashi [71] on Nile tilapia. The Authors provided morphological evidence for the presence of a blood-testis barrier and its break down during immune response. This indicates that the function of the blood-testis barrier may indeed be to prevent autoimmune reaction. Interestingly, it has been shown that impaired Sertoli cell junctional protein expression were the consequence of exposure to different classes of reproductive toxicants [73].

### 3.5. Indications of Ictacalcin

Studies are accumulating that autoimmune diseases are also stimulated by chronic exposure to various chemicals, *i.e.*, xenobiotic substances, in humans as well as in animals [74,75]. Our data, however, provide only a weak indication pointing to particular chemical substances. We found one gene, Ictacalcin, at the nominal 1% level that showed up-regulation in deformed fish in head kidney of both whitefish forms. Reanalysis of the samples with RT *q*PCR revealed qualitatively the same but a statistically not significant difference between deformed and normal fish in both forms. Ictacalcin is a Ca^2+^ binding protein abundant in epithelial cells of the olfactory barbells, skin, and gills of fish suggesting an important role in calcium homeostasis [76,77]. Interestingly, expression of Ictacalcin was significantly increased in juvenile rainbow trout exposed to TCDD dioxin in the head kidney, but not in liver [78], which is in line with our results. The mechanisms of the TCDD-induced increase of Ictacalcin in rainbow trout, however, were not clear. Calcium is the most common intracellular messenger in the signal transduction pathway and is required for cell growth and survival and thus plays an important role in various physiological processes. Therefore, the isolated induction of Ictacalcin alone is not conclusive that whitefish in Lake Thun are exposed in any way to dioxin-like substances. Furthermore, the GO analyses did not reveal any GO categories that are indicative for the involvement of dioxins. In this context, it also is worth noting that the array contains several probes for *CYP*1A genes, which are known to show the most sensitive response to exposure to dioxins like TCDD and which did not show any sign of up-regulation in deformed fish.

## 4. Conclusions

Our study showed that the application of gene expression profiling to study natural populations represents a promising approach to address complex ecotoxicological problems. By using such a transcriptomic approach, we identified physiological processes and one candidate gene potentially involved in gonad deformations. A deviation in regulation of gene expression of immune system processes, the most prominent finding of our data, is commonly observed in situations where fish are stressed, thus indicating, that deformed fish are under increased influence of stress. In comparison with current literature, the gene expression patterns found here best fit to a signature of an autoimmune disease in the testes. Based on the recent observations that gonad deformations are induced through feeding of zooplankton from Lake Thun, we hypothesize that a xenobiotic accumulated in whitefish via the plankton triggering autoimmunity as the likely cause of gonad deformations in Lake Thun. As a follow-up study, in order to test the autoimmunity hypothesis, we propose screen normal and deformed whitefish for auto-antibodies against gonad tissue.

## Supplementary Materials



## Figures and Tables

**Table 1:** Number and frequencies of genes showing difference in expression levels in head kidney and liver tissue observed in pairwise gene-by-gene analyses at three different nominal *P*-value thresholds, (i) 5% level; (ii) 1% level and (iii) after correcting for multiple hypothesis testing with FDR ≤ 5%. Comparisons were carried out between the following pairs of samples: (A) Albock normal *vs.* Albock deformed; (B) Brienzlig normal *vs.* Brienzlig deformed; (C) all normal (pooled Albock and Brienzlig) *vs.* all deformed and (D) all Albock (pooled normal and deformed) *vs.* all Brienzlig. The total number of expressed genes (G_EXP_) per tissue is indicated. Additionally, for the 5% and 1% levels the corresponding FDR at the respective thresholds are given in parentheses.

Liver: G_EXP_ = 7,013	Comparison	5%	FDR	1%	FDR	FDR ≤ 5%
	A	346 (0.049)	1	78 (0.011)	0.88	0
	B	74 (0.011)	1	10 (0.001)	1	0
	C	177 (0.025)	1	33 (0.005)	1	0
	D	3,407 (0.486)	0.1	2,407 (0.343)	0.03	2,764 (0.394)

**Head kidney: G_EXP_= 17,834**	**Comparison**	**5%**	**FDR**	**1%**	**FDR**	**FDR ≤ 5%**

	A	1,162 (0.065)	0.77	264 (0.015)	0.68	0
	B	2,548 (0.143)	0.35	528 (0.030)	0.31	0
	C	651 (0.037)	0.95	102 (0.006)	0.95	0
	D	12,416 (0.696)	0.01	10,382 (0.582)	0.02	12,820 (0.719)

**Table 2:** Gene Ontology (GO) categories that comprised genes that showed significant over-representation in head kidney and liver tissues at the 1% level of low *P*-value ranking among all genes after refinement analysis (see Material and Methods section) in comparisons of whitefish with deformed and normal gonads. Only GO categories with number of genes < 200 are shown.

(a)	Liver: Albock normal *vs.* Albock deformed				
	GO root node	Name	GO ID	Genes	*P*
	biological_process	immune response	GO:0006955	70	0.000
	biological_process	humoral immune response	GO:0006959	22	0.001
	biological_process	innate immune response	GO:0045087	25	0.001
	biological_process	activation of immune response	GO:0002253	22	0.002
	biological_process	Proteolysis	GO:0006508	160	0.004
	cellular_component	integral to plasma membrane	GO:0005887	60	0.009
	biological_process	immune effector process	GO:0002252	32	0.009

**(b)**	**Liver: Brienzlig normal*vs.*deformed**				
	**GO root node**	**Name**	**GO ID**	**Genes**	***P***

	cellular_component	extracellular region	GO:0005576	197	0.002
	cellular_component	proteinaceous extracellular matrix	GO:0005578	31	0.002
	biological_process	immune system process	GO:0002376	107	0.006
	biological_process	cell adhesion	GO:0007155	85	0.009

**(c)**	**Head kidney: Albock normal*vs.*deformed**				
	**GO root node**	**Name**	**GO ID**	**Genes**	***P***

	biological_process	regulation of cell proliferation	GO:0042127	114	0.000
	biological_process	positive regulation of cell proliferation	GO:0008284	54	0.000
	biological_process	embryonic development	GO:0009790	86	0.000
	biological_process	protein import into nucleus	GO:0006606	23	0.001
	molecular_function	ATP-dependent helicase activity	GO:0008026	20	0.001
	biological_process	cell migration	GO:0016477	52	0.001
	biological_process	embryonic development ending in birth or egg hatching	GO:0009792	58	0.002
	molecular_function	small GTPase regulator activity	GO:0005083	49	0.004
	biological_process	regulation of Ras protein signal transduction	GO:0046578	27	0.004
	biological_process	cellular protein complex assembly	GO:0043623	41	0.009
	biological_process	regulation of immune system process	GO:0002682	45	0.009
	biological_process	central nervous system development	GO:0007417	45	0.010

**(d)**	**Head kidney: Brienzlig normal*vs.*deformed**				
	**GO root node**	**Name**	**GO ID**	**Genes**	***P***

	molecular_function	GTPase activator activity	GO:0005096	36	0.003
	biological_process	leukocyte differentiation	GO:0002521	32	0.006
	biological_process	gamete generation	GO:0007276	40	0.006
	biological_process	T cell activation	GO:0042110	31	0.008
	cellular_component	soluble fraction	GO:0005625	32	0.009
	biological_process	muscle contraction	GO:0006936	28	0.010
