# Supplementary material for: Identification of Candidate Genes and Physiological Pathways Involved in Gonad Deformation in Whitefish (Coregonus spp.) from Lake Thun, Switzerland
Source: Int J Environ Res Public Health. 2011 Jun 30;8(7):2706–33. doi: 10.3390/ijerph8072706 (PMC3155325; doi:10.3390/ijerph8072706)
Supplement: Supplementary file 1 [file ijerph-08-02706-s001.doc]

Int. J. Environ. Res. Public Health **2011**, 8, 1-x manuscripts; doi:10.3390/ijerph80x000x

**OPEN ACCESS**

International Journal of

Environmental Research and
Public Health
ISSN 1660-4601
www.mdpi.com/journal/ijerph

*Article*

Identification of Candidate Genes and Physiological Pathways Involved in Gonad Deformation in Whitefish (*Coregonus* spp.) from Lake Thun, Switzerland

David Bittner ^1^, Andrew R. Cossins ^2^, Helmut Segner ^3^, Laurent Excoffier ^1^
and Carlo R. Largiadèr ^4^,*

^1^ Computational and Molecular Populations Genetics Lab, University of Bern, Baltzerstrasse 6, 3012 Bern, Switzerland; E-Mails: David.Bittner@eawag.ch (D.B.); laurent.excoffier@iee.unibe.ch (L.E.)

^2^ Liverpool Microarray Facility, School of Biological Sciences, University of Liverpool, L69 7ZB Liverpool, UK; E-Mail: cossins@liverpool.ac.uk

^3^ Centre for Fish and Wildlife Health, University of Bern, Laenggass-Strasse 122, PO-Box 8466, 3001 Bern, Switzerland; E-Mail: helmut.segner@itpa.unibe.ch

^4^ Institute of Clinical Chemistry, University Hospital, University of Bern, Inselspital, CH-3010 Bern, Switzerland

* Author to whom correspondence should be addressed; E-Mail: carlo.largiader@insel.ch; cossins@liverpool.ac.uk; Tel.: +41-31-632-9545; Fax: +41-31-632-0310.

**Supplemental Material**

**Table S1.** Mean (*x*) and standard deviation (σ) of gill raker numbers, weight (g), length (mm), age (years), assignment probabilities *P* and sample size (n) of fish used in the array experiments. Data are given for the two whitefish forms Brienzlig (BR) and Albock (AL) and are further stratified by gonad morphology (normal/deformed). Differences between means of the various parameters among the two forms as well as within forms based on gonad morphology were evaluated using *t*-tests (Sokal & Rohlf 1981, p. 223) and are indicated by *P*-values (*p*).

|  | **BR** | **AL** | ***p*** | **BR_def** | **BR_nor** | ***p*** | **AL_def** | **AL_nor** | ***p*** |
| --- | --- | --- | --- | --- | --- | --- | --- | --- | --- |
| gill rakers *x* | 35.57 | 37.48 | 0.01 | 37.40 | 37.57 | 0.88 | 35.64 | 35.50 | 0.84 |
| gill rakers σ | 1.85 | 2.95 |  | 3.07 | 2.93 |  | 1.95 | 1.83 |  |
|  |  |  |  |  |  |  |  |  |  |
| weight *x* | 289.68 | 167.66 | 0.00 | 158.53 | 177.43 | 0.07 | 299.43 | 279.93 | 0.31 |
| weight σ | 49.29 | 28.03 |  | 21.73 | 31.38 |  | 65.11 | 24.55 |  |

**Table S1.** *Cont.*

|  | **BR** | **AL** | ***p*** | **BR_def** | **BR_nor** | ***p*** | **AL_def** | **AL_nor** | ***p*** |
| --- | --- | --- | --- | --- | --- | --- | --- | --- | --- |
| length *x* | 326.89 | 267.62 | 0.00 | 264.40 | 271.07 | 0.19 | 331.07 | 322.71 | 0.22 |
| length σ | 17.59 | 13.56 |  | 11.82 | 14.85 |  | 22.42 | 10.13 |  |
|  |  |  |  |  |  |  |  |  |  |
| age *x* | 3.14 | 3.76 | 0.00 | 3.73 | 3.79 | 0.79 | 3.14 | 3.14 | 1.00 |
| age σ | 0.36 | 0.51 |  | 0.59 | 0.43 |  | 0.36 | 0.36 |  |
|  |  |  |  |  |  |  |  |  |  |
| *P* BR *x* | 0.05 | 0.40 | 0.00 | 0.40 | 0.39 | 0.91 | 0.10 | 0.01 | 0.81 |
| *P* BR σ | 0.14 | 0.29 |  | 0.33 | 0.25 |  | 0.19 | 0.02 |  |
|  |  |  |  |  |  |  |  |  |  |
| *P* Al *x* | 0.38 | 0.10 | 0.00 | 0.12 | 0.08 | 0.65 | 0.35 | 0.41 | 0.60 |
| *P* Al σ | 0.29 | 0.17 |  | 0.23 | 0.06 |  | 0.25 | 0.33 |  |

**Table S2.** Evaluation of rainbow trout oligonucleotide probes for use with whitefish extracted total RNA. Given is the number of probes at two thresholds for which expression was detected among 21’492 probes before and after global normalization (norglo). Raw intensities were log2 transformed.

| **tissue** | **threshold** | **whitefish** | **whitefish nor_glo_** | **rainbow** | **rainbow nor_glo_** | **both  species** | **both species nor_glo_** |
| --- | --- | --- | --- | --- | --- | --- | --- |
| liver | > 7 | 4636 | 3616 | 2246 | 3072 | 5170 | 4582 |
|  | > 9 | 1398 | 951 | 521 | 791 | 1563 | 1285 |
| head kidney | > 7 | 4903 | 4311 | 3925 | 4519 | 5663 | 5540 |
|  | > 9 | 1257 | 1055 | 888 | 1057 | 1452 | 1386 |
| both tissues | > 7 | 5696 | 4897 | 4503 | 5352 | 6699 | 6590 |
|  | > 9 | 1661 | 1296 | 1077 | 1364 | 1948 | 1840 |

**Table S3.** Gene-by-gene analysis between normal and deformed whitefish based on ANOVA between following combinations of the four population groups: (a) and (d) Albock normal *vs.* Albock deformed; (b) and (e) Brienzlig normal *vs.* Brienzlig deformed; (c) and (f) all normal fish (Albock and Brienzlig pooled) *vs.* all deformed fish for
liver (a,b,c) and head kidney (d,e,f) at the 0.01 level. Given are gene identities (Gene ID), sample sizes (N), mean log2 intensity measurements (x) for both respective Population groups (Pop1 and 2 as indicated in header) along with *P*-values (P) and corresponding false discovery rate (FDR).

| **(a) Liver: Albock deformed (Pop1) *vs.* Albock normal (Pop2)** |  |  |  |  |  |  |  |
| --- | --- | --- | --- | --- | --- | --- | --- |
| **Gene ID** | **Tigr ID** | **N_1_** | **N_2_** | **x_1_** | **x_2_** | ***P*** | **FDR** |
| NGD5 protein homolog (CGI-53) | BX869040 | 14 | 14 | 7.413 | 7.748 | 0.000 | 0.701 |
| glutathione peroxidase type 2 [Oncorhynchus mykiss] | AY622862 | 14 | 14 | 10.119 | 9.857 | 0.002 | 0.810 |
| Protein C22orf2 (Cytosolic leucine-rich protein) | TC69794 | 14 | 14 | 8.601 | 8.915 | 0.002 | 0.810 |
| Mu-crystallin homolog | TC90482 | 14 | 14 | 7.804 | 7.068 | 0.002 | 0.810 |
| NifU-like protein | TC78409 | 14 | 14 | 11.884 | 11.459 | 0.002 | 0.810 |
| PREDICTED: similar to Protein KIAA0020 [Gallus gallus] | BX874752 | 14 | 14 | 7.585 | 7.230 | 0.002 | 0.810 |

**Table S3.** *Cont.*

| **(a) Liver: Albock deformed (Pop1) *vs.* Albock normal (Pop2)** |  |  |  |  |  |  |  |
| --- | --- | --- | --- | --- | --- | --- | --- |
| Calmodulin | TC86520 | 14 | 14 | 9.660 | 9.215 | 0.002 | 0.810 |
| zgc:77146 [Danio rerio] | TC88396 | 14 | 14 | 9.745 | 9.959 | 0.002 | 0.810 |
| Cyclic-AMP-dependent transcription factor ATF-5 | BX085471 | 14 | 14 | 7.961 | 8.424 | 0.002 | 0.810 |
| Zinc finger protein 16 (Zinc finger protein KOX9) | TC71695 | 14 | 14 | 7.881 | 8.310 | 0.003 | 0.810 |
| N-acetyltransferase 5 (EC 2.3.1.-) | TC88365 | 14 | 14 | 9.077 | 9.303 | 0.003 | 0.810 |
| Leptin receptor overlapping transcript-like 1 (My047 | CA384590 | 14 | 14 | 7.275 | 7.534 | 0.003 | 0.810 |
| unnamed protein product [Tetraodon nigroviridis] | TC70678 | 14 | 14 | 7.877 | 7.550 | 0.003 | 0.810 |
| myosin regulatory light chain interacting protein; wu:fj36b03 | TC77315 | 14 | 14 | 7.163 | 6.955 | 0.003 | 0.810 |
| Leukocyte elastase inhibitor (LEI) (Monocyte/neutrophil | TC74201 | 14 | 14 | 8.543 | 8.174 | 0.003 | 0.810 |
| PREDICTED: similar to KIAA0685 protein, partial [Gallus gallus] | TC71299 | 14 | 14 | 8.940 | 9.234 | 0.003 | 0.810 |
| Proteasome subunit beta type 7 precursor (EC 3.4.25.1) | BX073710 | 14 | 14 | 8.872 | 9.238 | 0.003 | 0.810 |
| NifU-like protein | CX031661 | 14 | 14 | 8.575 | 8.208 | 0.003 | 0.810 |
| Fatty acid-binding protein, liver (L-FABP) (Liver basic | CA385329 | 14 | 14 | 12.684 | 11.986 | 0.004 | 0.810 |
| GTP cyclohydrolase I (EC 3.5.4.16) (GTP-CH-I) (Fragment) | NP544134 | 14 | 14 | 7.641 | 8.310 | 0.004 | 0.810 |
| low molecular mass polypeptide complex subunit 2 [Oncorhynchus | AF112117 | 14 | 14 | 10.380 | 9.809 | 0.004 | 0.810 |
| Orphan nuclear receptor SHP (Small heterodimer partner) | TC75200 | 14 | 14 | 8.230 | 9.177 | 0.004 | 0.810 |
| Putative MAP kinase activating protein C22orf5 | TC91503 | 14 | 14 | 7.136 | 7.438 | 0.004 | 0.810 |
| Leukocyte elastase inhibitor (LEI) (Leucocyte neutral | CX136141 | 14 | 14 | 7.875 | 7.574 | 0.004 | 0.810 |
| Complement C4 precursor [Contains: C4a anaphylatoxin] | BX909258 | 14 | 14 | 7.448 | 6.843 | 0.004 | 0.810 |
| unnamed protein product [Tetraodon nigroviridis] | TC75213 | 14 | 14 | 10.621 | 10.142 | 0.004 | 0.810 |
| Fatty acid-binding protein, liver (L-FABP) (Liver basic | TC69944 | 14 | 14 | 13.216 | 12.563 | 0.004 | 0.810 |
| Proteasome subunit beta type 9 precursor (EC 3.4.25.1) | TC72410 | 14 | 14 | 8.528 | 7.929 | 0.004 | 0.810 |
| unnamed protein product [Tetraodon nigroviridis] | CX037322 | 14 | 14 | 7.059 | 6.663 | 0.004 | 0.810 |
| Transcriptional repressor NF-X1 (EC 6.3.2.-) (Nuclear | CA358863 | 14 | 14 | 8.043 | 7.912 | 0.005 | 0.810 |
| Translationally-controlled tumor protein (TCTP) | TC78402 | 14 | 14 | 13.091 | 13.652 | 0.005 | 0.810 |
| Glutathione peroxidase-gastrointestinal (EC 1.11.1.9) | CA345885 | 14 | 14 | 8.991 | 8.768 | 0.005 | 0.810 |
| UTP--glucose-1-phosphate uridylyltransferase 2 (EC 2.7.7.9) | TC72127 | 14 | 14 | 9.171 | 8.355 | 0.005 | 0.810 |
| unnamed protein product [Tetraodon nigroviridis] | TC89071 | 14 | 14 | 8.954 | 9.108 | 0.005 | 0.810 |
| unnamed protein product [Tetraodon nigroviridis] | CX030754 | 14 | 14 | 7.434 | 7.710 | 0.005 | 0.810 |
| Id1 protein [Oncorhynchus mykiss] | AY842405 | 14 | 14 | 6.881 | 7.409 | 0.005 | 0.810 |
| DNA-binding protein inhibitor ID-1 (ID) | TC80123 | 14 | 14 | 7.138 | 7.904 | 0.005 | 0.810 |
| Fatty acid-binding protein, liver (L-FABP) (Liver basic | TC69943 | 14 | 14 | 12.229 | 11.604 | 0.005 | 0.810 |
| Microsomal glutathione S-transferase 3 (EC 2.5.1.18) | TC71784 | 14 | 14 | 11.256 | 10.706 | 0.005 | 0.810 |
| SH3-domain kinase binding protein 1 (SH3 containing, | TC88024 | 14 | 14 | 7.013 | 6.501 | 0.005 | 0.810 |
| Interferon consensus sequence binding protein (ICSBP) | TC90441 | 14 | 14 | 6.868 | 7.155 | 0.005 | 0.810 |
| Potential carboxypeptidase-like protein X2 precursor | TC88461 | 14 | 14 | 8.180 | 8.515 | 0.006 | 0.810 |
| UTP--glucose-1-phosphate uridylyltransferase 2 (EC | CA387781 | 14 | 14 | 9.035 | 8.320 | 0.006 | 0.810 |
| Glutathione peroxidase-gastrointestinal (EC 1.11.1.9) | TC69629 | 14 | 14 | 9.310 | 8.956 | 0.006 | 0.810 |
| Myosin light polypeptide 6 (Myosin light chain alkali 3) | TC70741 | 14 | 14 | 7.720 | 7.285 | 0.006 | 0.810 |
| complement C4 [Oncorhynchus mykiss] | AJ544262 | 14 | 14 | 12.091 | 11.316 | 0.006 | 0.810 |
| unnamed protein product [Tetraodon nigroviridis] | TC71046 | 14 | 14 | 8.028 | 8.348 | 0.006 | 0.810 |
| Histone acetyltransferase HTATIP (EC 2.3.1.48) (60 kDa Tat | CA371737 | 14 | 14 | 7.014 | 7.368 | 0.006 | 0.810 |
| UTP--glucose-1-phosphate uridylyltransferase 2 (EC | TC84586 | 14 | 14 | 8.546 | 7.812 | 0.006 | 0.810 |
| unnamed protein product [Tetraodon nigroviridis] | TC75588 | 14 | 14 | 11.000 | 11.856 | 0.006 | 0.810 |
| Proteasome subunit beta type 9 precursor (EC 3.4.25.1) | TC79321 | 14 | 14 | 9.913 | 9.241 | 0.006 | 0.810 |
| Acylphosphatase, muscle type isozyme (EC 3.6.1.7) | CX027944 | 14 | 14 | 8.019 | 7.425 | 0.006 | 0.810 |

**Table S3.** *Cont.*

| **(a) Liver: Albock deformed (Pop1) *vs.* Albock normal (Pop2)** |  |  |  |  |  |  |  |
| --- | --- | --- | --- | --- | --- | --- | --- |
| Plasminogen precursor (EC 3.4.21.7) | TC83945 | 14 | 14 | 7.477 | 7.923 | 0.006 | 0.810 |
| Orphan nuclear receptor SHP (Small heterodimer partner) | CA381388 | 14 | 14 | 6.597 | 7.338 | 0.006 | 0.810 |
| RGM-like protein; wu:fb38f10 [Danio rerio] | BX080242 | 14 | 14 | 7.695 | 7.983 | 0.007 | 0.810 |
| LDL receptor 2 precursor - African clawed frog | AB218827 | 14 | 14 | 7.192 | 6.820 | 0.007 | 0.810 |
| Aspartyl aminopeptidase (EC 3.4.11.21) | TC70333 | 14 | 14 | 7.118 | 6.830 | 0.007 | 0.810 |
| Cathepsin F precursor (EC 3.4.22.41) | TC83302 | 14 | 14 | 13.978 | 14.417 | 0.007 | 0.810 |
| ADAM 22 precursor (A disintegrin and metalloproteinase | CA363158 | 14 | 14 | 7.595 | 8.200 | 0.008 | 0.869 |
| Cathepsin H precursor (EC 3.4.22.16) | TC70626 | 14 | 14 | 8.907 | 8.485 | 0.008 | 0.869 |
| Proteasome subunit beta type 9 precursor (EC 3.4.25.1) | CA365139 | 14 | 14 | 8.331 | 7.797 | 0.008 | 0.869 |
| LDL receptor adaptor protein [Homo sapiens] | TC73163 | 14 | 14 | 8.143 | 8.494 | 0.008 | 0.869 |
| complement component C8 beta [Oncorhynchus mykiss] | AF418597 | 14 | 14 | 14.312 | 13.738 | 0.008 | 0.869 |
| Fatty acid synthase (EC 2.3.1.85) [Includes: EC 2.3.1.38; EC | CA361413 | 14 | 14 | 10.779 | 10.184 | 0.008 | 0.869 |
| DNA-binding protein inhibitor ID-1 (ID) | TC70606 | 14 | 14 | 7.291 | 7.968 | 0.008 | 0.869 |
| Ran GTPase-activating protein 1 | TC82585 | 14 | 14 | 8.851 | 9.127 | 0.009 | 0.869 |
| unnamed protein product [Tetraodon nigroviridis] | BX888058 | 14 | 14 | 7.443 | 7.278 | 0.009 | 0.869 |
| THO complex subunit 3 (Tho3) | TC88378 | 14 | 14 | 7.715 | 7.474 | 0.009 | 0.869 |
| Orphan nuclear receptor SHP (Small heterodimer partner) | BX909735 | 14 | 14 | 6.795 | 7.259 | 0.009 | 0.869 |
| Leukocyte elastase inhibitor (LEI) | CR373314 | 14 | 14 | 7.224 | 6.957 | 0.009 | 0.869 |
| F-box/LRR-repeat protein 3A (F-box and leucine-rich repeat | TC82503 | 14 | 14 | 7.088 | 6.418 | 0.009 | 0.869 |
| Nop14-like; ik:tdsubc_1a2; xx:tdsubc_1a2 [Danio rerio] | TC71767 | 14 | 14 | 9.307 | 8.946 | 0.009 | 0.877 |
| Beta-microseminoprotein A1 precursor (msp-A1) (Fragment) | TC76641 | 14 | 14 | 11.370 | 10.864 | 0.009 | 0.877 |
| similar to hypothetical protein MGC27016 [Rattus norvegicus] | TC94038 | 14 | 14 | 7.660 | 7.299 | 0.009 | 0.877 |
| unnamed protein product [Tetraodon nigroviridis] | TC87544 | 14 | 14 | 12.750 | 12.316 | 0.010 | 0.877 |
| Translationally-controlled tumor protein (TCTP) | TC78401 | 14 | 14 | 13.981 | 14.335 | 0.010 | 0.877 |
| PREDICTED: similar to RIKEN cDNA 2510005D08 [Gallus gallus] | TC79594 | 14 | 14 | 8.088 | 7.783 | 0.010 | 0.877 |
| low molecular mass protein 2 [Oncorhynchus mykiss] | AF115541 | 14 | 14 | 9.958 | 9.440 | 0.010 | 0.877 |
|  |  |  |  |  |  |  |  |
| **(b) Liver: Brienzlig deformed (Pop1) *vs.* Brienzlig normal (Pop2)** |  |  |  |  |  |  |  |
| **Gene ID** | **Tigr ID** | **N_1_** | **N_2_** | **x_1_** | **x_2_** | ***P*** | **FDR** |
| 92 kDa type IV collagenase precursor (EC 3.4.24.35) (92 kDa | TC78734 | 14 | 14 | 9.943 | 9.523 | 0.001 | 1 |
| Noelin 2 precursor (Olfactomedin 2) | CR371709 | 14 | 14 | 10.356 | 9.765 | 0.002 | 1 |
| Microfibril-associated glycoprotein 4 precursor | TC88635 | 14 | 14 | 9.428 | 7.954 | 0.004 | 1 |
| MHC class II alpha [Oncorhynchus mykiss] | AJ251432 | 14 | 14 | 7.163 | 8.076 | 0.004 | 1 |
| HLA class II histocompatibility antigen, gamma chain | TC70179 | 14 | 14 | 9.434 | 10.128 | 0.005 | 1 |
| Circulating cathodic antigen (CCA) | TC70773 | 14 | 14 | 7.110 | 7.338 | 0.006 | 1 |
| 60S ribosomal protein L37a | TC78139 | 14 | 14 | 7.515 | 7.188 | 0.006 | 1 |
| Ras-like protein rasD (Transforming protein P23) | BX076836 | 14 | 14 | 7.085 | 7.389 | 0.008 | 1 |
| invariant chain INVX [Oncorhynchus mykiss] | AY065837 | 14 | 14 | 8.872 | 9.585 | 0.009 | 1 |
| hypothetical protein MGC55835 [Danio rerio] | TC85863 | 14 | 14 | 7.137 | 7.399 | 0.009 | 1 |
| Cyclic-AMP-dependent transcription factor ATF-5 | BX085471 | 28 | 28 | 8.092 | 8.465 | 0.001 | 1 |
| PREDICTED: similar to CXYorf1-related protein [Gallus gallus] | CR369505 | 28 | 28 | 7.927 | 8.097 | 0.001 | 1 |
| NifU-like protein | TC78409 | 28 | 28 | 11.835 | 11.468 | 0.002 | 1 |
| similar to mitochondrial ribosomal protein L16 (L16mt) [Rattus | TC87766 | 28 | 28 | 9.104 | 8.304 | 0.002 | 1 |
| Zinc finger protein 16 (Zinc finger protein KOX9) | TC71695 | 28 | 28 | 8.107 | 8.455 | 0.002 | 1 |
| G/T mismatch-specific thymine DNA glycosylase (EC 3.2.2.-) | TC84068 | 28 | 28 | 8.484 | 8.865 | 0.002 | 1 |

**Table S3.** *Cont.*

| **(c) Liver: all deformed (pooled Albock and Brienzlig) *vs.* all normal** |  |  |  |  |  |  |  |
| --- | --- | --- | --- | --- | --- | --- | --- |
| **Gene ID** | **Tigr ID** | **N_1_** | **N_2_** | **x_1_** | **x_2_** | ***P*** | **FDR** |
| Fucose operon fucU protein | TC72951 | 28 | 28 | 9.177 | 9.453 | 0.002 | 1 |
| Zinc-binding protein A33 | BX306375 | 28 | 28 | 5.732 | 6.565 | 0.002 | 1 |
| HLA class II histocompatibility antigen, gamma chain | TC70179 | 28 | 28 | 9.566 | 10.099 | 0.002 | 1 |
| Fucose operon fucU protein | CR376295 | 28 | 28 | 8.762 | 9.024 | 0.003 | 1 |
| RIKEN cDNA 2610301B20; EST AI428449 [Mus musculus] | CA384596 | 28 | 28 | 7.092 | 7.327 | 0.004 | 1 |
| hypothetical protein [Homo sapiens] | TC78786 | 28 | 28 | 7.949 | 8.114 | 0.004 | 1 |
| Noelin 2 precursor (Olfactomedin 2) | CR371709 | 28 | 28 | 10.335 | 9.974 | 0.004 | 1 |
| Protein C22orf2 (Cytosolic leucine-rich protein) | TC69794 | 28 | 28 | 8.743 | 8.984 | 0.004 | 1 |
| invariant chain INVX [Oncorhynchus mykiss] | AY065837 | 28 | 28 | 9.016 | 9.532 | 0.004 | 1 |
| unnamed protein product [Tetraodon nigroviridis] | CA365227 | 28 | 28 | 8.109 | 8.303 | 0.005 | 1 |
| Leukocyte elastase inhibitor (LEI) | CR373314 | 28 | 28 | 7.151 | 6.940 | 0.005 | 1 |
| Myosin light polypeptide 6 (Myosin light chain alkali 3) | TC70741 | 28 | 28 | 7.768 | 7.437 | 0.005 | 1 |
| PREDICTED: similar to CXYorf1-related protein [Gallus gallus] | BX870905 | 28 | 28 | 8.317 | 8.468 | 0.005 | 1 |
| NifU-like protein | CX031661 | 28 | 28 | 8.428 | 8.135 | 0.005 | 1 |
| Uncharacterized hematopoietic stem/progenitor cells | TC88497 | 28 | 28 | 8.701 | 8.962 | 0.006 | 1 |
| Leptin receptor overlapping transcript-like 1 (My047 | CA384590 | 28 | 28 | 7.325 | 7.472 | 0.006 | 1 |
| similar to hypothetical protein MGC27016 [Rattus norvegicus] | TC94038 | 28 | 28 | 7.588 | 7.318 | 0.006 | 1 |
| Transcriptional repressor NF-X1 (EC 6.3.2.-) (Nuclear | CA358863 | 28 | 28 | 8.109 | 7.981 | 0.006 | 1 |
| 60S ribosomal protein L37a | TC78139 | 28 | 28 | 7.561 | 7.315 | 0.007 | 1 |
| GTP cyclohydrolase I (EC 3.5.4.16) (GTP-CH-I) (Fragment) | NP544134 | 28 | 28 | 7.700 | 8.081 | 0.007 | 1 |
| Lymphocyte cytosolic protein 2 (SH2 domain-containing | TC73842 | 28 | 28 | 7.710 | 7.984 | 0.008 | 1 |
| unnamed protein product [Tetraodon nigroviridis] | TC71046 | 28 | 28 | 8.181 | 8.374 | 0.008 | 1 |
| complement C4 [Oncorhynchus mykiss] | AJ544262 | 28 | 28 | 11.854 | 11.197 | 0.008 | 1 |
| ARP2/3 complex 16 kDa subunit (p16-ARC) (Actin-related | TC93444 | 28 | 28 | 8.548 | 8.693 | 0.009 | 1 |
| Kynurenine/alpha-aminoadipate aminotransferase | CR373149 | 28 | 28 | 15.581 | 15.769 | 0.010 | 1 |
| MHC class II invariant chain-like protein 1 [Oncorhynchus mykiss] | AY081776 | 28 | 28 | 7.053 | 7.385 | 0.010 | 1 |
| unnamed protein product [Tetraodon nigroviridis] | CR375264 | 28 | 28 | 8.148 | 7.588 | 0.010 | 1 |
|  |  |  |  |  |  |  |  |
| **(d) Kidney: Albock deformed (Pop1) *vs.* Albock normal (Pop2)** |  |  |  |  |  |  |  |
| **Gene ID** | **Tigr ID** | **N_1_** | **N_2_** | **x_1_** | **x_2_** | ***P*** | **FDR** |
| hypothetical protein FLJ36874 [Homo sapiens] | BX873628 | 14 | 14 | 6.665 | 7.018 | 0.000 | 0.549 |
| mKIAA1844 protein [Mus musculus] | TC94498 | 14 | 14 | 5.795 | 6.083 | 0.000 | 0.549 |
| Frizzled-related protein precursor (Frzb-1) (Frezzled) | TC93175 | 14 | 14 | 6.002 | 5.468 | 0.000 | 0.549 |
| unnamed protein product [Tetraodon nigroviridis] | BX887051 | 14 | 14 | 5.991 | 6.369 | 0.000 | 0.549 |
| Amidophosphoribosyltransferase precursor (EC 2.4.2.14) | TC85980 | 14 | 14 | 8.152 | 8.425 | 0.000 | 0.549 |
| Rho GTPase activating protein 27 [Homo sapiens] | CX029327 | 14 | 14 | 9.405 | 9.660 | 0.000 | 0.549 |
| Interferon consensus sequence binding protein (ICSBP) | TC90441 | 14 | 14 | 10.670 | 11.289 | 0.000 | 0.549 |
| Zgc:101097 protein [Danio rerio] | TC93812 | 14 | 14 | 9.736 | 9.294 | 0.000 | 0.549 |
| Forkhead box protein J3 | TC84177 | 14 | 14 | 12.882 | 13.226 | 0.000 | 0.549 |
| limb and neural patterns; lunapark [Mus musculus] | TC88676 | 14 | 14 | 5.717 | 6.362 | 0.000 | 0.549 |
| UDP-GlcNAc:betaGal beta-1,3-N-acetylglucosaminyltransferase 4 [Mus | CR372465 | 14 | 14 | 6.894 | 7.971 | 0.000 | 0.549 |
| CREB-binding protein (EC 2.3.1.48) | TC89694 | 14 | 14 | 6.173 | 6.882 | 0.000 | 0.549 |
| Translationally-controlled tumor protein (TCTP) | TC78401 | 11 | 13 | 17.253 | 17.783 | 0.000 | 0.549 |

**Table S3.** *Cont.*

| **(d) Kidney: Albock deformed (Pop1) *vs.* Albock normal (Pop2)** |  |  |  |  |  |  |  |
| --- | --- | --- | --- | --- | --- | --- | --- |
| **Gene ID** | **Tigr ID** | **N_1_** | **N_2_** | **x_1_** | **x_2_** | ***P*** | **FDR** |
| Claudin-6 (Skullin 2) (UNQ757/PRO1488) | TC88355 | 14 | 14 | 5.552 | 6.311 | 0.000 | 0.637 |
| Receptor activity-modifying protein 1 precursor | BX863135 | 14 | 14 | 8.810 | 7.842 | 0.001 | 0.594 |
| Acyl carrier protein, mitochondrial precursor (ACP) | TC70158 | 14 | 14 | 8.225 | 7.640 | 0.001 | 0.594 |
| Kruppel-like factor 2b [Danio rerio] | CA371407 | 14 | 14 | 10.583 | 11.067 | 0.001 | 0.594 |
| Translationally-controlled tumor protein (TCTP) | TC78402 | 14 | 14 | 16.281 | 17.002 | 0.001 | 0.594 |
| unnamed protein product [Tetraodon nigroviridis] | BX082162 | 14 | 14 | 11.625 | 12.119 | 0.001 | 0.611 |
| FLJ00180 protein [Homo sapiens] | BX321530 | 14 | 14 | 9.211 | 9.497 | 0.001 | 0.611 |
| Ubiquitin carboxyl-terminal hydrolase 37 (EC 3.1.2.15) | TC86074 | 14 | 14 | 5.600 | 5.933 | 0.001 | 0.611 |
| Mitochondrial 28S ribosomal protein S36 (S36mt) (MRP-S36) | TC87060 | 14 | 14 | 10.134 | 9.849 | 0.001 | 0.611 |
| Tetratricopeptide repeat protein 9 (TPR repeat protein 9) | TC71416 | 14 | 14 | 10.996 | 10.829 | 0.001 | 0.611 |
| V-type ATPase B subunit [Oncorhynchus mykiss] | AF140022 | 14 | 14 | 9.705 | 10.124 | 0.001 | 0.611 |
| Probable glutamate receptor precursor (Kainate-binding | TC90372 | 14 | 14 | 7.815 | 7.507 | 0.001 | 0.611 |
| Serine/threonine-protein kinase PRP4 homolog (EC 2.7.1.37) | TC76257 | 14 | 14 | 6.004 | 6.320 | 0.001 | 0.611 |
| Putative HTH-type transcriptional regulator yafC | CA380345 | 14 | 14 | 5.886 | 5.674 | 0.001 | 0.611 |
| Steroid hormone receptor ERR2 (Estrogen-related receptor, | CA366558 | 14 | 14 | 7.737 | 7.284 | 0.001 | 0.611 |
| membrane bound immunoglobulin [Oncorhynchus mykiss] | X65263 | 14 | 14 | 14.160 | 14.608 | 0.001 | 0.611 |
| unnamed protein product [Tetraodon nigroviridis] | CA386395 | 14 | 14 | 8.863 | 9.049 | 0.001 | 0.611 |
| unnamed protein product [Tetraodon nigroviridis] | TC92247 | 14 | 14 | 7.262 | 7.722 | 0.001 | 0.611 |
| Spectrin beta chain, brain 1 (Spectrin, non-erythroid beta | TC89216 | 14 | 14 | 6.111 | 6.363 | 0.001 | 0.611 |
| RPE-retinal G protein-coupled receptor | TC91491 | 14 | 14 | 5.520 | 6.110 | 0.001 | 0.611 |
| Circulating cathodic antigen (CCA) | TC70773 | 14 | 14 | 9.625 | 9.265 | 0.001 | 0.611 |
| DNA (cytosine-5)-methyltransferase 1 (EC 2.1.1.37) (Dnmt1) | CX034411 | 14 | 14 | 6.547 | 6.835 | 0.001 | 0.611 |
| T-cell ecto-ADP-ribosyltransferase 1 precursor (EC | CA380561 | 14 | 14 | 9.232 | 8.853 | 0.001 | 0.611 |
| Nuclear pore glycoprotein p62 (62 kDa nucleoporin) | TC71079 | 14 | 14 | 6.371 | 5.455 | 0.001 | 0.611 |
| socius [Homo sapiens] | TC81794 | 14 | 14 | 5.904 | 5.720 | 0.001 | 0.611 |
| Nuclear pore complex protein Nup98 (Nucleoporin Nup98) (98 | TC75931 | 14 | 14 | 5.544 | 5.747 | 0.001 | 0.611 |
| Plexin A3 precursor (Plexin 4) (Transmembrane protein sex) | CA382802 | 14 | 14 | 8.862 | 9.205 | 0.001 | 0.611 |
| PREDICTED: similar to Chain L, Crystal Structure Of The Fab | TC78002 | 14 | 14 | 11.589 | 9.144 | 0.002 | 0.611 |
| Glutathione S-transferase P (EC 2.5.1.18) (GST class-pi) | TC69719 | 14 | 14 | 5.817 | 5.588 | 0.002 | 0.611 |
| Kruppel-like factor 2b [Danio rerio] | TC83724 | 14 | 14 | 9.526 | 9.995 | 0.002 | 0.611 |
| Disabled homolog 2 (Differentially expressed protein 2) | CA352169 | 14 | 14 | 7.463 | 7.036 | 0.002 | 0.611 |
| B-cell lymphoma/leukemia 11B (B-cell CLL/lymphoma 11B) | CR375489 | 14 | 14 | 8.068 | 8.319 | 0.002 | 0.611 |
| EH-domain containing protein 3 | TC69683 | 14 | 14 | 12.556 | 12.925 | 0.002 | 0.611 |
| Carboxypeptidase D precursor (EC 3.4.17.-) (GP180) | CA343654 | 14 | 14 | 9.235 | 8.582 | 0.002 | 0.611 |
| hypothetical protein MGC63674 [Danio rerio] | TC90973 | 14 | 14 | 6.328 | 5.740 | 0.002 | 0.611 |
| similar to KIAA0174 gene product; wu:fb16b04 [Danio rerio] | TC87284 | 14 | 14 | 8.126 | 8.421 | 0.002 | 0.611 |
| Ikaros homolog [Oncorhynchus mykiss] | U92200 | 14 | 14 | 13.252 | 13.495 | 0.002 | 0.611 |
| DNA-binding protein Ikaros | NP544110 | 14 | 14 | 13.226 | 13.463 | 0.002 | 0.611 |
| PREDICTED: hypothetical protein XP_143246 [Mus musculus] | BX858772 | 14 | 14 | 8.832 | 8.407 | 0.002 | 0.611 |
| Protocadherin gamma A12 precursor (PCDH-gamma-A12) | BX873940 | 14 | 14 | 6.408 | 6.079 | 0.002 | 0.611 |
| Stonustoxin beta-subunit (SNTX beta-subunit) | TC71440 | 14 | 14 | 8.212 | 9.118 | 0.002 | 0.611 |
| Death associated transcription factor 1 (Death | TC83440 | 14 | 14 | 6.262 | 6.482 | 0.002 | 0.611 |
| Putative eukaryotic translation initiation factor 3 | CA376626 | 14 | 14 | 5.842 | 6.156 | 0.002 | 0.611 |
| Mitochondrial 28S ribosomal protein S36 (S36mt) (MRP-S36) | TC87061 | 14 | 14 | 10.021 | 9.786 | 0.002 | 0.611 |
| unnamed protein product [Tetraodon nigroviridis] | TC77868 | 14 | 14 | 7.253 | 7.450 | 0.002 | 0.611 |

**Table S3.** *Cont.*

| **(d) Kidney: Albock deformed (Pop1) *vs.* Albock normal (Pop2)** |  |  |  |  |  |  |  |
| --- | --- | --- | --- | --- | --- | --- | --- |
| **Gene ID** | **Tigr ID** | **N_1_** | **N_2_** | **x_1_** | **x_2_** | ***P*** | **FDR** |
| Keratocan precursor (KTN) (Keratan sulfate proteoglycan | TC81914 | 14 | 14 | 6.256 | 5.739 | 0.002 | 0.611 |
| Microtubule-associated protein tau (Neurofibrillary tangle | TC72828 | 14 | 14 | 5.617 | 5.235 | 0.002 | 0.611 |
| transient receptor potential cation channel, subfamily V, member 4; | BX859296 | 14 | 14 | 6.180 | 5.983 | 0.002 | 0.611 |
| similar to RIKEN cDNA A730055C05 gene [Homo sapiens] | CR369887 | 14 | 14 | 7.224 | 7.671 | 0.002 | 0.611 |
| SI:bZ1G18.3 (novel protein similar to vertebrate gliacolin (C1Q)) | BX320366 | 14 | 14 | 5.745 | 6.069 | 0.002 | 0.611 |
| similar to RIKEN cDNA A730055C05 gene [Homo sapiens] | TC81075 | 14 | 14 | 8.903 | 9.206 | 0.002 | 0.611 |
| SH3-domain kinase binding protein 1 (Cbl-interacting | TC93012 | 14 | 14 | 7.527 | 7.765 | 0.002 | 0.611 |
| Homeobox protein Nkx-3.2 (Bagpipe homeobox protein homolog | BX300167 | 14 | 14 | 8.426 | 7.752 | 0.002 | 0.611 |
| Zinc transporter 1 (ZnT-1) | BX871381 | 14 | 14 | 12.275 | 11.848 | 0.002 | 0.611 |
| Hypoxia-inducible factor 1 alpha (HIF-1 alpha) (HIF1 | CA383850 | 14 | 14 | 5.550 | 5.794 | 0.002 | 0.611 |
| Tumor necrosis factor, alpha-induced protein 1, | CA373455 | 14 | 14 | 8.890 | 9.146 | 0.002 | 0.611 |
| myosin regulatory light chain interacting protein; wu:fj36b03 | TC77315 | 14 | 14 | 9.296 | 8.927 | 0.002 | 0.611 |
| ADP-ribosylation factor GTPase activating protein 1 | BX871419 | 14 | 14 | 6.718 | 6.868 | 0.002 | 0.611 |
| limkain beta 2 [Homo sapiens] | TC70813 | 14 | 14 | 8.118 | 7.579 | 0.002 | 0.611 |
| Brush border 61.9 kDa protein precursor | CA354102 | 14 | 14 | 8.911 | 9.557 | 0.002 | 0.611 |
| Myosin light polypeptide 6 (Myosin light chain alkali 3) | TC70741 | 14 | 14 | 11.276 | 10.597 | 0.003 | 0.618 |
| 60S ribosomal protein L9 | TC69755 | 14 | 14 | 10.268 | 10.116 | 0.003 | 0.618 |
| Polypeptide N-acetylgalactosaminyltransferase (EC | TC77029 | 14 | 14 | 6.317 | 6.061 | 0.003 | 0.620 |
| Mitogen-activated protein kinase kinase kinase 5 (EC | CX038496 | 14 | 14 | 6.686 | 6.978 | 0.003 | 0.620 |
| DNA topoisomerase II, beta isozyme (EC 5.99.1.3) | CA382478 | 14 | 14 | 6.665 | 6.948 | 0.003 | 0.620 |
| Putative serine/threonine-protein kinase C41C4.4 precursor | TC70611 | 14 | 14 | 9.248 | 9.593 | 0.003 | 0.620 |
| unnamed protein product [Tetraodon nigroviridis] | CR371201 | 14 | 14 | 5.884 | 5.545 | 0.003 | 0.620 |
| PREDICTED: similar to KIAA1530 protein, partial [Gallus gallus] | BX887054 | 14 | 14 | 7.448 | 7.671 | 0.003 | 0.620 |
| unnamed protein product [Tetraodon nigroviridis] | TC77584 | 14 | 14 | 8.326 | 8.758 | 0.003 | 0.620 |
| 3-oxoacyl-[acyl-carrier-protein] synthase II (EC 2.3.1.41) | BX084635 | 14 | 14 | 5.518 | 5.082 | 0.003 | 0.620 |
| Bromodomain adjacent to zinc finger domain protein 1A | TC77627 | 14 | 14 | 8.552 | 9.024 | 0.003 | 0.620 |
| SOUL protein [Gallus gallus] | TC73178 | 14 | 14 | 7.103 | 6.828 | 0.003 | 0.620 |
| RAD50 homolog [Mus musculus] | CA354596 | 14 | 14 | 5.972 | 6.214 | 0.003 | 0.620 |
| unnamed protein product [Tetraodon nigroviridis] | CA363824 | 14 | 14 | 6.534 | 6.825 | 0.003 | 0.620 |
| NGFI-A binding protein 2 (EGR-1 binding protein 2) | BX305577 | 14 | 14 | 6.406 | 6.170 | 0.003 | 0.620 |
| Mitogen-activated protein kinase kinase kinase kinase 2 (EC | CR363759 | 14 | 14 | 10.476 | 10.761 | 0.003 | 0.620 |
| Integrin alpha-5 precursor (Fibronectin receptor alpha | BX864621 | 14 | 14 | 7.091 | 6.763 | 0.003 | 0.620 |
| Fibroblast growth factor-12 (FGF-12) (Fibroblast growth | TC83274 | 14 | 14 | 5.540 | 5.289 | 0.003 | 0.620 |
| Chloride channel protein ClC-KB (ClC-K2) | CA379256 | 14 | 14 | 5.576 | 5.232 | 0.003 | 0.620 |
| receptor interacting protein kinase 5 [Gallus gallus] | CR374717 | 14 | 14 | 8.599 | 8.869 | 0.003 | 0.626 |
| unnamed protein product [Tetraodon nigroviridis] | CA372555 | 14 | 14 | 5.720 | 6.029 | 0.003 | 0.626 |
| Myosin heavy chain, nonmuscle type A (Cellular myosin heavy | TC76997 | 14 | 14 | 6.426 | 6.708 | 0.003 | 0.632 |
| Ran-binding protein 2 (RanBP2) (Nuclear pore complex protein | CX016051 | 14 | 14 | 5.969 | 6.159 | 0.003 | 0.632 |
| Laminin alpha-1 chain precursor (Laminin A chain) | CA348264 | 14 | 14 | 9.289 | 8.494 | 0.003 | 0.630 |
| Intersectin 1 (EH and SH3 domains protein 1) | TC93416 | 14 | 14 | 6.707 | 7.098 | 0.003 | 0.630 |
| Nuclear protein Hcc-1 | TC80245 | 14 | 14 | 11.323 | 11.512 | 0.003 | 0.630 |
| Regulator of presynaptic activity aex-3 | BX878184 | 14 | 14 | 8.464 | 8.173 | 0.004 | 0.642 |
| Mothers against decapentaplegic homolog interacting protein | TC72231 | 14 | 14 | 6.742 | 6.603 | 0.004 | 0.646 |
| PREDICTED: similar to RIKEN cDNA 4931400A14 [Gallus gallus] | CX039651 | 14 | 14 | 7.265 | 7.435 | 0.004 | 0.646 |

**Table S3.** *Cont.*

| **(d) Kidney: Albock deformed (Pop1) *vs.* Albock normal (Pop2)** |  |  |  |  |  |  |  |
| --- | --- | --- | --- | --- | --- | --- | --- |
| **Gene ID** | **Tigr ID** | **N_1_** | **N_2_** | **x_1_** | **x_2_** | ***P*** | **FDR** |
| Human enhancer of invasion 10 (EC 6.3.2.-) (E3 ubiquitin | TC81003 | 14 | 14 | 7.920 | 7.692 | 0.004 | 0.646 |
| Serine/threonine-protein kinase MAK (EC 2.7.1.37) (Male | TC91721 | 14 | 14 | 6.638 | 7.047 | 0.004 | 0.646 |
| Mannosyl-oligosaccharide 1,2-alpha-mannosidase IC (EC | CA387146 | 14 | 14 | 7.068 | 7.308 | 0.004 | 0.646 |
| gag-protease [Takifugu rubripes] | TC73773 | 14 | 14 | 5.558 | 5.328 | 0.004 | 0.646 |
| A-kinase anchor protein 7 isoform gamma (Protein kinase A | BX080699 | 14 | 14 | 5.205 | 5.508 | 0.004 | 0.646 |
| non-imprinted in Prader-Willi/Angelman syndrome 1; spastic | TC83135 | 14 | 14 | 5.837 | 6.235 | 0.004 | 0.646 |
| unnamed protein product [Tetraodon nigroviridis] | TC79670 | 14 | 14 | 6.967 | 7.243 | 0.004 | 0.646 |
| kelch-like ECH-associated protein 1 [Danio rerio] | TC84847 | 14 | 14 | 6.755 | 7.273 | 0.004 | 0.646 |
| Fatty acid-binding protein, liver (L-FABP) (Liver basic | TC69944 | 14 | 14 | 9.250 | 8.759 | 0.004 | 0.646 |
| SmcY protein (Histocompatibility Y antigen) (H-Y) | CA375178 | 14 | 14 | 7.992 | 7.776 | 0.004 | 0.646 |
| Nucleosomal binding protein 1 | CX040369 | 14 | 14 | 7.277 | 7.484 | 0.004 | 0.646 |
| Brush border 61.9 kDa protein precursor | BX088050 | 14 | 14 | 9.854 | 11.302 | 0.004 | 0.646 |
| Chemokine receptor-like 1 (G-protein coupled receptor DEZ) | CA380158 | 14 | 14 | 9.196 | 8.523 | 0.004 | 0.646 |
| Brush border 61.9 kDa protein precursor | BX088051 | 14 | 14 | 8.165 | 9.453 | 0.004 | 0.646 |
| Carbonic anhydrase XII precursor (EC 4.2.1.1) (Carbonate | TC80241 | 14 | 14 | 6.798 | 6.643 | 0.004 | 0.655 |
| Laminin alpha-3 chain precursor (Epiligrin 170 kDa subunit) | TC94653 | 14 | 14 | 4.931 | 5.550 | 0.004 | 0.665 |
| Adenosine kinase (EC 2.7.1.20) (AK) (Adenosine | CA354063 | 14 | 14 | 8.917 | 8.549 | 0.004 | 0.677 |
| Agrin (Fragment) | TC81586 | 14 | 14 | 6.401 | 7.125 | 0.005 | 0.677 |
| DNA repair protein rad3 | CR364255 | 14 | 14 | 7.320 | 7.671 | 0.005 | 0.677 |
| Serine protease HTRA1 precursor (EC 3.4.21.-) (L56) | TC71882 | 14 | 14 | 7.893 | 7.062 | 0.005 | 0.677 |
| Syntaxin 7 | TC71364 | 14 | 14 | 8.866 | 9.154 | 0.005 | 0.677 |
| unnamed protein product [Tetraodon nigroviridis] | TC72061 | 14 | 14 | 6.401 | 6.690 | 0.005 | 0.677 |
| Galectin-3 (Galactose-specific lectin 3) (MAC-2 antigen) | TC87132 | 14 | 14 | 9.501 | 9.243 | 0.005 | 0.677 |
| Mitochondrial ribosomal protein L38 [Danio rerio] | TC74161 | 14 | 14 | 5.614 | 5.426 | 0.005 | 0.677 |
| RIKEN cDNA 9930116O05 gene [Mus musculus] | TC92231 | 14 | 14 | 6.698 | 6.328 | 0.005 | 0.677 |
| Brush border 61.9 kDa protein precursor | CA378216 | 14 | 14 | 8.354 | 9.314 | 0.005 | 0.677 |
| Dynein gamma chain, flagellar outer arm | BX858167 | 14 | 14 | 7.981 | 7.720 | 0.005 | 0.677 |
| hypothetical protein [Gallus gallus] | TC87545 | 14 | 14 | 8.113 | 8.515 | 0.005 | 0.677 |
| unnamed protein product [Tetraodon nigroviridis] | TC90980 | 14 | 14 | 5.773 | 5.645 | 0.005 | 0.677 |
| Proteasome subunit beta type 7 precursor (EC 3.4.25.1) | TC70076 | 14 | 14 | 13.116 | 12.671 | 0.005 | 0.677 |
| SWAP-70 protein [Homo sapiens] | TC73042 | 14 | 14 | 8.613 | 8.981 | 0.005 | 0.677 |
| Ankyrin repeat domain protein 28 (Fragment) | TC80349 | 14 | 14 | 7.413 | 7.663 | 0.005 | 0.677 |
| unnamed protein product [Tetraodon nigroviridis] | TC70068 | 14 | 14 | 7.449 | 7.172 | 0.005 | 0.677 |
| Epidermal growth factor receptor kinase substrate EPS8 | CA369207 | 14 | 14 | 6.072 | 5.918 | 0.005 | 0.677 |
| Spectrin beta chain, brain 1 (Spectrin, non-erythroid beta | TC72830 | 14 | 14 | 13.732 | 14.101 | 0.005 | 0.677 |
| Adenylate cyclase, type VII (EC 4.6.1.1) (ATP | TC83609 | 14 | 14 | 8.831 | 9.094 | 0.005 | 0.677 |
| DNA replication licensing factor MCM4 (CDC21 homolog) | TC79253 | 14 | 14 | 5.808 | 5.471 | 0.005 | 0.677 |
| Diacylglycerol kinase, delta (EC 2.7.1.107) (Diglyceride | CA351948 | 14 | 14 | 5.297 | 5.509 | 0.005 | 0.677 |
| DNA-binding protein Ikaros | U92201 | 14 | 14 | 13.204 | 13.426 | 0.005 | 0.677 |
| Alpha-methylacyl-CoA racemase (EC 5.1.99.4) | TC73745 | 14 | 14 | 10.455 | 9.744 | 0.005 | 0.677 |
| Mitogen-activated protein kinase kinase kinase 14 (EC | CA371282 | 14 | 14 | 8.683 | 8.987 | 0.006 | 0.677 |
| hypothetical protein MGC55407 [Danio rerio] | CA360110 | 14 | 14 | 6.451 | 6.684 | 0.006 | 0.677 |
| Brush border 61.9 kDa protein precursor | BX875164 | 14 | 14 | 9.186 | 10.601 | 0.006 | 0.677 |
| Serine/threonine-protein kinase Sgk2 (EC 2.7.1.37) | BX864334 | 14 | 14 | 8.005 | 7.465 | 0.006 | 0.677 |
| Friend leukemia integration 1 transcription factor | CA341888 | 14 | 14 | 8.479 | 8.634 | 0.006 | 0.677 |

**Table S3.** *Cont.*

| **(d) Kidney: Albock deformed (Pop1) *vs.* Albock normal (Pop2)** |  |  |  |  |  |  |  |
| --- | --- | --- | --- | --- | --- | --- | --- |
| **Gene ID** | **Tigr ID** | **N_1_** | **N_2_** | **x_1_** | **x_2_** | ***P*** | **FDR** |
| unnamed protein product [Tetraodon nigroviridis] | TC79465 | 14 | 14 | 6.203 | 6.052 | 0.006 | 0.677 |
| Synaptophysin (Major synaptic vesicle protein p38) | TC74949 | 14 | 14 | 5.856 | 6.264 | 0.006 | 0.677 |
| NACHT-, LRR- and PYD-containing protein 12 (PYRIN-containing | BX858824 | 14 | 14 | 12.921 | 13.557 | 0.006 | 0.677 |
| Zona pellucida sperm-binding protein 4 precursor (Zona | TC80099 | 14 | 14 | 6.923 | 6.616 | 0.006 | 0.677 |
| Forkhead box protein P1 (Forkhead-related transcription | TC93537 | 14 | 14 | 7.328 | 7.061 | 0.006 | 0.677 |
| DNA nucleotidylexotransferase (Terminal addition enzyme) (Terminal | U53366 | 14 | 14 | 6.180 | 5.984 | 0.006 | 0.677 |
| CBL E3 ubiquitin protein ligase (EC 6.3.2.-) (Signal | TC83768 | 14 | 14 | 7.897 | 8.114 | 0.006 | 0.677 |
| EMILIN 2 precursor (Elastin microfibril interface-located | TC81654 | 14 | 14 | 8.765 | 8.450 | 0.006 | 0.677 |
| unnamed protein product [Tetraodon nigroviridis] | TC93236 | 14 | 14 | 7.374 | 7.115 | 0.006 | 0.677 |
| Anthrax toxin receptor 1 precursor (Tumor endothelial | BX866999 | 14 | 14 | 8.320 | 8.656 | 0.006 | 0.677 |
| PREDICTED: similar to HECT domain containing 2 [Gallus gallus] | BX869565 | 14 | 14 | 8.777 | 8.366 | 0.006 | 0.677 |
| Zinc finger protein 143 (SPH-binding factor) | CX031984 | 14 | 14 | 7.498 | 7.246 | 0.006 | 0.677 |
| Brush border 61.9 kDa protein precursor | BX860397 | 14 | 14 | 6.841 | 8.220 | 0.006 | 0.677 |
| wu:fb11h03; lipocalin-type prostaglandin D synthase-like; | CA358499 | 14 | 14 | 8.309 | 7.710 | 0.006 | 0.677 |
| CUG triplet repeat, RNA binding protein 2; elav-type RNA-binding | CA343771 | 14 | 14 | 5.804 | 6.107 | 0.006 | 0.677 |
| DNA replication licensing factor MCM8 (Minichromosome | TC91229 | 14 | 14 | 9.305 | 9.504 | 0.006 | 0.677 |
| Dedicator of cytokinesis protein 2 (Hch protein) | CA386553 | 14 | 14 | 9.755 | 10.085 | 0.006 | 0.677 |
| Polyhomeotic-like protein 1 (Early development regulator | TC87270 | 14 | 14 | 10.675 | 10.961 | 0.007 | 0.677 |
| MYST histone acetyltransferase 2 [Danio rerio] | CA385023 | 14 | 14 | 8.896 | 9.094 | 0.007 | 0.677 |
| Polymyositis/scleroderma autoantigen 2 (Autoantigen PM/Scl | CA360761 | 14 | 14 | 7.469 | 6.765 | 0.007 | 0.677 |
| Forkhead box protein P4 (Fork head-related protein like A) | BX867531 | 14 | 14 | 6.741 | 6.922 | 0.007 | 0.677 |
| parapinopsin [Oncorhynchus mykiss] | AB159673 | 14 | 14 | 7.311 | 7.041 | 0.007 | 0.677 |
| Loss of heterozygosity 11 chromosomal region 2 gene A | TC72589 | 14 | 14 | 9.124 | 9.478 | 0.007 | 0.677 |
| Vacuolar protein sorting 35 (Vesicle protein sorting 35) | CA366526 | 14 | 14 | 8.985 | 9.256 | 0.007 | 0.677 |
| PREDICTED: similar to hypothetical protein [Gallus gallus] | CA347479 | 14 | 14 | 5.709 | 5.981 | 0.007 | 0.677 |
| Forkhead box protein F2 (Forkhead-related protein FKHL6) | TC77941 | 14 | 14 | 5.377 | 5.176 | 0.007 | 0.677 |
| Zinc finger protein 16 (Zinc finger protein KOX9) | TC71695 | 14 | 14 | 11.849 | 12.016 | 0.007 | 0.677 |
| Epidermal growth factor receptor kinase substrate EPS8 | CA361524 | 14 | 14 | 7.541 | 7.282 | 0.007 | 0.677 |
| unnamed protein product [Tetraodon nigroviridis] | CX037322 | 14 | 14 | 9.808 | 9.269 | 0.007 | 0.677 |
| Gamma crystallin M3 (Gamma-M3) | BX887500 | 14 | 14 | 7.089 | 6.838 | 0.007 | 0.677 |
| Mucosa associated lymphoid tissue lymphoma translocation | CA381994 | 14 | 14 | 6.146 | 6.569 | 0.007 | 0.677 |
| Ictacalcin | BX914141 | 14 | 14 | 12.539 | 11.630 | 0.007 | 0.677 |
| Aldose reductase (EC 1.1.1.21) (AR) (Aldehyde reductase) | TC70267 | 14 | 14 | 9.704 | 9.430 | 0.007 | 0.677 |
| zinc finger protein 622; zinc finger-like protein 9 [Homo sapiens] | TC91180 | 14 | 14 | 9.414 | 9.228 | 0.007 | 0.677 |
| PREDICTED: similar to SF21 protein [Gallus gallus] | CA361579 | 14 | 14 | 9.245 | 9.422 | 0.007 | 0.677 |
| Transcription initiation factor TFIID subunit 1 | TC91855 | 14 | 14 | 10.540 | 10.769 | 0.007 | 0.677 |
| Peroxisomal acyl-coenzyme A thioester hydrolase 2a (EC | CA374528 | 14 | 14 | 6.126 | 6.396 | 0.007 | 0.677 |
| unnamed protein product [Tetraodon nigroviridis] | CA384997 | 14 | 14 | 5.400 | 5.571 | 0.007 | 0.677 |
| unnamed protein product [Tetraodon nigroviridis] | CA344009 | 14 | 14 | 6.345 | 6.188 | 0.007 | 0.677 |
| S100 calcium-binding protein A5 (S-100D protein) | TC73707 | 14 | 14 | 9.164 | 8.280 | 0.007 | 0.677 |
| Hermansky-Pudlak syndrome 3 protein | TC81739 | 14 | 14 | 9.329 | 9.692 | 0.007 | 0.677 |
| Cytochrome c oxidase assembly protein COX11, mitochondrial | TC89224 | 14 | 14 | 8.959 | 8.779 | 0.007 | 0.677 |
| SWAP-70 protein [Homo sapiens] | TC74224 | 14 | 14 | 10.316 | 10.760 | 0.007 | 0.677 |

**Table S3.** *Cont.*

| **(d) Kidney: Albock deformed (Pop1) *vs.* Albock normal (Pop2)** |  |  |  |  |  |  |  |
| --- | --- | --- | --- | --- | --- | --- | --- |
| **Gene ID** | **Tigr ID** | **N_1_** | **N_2_** | **x_1_** | **x_2_** | ***P*** | **FDR** |
| Zinc-finger protein HT2A (72 kDa Tat-interacting protein) | TC75444 | 14 | 14 | 7.278 | 7.120 | 0.007 | 0.677 |
| sperm associated antigen 6 [Mus musculus] | TC76845 | 14 | 14 | 7.151 | 6.956 | 0.008 | 0.677 |
| unnamed protein product [Tetraodon nigroviridis] | BX872356 | 14 | 14 | 6.366 | 6.093 | 0.008 | 0.677 |
| NACHT-, LRR- and PYD-containing protein 12 (PYRIN-containing | BX319693 | 14 | 14 | 7.381 | 7.832 | 0.008 | 0.677 |
| Beta-centractin (Actin-related protein 1B) (ARP1B) | TC78630 | 14 | 14 | 11.074 | 10.852 | 0.008 | 0.677 |
| ATP-binding cassette, sub-family A, member 1 (ATP-binding | CA363869 | 14 | 14 | 8.194 | 7.889 | 0.008 | 0.677 |
| Homeobox protein Hox-C8 (Hox-3.1) (M31) | TC83987 | 14 | 14 | 6.194 | 5.997 | 0.008 | 0.677 |
| Zinc finger protein 16 (Zinc finger protein KOX9) | BX889488 | 14 | 14 | 8.458 | 8.698 | 0.008 | 0.677 |
| Probable chromodomain-helicase-DNA-binding protein KIAA1416 | BX880179 | 14 | 14 | 8.743 | 9.003 | 0.008 | 0.677 |
| F-box/LRR-repeat protein 11 (F-box and leucine-rich repeat | TC73533 | 14 | 14 | 6.286 | 6.537 | 0.008 | 0.677 |
| Fatty acid-binding protein, liver (L-FABP) (Liver basic | CA385329 | 14 | 14 | 10.190 | 9.665 | 0.008 | 0.677 |
| PREDICTED: similar to 4930544G21Rik protein [Gallus gallus] | CA381387 | 14 | 14 | 6.827 | 6.533 | 0.008 | 0.677 |
| zgc:92858 [Danio rerio] | TC90310 | 14 | 14 | 8.072 | 8.423 | 0.008 | 0.677 |
| NADH-ubiquinone oxidoreductase 18 kDa subunit, | TC70031 | 14 | 14 | 13.399 | 13.243 | 0.008 | 0.677 |
| B-cell antigen receptor complex associated protein | TC73657 | 14 | 14 | 6.646 | 7.021 | 0.008 | 0.677 |
| translin-associated factor X interacting protein 1 [Homo sapiens] | TC79796 | 14 | 14 | 6.266 | 6.597 | 0.008 | 0.677 |
| SH2 domain protein 2A (T cell-specific adapter protein) | CA372550 | 14 | 14 | 6.781 | 7.023 | 0.008 | 0.677 |
| dihydrodiol dehydrogenase (dimeric), like [Danio rerio] | CA382347 | 14 | 14 | 7.367 | 7.096 | 0.008 | 0.677 |
| NAD(P)H dehydrogenase [quinone] 1 (EC 1.6.99.2) (Quinone | TC70300 | 14 | 14 | 10.534 | 10.887 | 0.008 | 0.677 |
| DNA repair protein rad3 | TC88945 | 14 | 14 | 10.069 | 10.289 | 0.008 | 0.677 |
| Cohesin subunit SA-1 (XSA-1) (Stromal antigen 1 homolog) | BX860761 | 14 | 14 | 7.004 | 7.249 | 0.008 | 0.677 |
| DNA (cytosine-5)-methyltransferase 3B (EC 2.1.1.37) | CA357958 | 14 | 14 | 6.445 | 6.772 | 0.008 | 0.677 |
| Splicing factor, arginine/serine-rich 5 (Pre-mRNA splicing | TC69579 | 14 | 14 | 9.131 | 9.620 | 0.008 | 0.677 |
| unnamed protein product [Tetraodon nigroviridis] | CA372956 | 14 | 14 | 7.432 | 7.637 | 0.008 | 0.677 |
| Alpha-1,3-mannosyl-glycoprotein | CA364012 | 14 | 14 | 6.367 | 6.225 | 0.008 | 0.677 |
| HORMA domain containing protein [Homo sapiens] | TC72240 | 14 | 14 | 8.316 | 8.012 | 0.008 | 0.677 |
| Cytochrome b | TC69298 | 14 | 14 | 14.075 | 14.370 | 0.008 | 0.677 |
| zgc:56115 [Danio rerio] | TC74225 | 14 | 14 | 7.412 | 8.121 | 0.009 | 0.677 |
| PREDICTED: similar to cAMP responsive element binding protein 3-like | TC82428 | 14 | 14 | 6.589 | 6.271 | 0.009 | 0.677 |
| F-box/LRR-repeat protein 9 (F-box and leucine-rich repeat | CR373251 | 14 | 14 | 8.496 | 8.287 | 0.009 | 0.677 |
| Phosphatidylinositol 3-kinase regulatory alpha subunit | BX300004 | 14 | 14 | 6.679 | 6.403 | 0.009 | 0.677 |
| T cell receptor gamma chain V-J-C1 [Paralichthys olivaceus] | TC74323 | 14 | 14 | 7.875 | 8.351 | 0.009 | 0.677 |
| Sodium/glucose cotransporter 2 (Na(+)/glucose cotransporter | TC81751 | 14 | 14 | 6.409 | 6.213 | 0.009 | 0.677 |
| hypothetical protein zgc:85938 [Danio rerio] | TC86695 | 14 | 14 | 10.545 | 9.806 | 0.009 | 0.677 |
| unnamed protein product [Tetraodon nigroviridis] | TC84301 | 14 | 14 | 6.167 | 7.814 | 0.009 | 0.677 |
| Alkylated DNA repair protein alkB homolog | BX082990 | 14 | 14 | 7.090 | 6.756 | 0.009 | 0.677 |
| TPA: transposase [Rana pipiens] | CA367179 | 14 | 14 | 13.643 | 13.819 | 0.009 | 0.677 |
| ubiquitin associated protein 2 isoform 1; AD-012 protein [Homo | TC83668 | 14 | 14 | 7.930 | 8.161 | 0.009 | 0.677 |
| Myelin and lymphocyte protein (T-lymphocyte | TC71701 | 14 | 14 | 5.900 | 5.288 | 0.009 | 0.677 |
| PREDICTED: similar to RIKEN cDNA 1110001J12 [Gallus gallus] | TC93033 | 14 | 14 | 5.724 | 5.852 | 0.009 | 0.677 |
| Cytochrome c-type heme lyase (EC 4.4.1.17) (CCHL) | TC71632 | 14 | 14 | 10.698 | 11.012 | 0.009 | 0.677 |
| Myoferlin (Fer-1 like protein 3) | TC77766 | 14 | 14 | 6.464 | 6.070 | 0.009 | 0.677 |
| Golgi apparatus protein 1 precursor (Cysteine-rich | TC78640 | 14 | 14 | 7.840 | 8.372 | 0.009 | 0.677 |

**Table S3.** *Cont.*

| **(d) Kidney: Albock deformed (Pop1) *vs.* Albock normal (Pop2)** |  |  |  |  |  |  |  |
| --- | --- | --- | --- | --- | --- | --- | --- |
| **Gene ID** | **Tigr ID** | **N_1_** | **N_2_** | **x_1_** | **x_2_** | ***P*** | **FDR** |
| Heterogeneous nuclear ribonucleoprotein A0 (hnRNP A0) | TC86571 | 14 | 14 | 11.592 | 11.895 | 0.009 | 0.677 |
| Adenosine A2a receptor | BX911303 | 14 | 14 | 6.910 | 7.144 | 0.009 | 0.677 |
| PREDICTED: similar to DEP domain containing protein 5 [Gallus gallus] | TC92200 | 14 | 14 | 7.589 | 7.839 | 0.009 | 0.677 |
| Organic cation/carnitine transporter 2 (Solute carrier | TC84695 | 14 | 14 | 8.135 | 7.897 | 0.009 | 0.677 |
| Cocaine- and amphetamine-regulated transcript protein | TC77298 | 14 | 14 | 8.154 | 7.757 | 0.009 | 0.677 |
| Integrin beta-1* precursor | CA382877 | 14 | 14 | 9.281 | 9.630 | 0.009 | 0.677 |
| PREDICTED: similar to ring finger protein 123 [Gallus gallus] | CA347442 | 14 | 14 | 9.102 | 8.884 | 0.009 | 0.677 |
| Zinc finger protein 462 | TC90364 | 14 | 14 | 6.770 | 7.070 | 0.009 | 0.677 |
| APG7 autophagy 7-like; ubiquitin activating enzyme E1-like protein | TC85275 | 14 | 14 | 6.717 | 6.481 | 0.009 | 0.677 |
| PREDICTED: similar to ubiquitin specific protease 47 [Gallus gallus] | TC78647 | 14 | 14 | 14.360 | 13.882 | 0.009 | 0.677 |
| pleckstrin homology domain containing, family M (with RUN domain) | TC89869 | 14 | 14 | 7.531 | 7.865 | 0.009 | 0.677 |
| PREDICTED: similar to ribosome associated membrane protein 4 [Homo | TC85456 | 14 | 14 | 7.355 | 7.192 | 0.009 | 0.677 |
| unnamed protein product [Tetraodon nigroviridis] | BX859981 | 14 | 14 | 6.931 | 7.187 | 0.009 | 0.677 |
| RAS guanyl releasing protein 3 (Calcium and DAG-regulated | TC75017 | 14 | 14 | 7.253 | 7.565 | 0.009 | 0.677 |
| SH2 domain protein 2A (T cell-specific adapter protein) | CA378519 | 14 | 14 | 7.806 | 8.236 | 0.009 | 0.677 |
| unnamed protein product [Tetraodon nigroviridis] | TC91057 | 14 | 14 | 9.416 | 9.182 | 0.010 | 0.677 |
| Keratin associated protein 4-5 (Keratin associated protein | BX885050 | 14 | 14 | 15.529 | 15.706 | 0.010 | 0.677 |
| ARF GTPase-activating protein GIT2 (G protein-coupled | TC85986 | 14 | 14 | 6.643 | 6.851 | 0.010 | 0.677 |
| Von Hippel-Lindau disease tumor suppressor (pVHL) | TC85757 | 14 | 14 | 6.990 | 6.737 | 0.010 | 0.677 |
| Heat shock 27 kDa protein (HSP 27) (Stress-responsive | TC72081 | 14 | 14 | 6.351 | 6.140 | 0.010 | 0.677 |
| unnamed protein product [Tetraodon nigroviridis] | BX304335 | 14 | 14 | 9.925 | 9.674 | 0.010 | 0.677 |
| Nop14-like; ik:tdsubc_1a2; xx:tdsubc_1a2 [Danio rerio] | TC71767 | 14 | 14 | 12.462 | 12.054 | 0.010 | 0.677 |
| Zinc finger protein ZFMSA12A | TC71304 | 14 | 14 | 8.735 | 8.908 | 0.010 | 0.677 |
| Elongation factor 2 (EF-2) | TC86598 | 14 | 14 | 14.366 | 14.843 | 0.010 | 0.677 |
| Malate dehydrogenase, cytoplasmic (EC 1.1.1.37) | TC69925 | 14 | 14 | 5.366 | 5.765 | 0.010 | 0.677 |
| NADP-dependent malic enzyme, mitochondrial precursor (EC | CA360677 | 14 | 14 | 6.822 | 6.624 | 0.010 | 0.679 |
| Integrin beta pat-3 precursor | CA369883 | 14 | 14 | 8.445 | 7.517 | 0.010 | 0.679 |
| Nucleolar RNA helicase II (Nucleolar RNA helicase Gu) (RH | TC78390 | 14 | 14 | 12.256 | 12.594 | 0.010 | 0.675 |
| hypothetical protein C330043M08 [Mus musculus] | TC93228 | 14 | 14 | 6.871 | 6.661 | 0.010 | 0.675 |
| putative endoplasmic reticulum multispan transmembrane protein | CA378447 | 14 | 14 | 7.962 | 7.757 | 0.010 | 0.675 |
| membrane-Toll-like receptor 5 [Oncorhynchus mykiss] | AB091105 | 14 | 14 | 5.562 | 5.772 | 0.010 | 0.675 |
|  |  |  |  |  |  |  |  |
| **(e) Kidney: Brienzlig deformed (Pop1) *vs.* Brienzlig normal (Pop2)** |  |  |  |  |  |  |  |
| **Gene ID** | **Tigr ID** | **N_1_** | **N_2_** | **x_1_** | **x_2_** | ***P*** | **FDR** |
| Syntaxin 17 | BX857368 | 14 | 14 | 5.897 | 5.599 | 0.000 | 0.314 |
| GPI-anchored protein p137 (p137GPI) | CA386677 | 14 | 14 | 6.654 | 6.987 | 0.000 | 0.314 |
| MYC I protein (C-MYC I) | TC90805 | 14 | 14 | 11.463 | 11.049 | 0.000 | 0.314 |
| 3-mercaptopyruvate sulfurtransferase (EC 2.8.1.2) (MST) | TC86833 | 14 | 14 | 9.256 | 8.623 | 0.000 | 0.314 |
| Syntaxin 17 | BX857368 | 14 | 14 | 5.897 | 5.599 | 0.000 | 0.314 |

**Table S3.** *Cont.*

| **(e) Kidney: Brienzlig deformed (Pop1) *vs.* Brienzlig normal (Pop2)** |  |  |  |  |  |  |  |
| --- | --- | --- | --- | --- | --- | --- | --- |
| **Gene ID** | **Tigr ID** | **N_1_** | **N_2_** | **x_1_** | **x_2_** | ***P*** | **FDR** |
| GPI-anchored protein p137 (p137GPI) | CA386677 | 14 | 14 | 6.654 | 6.987 | 0.000 | 0.314 |
| MYC I protein (C-MYC I) | TC90805 | 14 | 14 | 11.463 | 11.049 | 0.000 | 0.314 |
| 3-mercaptopyruvate sulfurtransferase (EC 2.8.1.2) (MST) | TC86833 | 14 | 14 | 9.256 | 8.623 | 0.000 | 0.314 |
| Replication protein A 14 kDa subunit (RP-A) (RF-A) | TC78866 | 14 | 14 | 11.445 | 12.040 | 0.000 | 0.314 |
| Nedd-4-like ubiquitin-protein ligase WWP2 (EC 6.3.2.-) (WW | TC93189 | 14 | 14 | 5.509 | 5.712 | 0.000 | 0.314 |
| NAD-dependent deacetylase sirtuin 6 (EC 3.5.1.-) | CX038407 | 14 | 14 | 6.804 | 7.110 | 0.000 | 0.314 |
| Transposable element TCB2 transposase | CA353291 | 14 | 14 | 13.446 | 13.786 | 0.000 | 0.314 |
| unnamed protein product [Tetraodon nigroviridis] | BX864543 | 14 | 14 | 5.511 | 5.907 | 0.000 | 0.314 |
| N-sulphoglucosamine sulphohydrolase precursor (EC 3.10.1.1) | TC91134 | 14 | 14 | 6.629 | 5.909 | 0.001 | 0.314 |
| Dedicator of cytokinesis protein 10 (Protein zizimin 3) | CF753039 | 14 | 14 | 6.140 | 6.318 | 0.001 | 0.314 |
| unnamed protein product [Tetraodon nigroviridis] | TC86101 | 14 | 14 | 7.726 | 7.264 | 0.001 | 0.314 |
| Transmembrane protein PFT27 (TPA regulated locus protein) | BX311803 | 14 | 14 | 6.822 | 6.330 | 0.001 | 0.314 |
| Phosphoglucomutase (EC 5.4.2.2) (Glucose phosphomutase) | BX310729 | 14 | 14 | 6.815 | 7.496 | 0.001 | 0.314 |
| PREDICTED: similar to TRAF2 binding protein; TRAF6 binding protein | TC81612 | 14 | 14 | 6.439 | 6.991 | 0.001 | 0.314 |
| Protein-tyrosine phosphatase, non-receptor type 9 (EC | BX911086 | 14 | 14 | 6.070 | 5.784 | 0.001 | 0.314 |
| Complement C3-1 [Contains: C3a anaphylatoxin] (Fragment) | TC78118 | 14 | 14 | 5.364 | 5.800 | 0.001 | 0.314 |
| Microfibril-associated glycoprotein 4 precursor | TC88635 | 14 | 14 | 7.172 | 5.780 | 0.001 | 0.314 |
| NOD9 protein isoform 1 [Homo sapiens] | BX074699 | 14 | 14 | 7.620 | 7.076 | 0.001 | 0.314 |
| Voltage-gated potassium channel beta-2 subunit (K+ channel | BX863792 | 14 | 14 | 8.096 | 8.547 | 0.001 | 0.314 |
| Alpha-1 catenin (102 kDa cadherin-associated protein) | TC88051 | 14 | 14 | 9.111 | 9.474 | 0.001 | 0.314 |
| Neuroendocrine convertase 2 precursor (EC 3.4.21.94) (NEC | TC90286 | 14 | 14 | 5.865 | 6.373 | 0.001 | 0.314 |
| Neuronal cell death inducible putative kinase (SKIP3) | BX866316 | 14 | 14 | 8.476 | 7.746 | 0.001 | 0.314 |
| hypothetical protein LOC222967 [Homo sapiens] | TC82837 | 14 | 14 | 6.756 | 7.290 | 0.001 | 0.314 |
| Glycogen synthase kinase-3 alpha (EC 2.7.1.37) (GSK-3 | TC92718 | 14 | 14 | 7.731 | 7.342 | 0.001 | 0.314 |
| unnamed protein product [Tetraodon nigroviridis] | TC80726 | 14 | 14 | 11.279 | 11.065 | 0.001 | 0.314 |
| PREDICTED: similar to FLJ45273 protein [Gallus gallus] | CA380317 | 14 | 14 | 8.079 | 7.412 | 0.001 | 0.314 |
| Potential phospholipid-transporting ATPase IH (EC 3.6.3.1) | TC82882 | 14 | 14 | 5.661 | 5.945 | 0.001 | 0.314 |
| similar to hypothetical protein [Rattus norvegicus] | BX878879 | 14 | 14 | 6.850 | 6.269 | 0.001 | 0.314 |
| Liprin-beta 2 (Protein tyrosine phosphatase receptor type f | CX036505 | 14 | 14 | 15.005 | 15.184 | 0.001 | 0.314 |
| unnamed protein product [Tetraodon nigroviridis] | BX876787 | 14 | 14 | 5.965 | 6.230 | 0.001 | 0.314 |
| hypothetical protein MGC65939 [Danio rerio] | TC92933 | 14 | 14 | 8.016 | 7.467 | 0.001 | 0.314 |
| mitochondrial carrier homolog 1; presenilin-associated protein; | TC92699 | 14 | 14 | 5.849 | 6.436 | 0.001 | 0.314 |
| Hypoxia-inducible factor 1 alpha (HIF-1 alpha) (HIF1 | CA383850 | 14 | 14 | 5.715 | 5.511 | 0.001 | 0.314 |
| Cysteine string protein (CSP) (Xcsp) | CR371183 | 14 | 14 | 14.279 | 14.822 | 0.001 | 0.314 |
| Regulator of G-protein signaling 1 (RGS1) (Early response | TC92554 | 14 | 14 | 11.744 | 11.117 | 0.001 | 0.314 |
| Neuronal membrane glycoprotein M6-b (M6b) | TC72651 | 14 | 14 | 7.505 | 7.851 | 0.001 | 0.314 |
| 60S ribosomal protein L36 | TC86803 | 14 | 14 | 12.330 | 11.851 | 0.001 | 0.314 |
| Chemokine receptor-like 1 (G-protein coupled receptor DEZ) | TC73381 | 14 | 14 | 7.297 | 6.346 | 0.001 | 0.314 |
| unnamed protein product [Tetraodon nigroviridis] | TC93109 | 14 | 14 | 6.494 | 6.856 | 0.001 | 0.314 |
| SWS1 opsin [Oncorhynchus mykiss] | AF425074 | 14 | 14 | 6.574 | 7.088 | 0.001 | 0.314 |
| unnamed protein product [Tetraodon nigroviridis] | CA360257 | 14 | 14 | 5.796 | 6.112 | 0.001 | 0.314 |
| Inward rectifier potassium channel 2 (Potassium channel, | BX305560 | 14 | 14 | 5.386 | 5.620 | 0.001 | 0.314 |

**Table S3.** *Cont.*

| **(e) Kidney: Brienzlig deformed (Pop1) *vs.* Brienzlig normal (Pop2)** |  |  |  |  |  |  |  |
| --- | --- | --- | --- | --- | --- | --- | --- |
| **Gene ID** | **Tigr ID** | **N_1_** | **N_2_** | **x_1_** | **x_2_** | ***P*** | **FDR** |
| PREDICTED: similar to M-phase phosphoprotein 9 [Gallus gallus] | TC90056 | 14 | 14 | 7.533 | 7.842 | 0.001 | 0.314 |
| Thyrotropin-releasing hormone degrading ectoenzyme (EC | CA384986 | 14 | 14 | 6.086 | 6.437 | 0.001 | 0.314 |
| DnaJ homolog subfamily B member 6 (Heat shock protein J2) | TC73768 | 14 | 14 | 11.001 | 10.705 | 0.001 | 0.314 |
| Kynureninase (EC 3.7.1.3) (L-kynurenine hydrolase) | TC88574 | 14 | 14 | 9.003 | 9.451 | 0.002 | 0.314 |
| Probable serine/threonine-protein kinase pkwA (EC | TC89136 | 14 | 14 | 10.420 | 9.263 | 0.002 | 0.314 |
| unnamed protein product [Tetraodon nigroviridis] | BX912504 | 14 | 14 | 7.077 | 6.571 | 0.002 | 0.314 |
| Junctional adhesion molecule 1 precursor (JAM) | CX027759 | 14 | 14 | 5.929 | 6.230 | 0.002 | 0.314 |
| unnamed protein product [Tetraodon nigroviridis] | BX868149 | 14 | 14 | 10.278 | 9.706 | 0.002 | 0.314 |
| Protein-tyrosine phosphatase delta precursor (EC 3.1.3.48) | CA385813 | 14 | 14 | 7.256 | 7.793 | 0.002 | 0.314 |
| PREDICTED: similar to RIKEN cDNA 1700028P05 [Gallus gallus] | CA371350 | 14 | 14 | 8.400 | 9.044 | 0.002 | 0.314 |
| H4 protein | TC86036 | 14 | 14 | 10.918 | 10.704 | 0.002 | 0.314 |
| RING finger protein 23 (Testis-abundant finger protein) | BX861536 | 14 | 14 | 7.014 | 7.356 | 0.002 | 0.314 |
| High-affinity cAMP-specific 3',5'-cyclic phosphodiesterase | CX038869 | 14 | 14 | 6.334 | 6.031 | 0.002 | 0.314 |
| CCAAT/enhancer binding protein beta [Oncorhynchus mykiss] | AY144611 | 14 | 14 | 12.105 | 11.364 | 0.002 | 0.314 |
| unnamed protein product [Tetraodon nigroviridis] | TC90917 | 14 | 14 | 8.158 | 8.790 | 0.002 | 0.314 |
| mesoderm specific transcript [Takifugu rubripes] | TC76913 | 14 | 14 | 5.554 | 5.825 | 0.002 | 0.314 |
| zgc:77306 [Danio rerio] | BX081702 | 14 | 14 | 8.779 | 8.474 | 0.002 | 0.314 |
| Probable aminopeptidase NPEPL1 (EC 3.4.11.-) | TC71183 | 14 | 14 | 12.825 | 13.186 | 0.002 | 0.314 |
| Transmembrane 4 superfamily, member 8 (Tetraspanin 5) | TC80959 | 14 | 14 | 5.915 | 5.648 | 0.002 | 0.314 |
| zgc:77778 [Danio rerio] | CA357517 | 14 | 14 | 12.996 | 13.293 | 0.002 | 0.314 |
| Glutathione transferase omega 1 (EC 2.5.1.18) (GSTO 1-1) | TC78387 | 14 | 14 | 9.910 | 10.473 | 0.002 | 0.314 |
| Type I inositol-1,4,5-trisphosphate 5-phosphatase (EC | CA351545 | 14 | 14 | 5.538 | 5.257 | 0.002 | 0.314 |
| Cytochrome P450 3A27 (EC 1.14.14.1) (CYPIIIA27) | CA361991 | 14 | 14 | 7.988 | 8.449 | 0.002 | 0.314 |
| Peroxisome proliferator activated receptor gamma | CA345564 | 14 | 14 | 7.290 | 6.713 | 0.002 | 0.314 |
| Importin alpha-6 subunit (Karyopherin alpha-5 subunit) | CA344231 | 14 | 14 | 5.279 | 4.993 | 0.002 | 0.314 |
| Laminin beta-2 chain precursor (S-laminin) (S-LAM) | TC74114 | 14 | 14 | 5.984 | 6.226 | 0.002 | 0.314 |
| P300/CBP-associated factor (EC 2.3.1.-) (P/CAF) (Histone | TC84610 | 14 | 14 | 7.070 | 7.532 | 0.002 | 0.314 |
| Latent transforming growth factor beta binding protein 3 | CA351795 | 14 | 14 | 9.694 | 10.203 | 0.002 | 0.314 |
| X box binding protein-1 (XBP-1) (TREB5 protein) | TC79380 | 14 | 14 | 11.372 | 10.610 | 0.002 | 0.314 |
| Beta-1,4-galactosyltransferase 6 (EC 2.4.1.-) | TC74491 | 14 | 14 | 5.869 | 5.490 | 0.002 | 0.314 |
| helical cytokine receptor CRFB7 [Tetraodon nigroviridis] | CA388246 | 14 | 14 | 5.593 | 5.301 | 0.002 | 0.314 |
| Histone-lysine N-methyltransferase, H3 lysine-9 specific 3 | TC88414 | 14 | 14 | 8.588 | 9.034 | 0.002 | 0.314 |
| Induced myeloid leukemia cell differentiation protein | TC87110 | 14 | 14 | 15.102 | 14.610 | 0.002 | 0.314 |
| unnamed protein product [Tetraodon nigroviridis] | CA356294 | 14 | 14 | 6.230 | 6.630 | 0.002 | 0.314 |
| C-ETS-2 protein | TC94699 | 14 | 14 | 10.541 | 9.570 | 0.002 | 0.314 |
| VIP36-like protein precursor (Lectin, mannose-binding | BX862025 | 14 | 14 | 6.814 | 7.234 | 0.002 | 0.314 |
| ring finger protein 146 [Danio rerio] | TC79921 | 14 | 14 | 10.119 | 9.685 | 0.002 | 0.314 |
| Similar to hypothetical protein FLJ10856 [Danio rerio] | TC88607 | 14 | 14 | 8.711 | 7.881 | 0.003 | 0.314 |
| unnamed protein product [Tetraodon nigroviridis] | CA378819 | 14 | 14 | 6.672 | 6.358 | 0.003 | 0.314 |
| similar to Melanoma-associated antigen C1 (MAGE-C1 antigen) | TC72891 | 14 | 14 | 14.058 | 14.507 | 0.003 | 0.314 |
| similar to RIKEN cDNA C230094B15 [Rattus norvegicus] | CA345414 | 14 | 14 | 7.968 | 8.399 | 0.003 | 0.314 |
| Metalloproteinase inhibitor 2 precursor (TIMP-2) (Tissue | TC72030 | 14 | 14 | 11.249 | 10.711 | 0.003 | 0.314 |
| Chromosome 19 open reading frame 28 [Homo sapiens] | CX037529 | 14 | 14 | 7.199 | 7.612 | 0.003 | 0.314 |

**Table S3.** *Cont.*

| **(e) Kidney: Brienzlig deformed (Pop1) *vs.* Brienzlig normal (Pop2)** |  |  |  |  |  |  |  |
| --- | --- | --- | --- | --- | --- | --- | --- |
| **Gene ID** | **Tigr ID** | **N_1_** | **N_2_** | **x_1_** | **x_2_** | ***P*** | **FDR** |
| Long-chain-fatty-acid--CoA ligase 5 (EC 6.2.1.3) (Long-chain | TC80487 | 14 | 14 | 6.783 | 7.012 | 0.003 | 0.314 |
| Arginyl aminopeptidase-like 1 (EC 3.4.11.-) (RNPEP-like | CF753073 | 14 | 14 | 8.636 | 9.149 | 0.003 | 0.314 |
| inositol polyphosphate-5-phosphatase, 145kDa; hp51CN [Homo sapiens] | TC80501 | 14 | 14 | 10.192 | 10.842 | 0.003 | 0.314 |
| IFT172 [Danio rerio] | CA360326 | 14 | 14 | 8.358 | 7.728 | 0.003 | 0.314 |
| Procollagen C-proteinase enhancer protein precursor (PCPE) | CX141492 | 14 | 14 | 8.732 | 9.297 | 0.003 | 0.314 |
| Transposable element TCB1 transposase (Transposable | TC86656 | 14 | 14 | 12.496 | 12.776 | 0.003 | 0.314 |
| Ictacalcin | BX914141 | 14 | 14 | 12.501 | 11.799 | 0.003 | 0.314 |
| Zinc finger and BTB domain containing protein 3 | BX302231 | 14 | 14 | 14.640 | 15.162 | 0.003 | 0.314 |
| UDP-glucuronosyltransferase 1-5 precursor, microsomal (EC | CA381172 | 14 | 14 | 7.698 | 7.965 | 0.003 | 0.314 |
| cytokine receptor CRFB5 [Tetraodon nigroviridis] | CX032074 | 14 | 14 | 8.660 | 9.007 | 0.003 | 0.314 |
| Zona pellucida sperm-binding protein 3 precursor (Zona | BX082732 | 14 | 14 | 6.346 | 6.802 | 0.003 | 0.314 |
| Myosin-binding protein C, cardiac-type (Cardiac MyBP-C) | BX911075 | 14 | 14 | 7.730 | 8.091 | 0.003 | 0.314 |
| similar to retinoblastoma-associated factor 600 [Rattus norvegicus] | TC71898 | 14 | 14 | 12.175 | 12.725 | 0.003 | 0.314 |
| Unknown (protein for MGC:85973); wu:fj46a05 [Danio rerio] | BX910562 | 14 | 14 | 8.358 | 8.850 | 0.003 | 0.314 |
| Double-stranded RNA-binding protein Staufen homolog | TC71599 | 14 | 14 | 13.130 | 13.554 | 0.003 | 0.314 |
| Vesicule-associated membrane protein 5 (VAMP-5) | CA381044 | 14 | 14 | 10.126 | 9.603 | 0.003 | 0.314 |
| Unknown (protein for IMAGE:7026099) [Xenopus tropicalis] | TC85563 | 14 | 14 | 7.229 | 7.655 | 0.003 | 0.314 |
| Lymphocyte cytosolic protein 2 (SH2 domain-containing | CA350126 | 14 | 14 | 9.570 | 9.178 | 0.003 | 0.314 |
| Putative secreted protein XAG precursor | TC78844 | 14 | 14 | 5.921 | 6.332 | 0.003 | 0.314 |
| Probable G protein-coupled receptor GPR1 | CA370002 | 14 | 14 | 7.128 | 6.469 | 0.003 | 0.314 |
| Dapper homolog 1 (hDPR1) (Heptacellular carcinoma novel | CX036471 | 14 | 14 | 7.883 | 8.293 | 0.003 | 0.314 |
| Claudin-like protein ZF4A22 (Claudin 7) | TC70038 | 14 | 14 | 6.508 | 7.040 | 0.003 | 0.314 |
| Circulating cathodic antigen (CCA) | TC70773 | 14 | 14 | 9.209 | 9.565 | 0.003 | 0.314 |
| unknown [Homo sapiens] | CA386387 | 14 | 14 | 7.019 | 6.725 | 0.003 | 0.314 |
| HOXA3A [Oryzias latipes] | AY567796 | 14 | 14 | 8.477 | 9.041 | 0.003 | 0.314 |
| DNA repair protein rad8 | TC84312 | 14 | 14 | 5.852 | 6.195 | 0.003 | 0.314 |
| Serine/threonine-protein kinase 17A (EC 2.7.1.37) (DAP | CX037883 | 14 | 14 | 8.218 | 7.551 | 0.003 | 0.314 |
| Ependymin precursor (EPD) | CA376554 | 14 | 14 | 13.200 | 12.592 | 0.003 | 0.314 |
| unnamed protein product [Tetraodon nigroviridis] | TC94560 | 14 | 14 | 8.870 | 8.053 | 0.003 | 0.314 |
| Kelch-like protein 3 | BX880813 | 14 | 14 | 6.606 | 6.365 | 0.003 | 0.314 |
| unnamed protein product [Tetraodon nigroviridis] | TC80950 | 14 | 14 | 7.808 | 8.333 | 0.004 | 0.314 |
| unnamed protein product [Tetraodon nigroviridis] | CA372324 | 14 | 14 | 8.584 | 8.927 | 0.004 | 0.314 |
| Ubiquitin-conjugating enzyme E2-28.4 kDa (EC 6.3.2.19) | TC87979 | 14 | 14 | 8.449 | 8.833 | 0.004 | 0.314 |
| Ubiquitin-conjugating enzyme E2-28.4 kDa (EC 6.3.2.19) | TC74710 | 14 | 14 | 7.113 | 7.523 | 0.004 | 0.314 |
| Chromobox protein homolog 3 (Heterochromatin protein 1 | TC86769 | 14 | 14 | 11.075 | 11.546 | 0.004 | 0.314 |
| class I helical cytokine receptor number 19 [Tetraodon | TC74829 | 14 | 14 | 8.563 | 7.802 | 0.004 | 0.314 |
| Activin receptor type I precursor (EC 2.7.1.37) (ACTR-I) | CA363719 | 14 | 14 | 7.808 | 8.218 | 0.004 | 0.314 |
| F-box/LRR-repeat protein 3A (F-box and leucine-rich repeat | TC82503 | 14 | 14 | 9.010 | 9.487 | 0.004 | 0.314 |
| Ependymin precursor (EPD) | CA380100 | 14 | 14 | 13.227 | 12.609 | 0.004 | 0.314 |
| CGI-90 protein [Homo sapiens] | BX872680 | 14 | 14 | 7.150 | 6.739 | 0.004 | 0.314 |
| Zinc finger protein 132 | TC90968 | 14 | 14 | 5.858 | 5.656 | 0.004 | 0.314 |
| Pyridoxine-5'-phosphate oxidase (EC 1.4.3.5) | TC75464 | 14 | 14 | 8.174 | 7.552 | 0.004 | 0.314 |
| similar to RIKEN cDNA B230339H12 [Rattus norvegicus] | TC82686 | 14 | 14 | 7.318 | 6.912 | 0.004 | 0.314 |

**Table S3.** *Cont.*

| **(e) Kidney: Brienzlig deformed (Pop1) *vs.* Brienzlig normal (Pop2)** |  |  |  |  |  |  |  |
| --- | --- | --- | --- | --- | --- | --- | --- |
| **Gene ID** | **Tigr ID** | **N_1_** | **N_2_** | **x_1_** | **x_2_** | ***P*** | **FDR** |
| Anaphase promoting complex subunit 4 (APC4) (Cyclosome | BX876798 | 14 | 14 | 8.027 | 8.688 | 0.004 | 0.314 |
| Aldehyde dehydrogenase 1A2 (EC 1.2.1.3) | TC72517 | 14 | 14 | 5.919 | 6.322 | 0.004 | 0.314 |
| unnamed protein product [Tetraodon nigroviridis] | CA375526 | 14 | 14 | 6.890 | 7.416 | 0.004 | 0.314 |
| Dual specificity mitogen-activated protein kinase kinase 7 | CA342333 | 14 | 14 | 5.771 | 5.434 | 0.004 | 0.314 |
| Phosphatidylinositol 3,4,5-trisphosphate-dependent Rac | CA351247 | 14 | 14 | 5.428 | 5.186 | 0.004 | 0.314 |
| CRK-associated substrate (p130Cas) (Breast cancer | CA362110 | 14 | 14 | 9.223 | 9.835 | 0.004 | 0.314 |
| PREDICTED: similar to expressed sequence AI256361 [Gallus gallus] | BX315440 | 14 | 14 | 9.445 | 10.084 | 0.004 | 0.314 |
| unnamed protein product [Tetraodon nigroviridis] | TC81426 | 14 | 14 | 8.696 | 9.189 | 0.004 | 0.314 |
| Transcription initiation factor TFIID subunit 4 | CX038427 | 14 | 14 | 6.810 | 6.686 | 0.004 | 0.314 |
| Fructose-bisphosphate aldolase A (EC 4.1.2.13) | TC78050 | 14 | 14 | 6.917 | 7.316 | 0.004 | 0.314 |
| Myogenin (Myogenic factor) | NP544079 | 14 | 14 | 8.623 | 9.186 | 0.004 | 0.314 |
| CMP-N-acetylneuraminate-beta-galactosamide-alpha-2, | TC87486 | 14 | 14 | 7.688 | 8.307 | 0.004 | 0.314 |
| cytochrome P450 [Oncorhynchus mykiss] | U82983 | 14 | 14 | 10.056 | 10.444 | 0.004 | 0.314 |
| PREDICTED: similar to meningioma expressed antigen 5 (hyaluronidase) | BX912183 | 14 | 14 | 6.370 | 6.740 | 0.004 | 0.314 |
| PREDICTED: similar to kinase non-catalytic C-lobe domain (KIND) | BX911989 | 14 | 14 | 6.930 | 7.353 | 0.004 | 0.314 |
| GPI-anchor transamidase precursor (EC 3.-.-.-) (GPI | TC81404 | 14 | 14 | 8.735 | 8.112 | 0.004 | 0.314 |
| HECT domain containing protein 1 (Fragment) | TC77628 | 14 | 14 | 11.282 | 11.858 | 0.004 | 0.314 |
| Spastin | BX913298 | 14 | 14 | 8.923 | 8.648 | 0.004 | 0.314 |
| MGC80272 protein [Xenopus laevis] | BX080799 | 14 | 14 | 5.628 | 5.856 | 0.004 | 0.314 |
| similar to interactor protein for cytohesin exchange factors 1 | CA366665 | 14 | 14 | 8.147 | 7.706 | 0.004 | 0.314 |
| Testis-specific gene A2 (Male meiotic metaphase | TC79924 | 14 | 14 | 8.374 | 8.749 | 0.004 | 0.314 |
| Metastasis suppressor protein 1 (Missing in metastasis | CA344048 | 14 | 14 | 14.985 | 15.355 | 0.004 | 0.314 |
| Unknown (protein for MGC:63962); wu:fe24b05 [Danio rerio] | CX031508 | 14 | 14 | 15.309 | 15.867 | 0.004 | 0.314 |
| Ubiquitin ligase protein PHF9 (EC 6.3.2.-) (FAAP 43) | TC81356 | 14 | 14 | 6.267 | 5.956 | 0.004 | 0.314 |
| Transcription intermediary factor 1-gamma (TIF1-gamma) | TC76601 | 14 | 14 | 5.566 | 5.319 | 0.004 | 0.314 |
| Pyridoxine-5'-phosphate oxidase (EC 1.4.3.5) | TC81189 | 14 | 14 | 9.012 | 8.276 | 0.004 | 0.314 |
| PREDICTED: similar to Plasmodium falciparum trophozoite antigen | TC75093 | 14 | 14 | 6.504 | 6.966 | 0.004 | 0.314 |
| Apoptosis regulator BAX, membrane isoform alpha | TC79870 | 14 | 14 | 11.543 | 12.106 | 0.004 | 0.314 |
| unnamed protein product [Tetraodon nigroviridis] | CA352062 | 14 | 14 | 6.582 | 6.917 | 0.004 | 0.314 |
| Ras-related protein Rab-21 | CR375980 | 14 | 14 | 9.685 | 10.343 | 0.004 | 0.314 |
| interferon-inducible protein Gig2 [Carassius auratus] | BX874048 | 14 | 14 | 11.409 | 11.746 | 0.004 | 0.314 |
| Conserved oligomeric Golgi complex component 2 (Low | TC71340 | 14 | 14 | 8.757 | 8.578 | 0.004 | 0.314 |
| Growth arrest and DNA-damage-inducible protein GADD45 beta | TC91840 | 14 | 14 | 6.832 | 6.247 | 0.004 | 0.314 |
| PREDICTED: similar to putative transmembrane protein TA-2 [Gallus | TC84045 | 14 | 14 | 8.741 | 9.235 | 0.004 | 0.314 |
| Ependymin precursor (EPD) | BX074005 | 14 | 14 | 14.086 | 13.550 | 0.004 | 0.314 |
| Carnitine O-palmitoyltransferase I, mitochondrial liver | NP543639 | 14 | 14 | 9.615 | 10.090 | 0.004 | 0.314 |
| unnamed protein product [Tetraodon nigroviridis] | BX086590 | 14 | 14 | 10.087 | 9.870 | 0.004 | 0.314 |
| Hypothetical RNA-binding protein C23E6.01c in chromosome | TC74127 | 14 | 14 | 10.513 | 10.224 | 0.004 | 0.314 |
| Zinc finger protein 189 | TC70596 | 14 | 14 | 11.272 | 10.935 | 0.004 | 0.314 |

**Table S3.** *Cont.*

| **(e) Kidney: Brienzlig deformed (Pop1) *vs.* Brienzlig normal (Pop2)** |  |  |  |  |  |  |  |
| --- | --- | --- | --- | --- | --- | --- | --- |
| **Gene ID** | **Tigr ID** | **N_1_** | **N_2_** | **x_1_** | **x_2_** | ***P*** | **FDR** |
| SON protein | TC70937 | 14 | 14 | 13.597 | 14.239 | 0.004 | 0.314 |
| ganglioside-induced differentiation-associated-protein 2 [Mus | BX867917 | 14 | 14 | 10.169 | 9.813 | 0.004 | 0.314 |
| Potassium channel subfamily K member 15 (Acid-sensitive | TC91162 | 14 | 14 | 6.779 | 7.245 | 0.004 | 0.314 |
| Zinc finger protein SLUG (Neural crest transcription | CA369439 | 14 | 14 | 10.581 | 11.401 | 0.004 | 0.314 |
| BDNF/NT-3 growth factors receptor precursor (EC 2.7.1.112) | TC85122 | 14 | 14 | 5.627 | 5.832 | 0.004 | 0.314 |
| unnamed protein product [Tetraodon nigroviridis] | BX876626 | 14 | 14 | 10.842 | 11.424 | 0.004 | 0.314 |
| Ski oncogene (C-ski) | TC90407 | 14 | 14 | 9.221 | 9.601 | 0.004 | 0.314 |
| Zinc finger protein 264 | TC70599 | 14 | 14 | 10.596 | 10.301 | 0.004 | 0.314 |
| Ictacalcin | CX026130 | 14 | 14 | 12.455 | 11.793 | 0.005 | 0.314 |
| Myoferlin (Fer-1 like protein 3) | BX317692 | 14 | 14 | 5.865 | 5.437 | 0.005 | 0.314 |
| unnamed protein product [Tetraodon nigroviridis] | TC94127 | 14 | 14 | 7.232 | 7.772 | 0.005 | 0.314 |
| NADH-ubiquinone oxidoreductase 15 kDa subunit (EC 1.6.5.3) | CX140792 | 14 | 14 | 8.396 | 8.671 | 0.005 | 0.314 |
| Delta-like protein 1 precursor (Drosophila Delta homolog 1) | TC80147 | 14 | 14 | 6.613 | 7.032 | 0.005 | 0.314 |
| Carbonic anhydrase-related protein 10 (Carbonic | CA345046 | 14 | 14 | 9.013 | 9.703 | 0.005 | 0.314 |
| Transcription factor jun-D | CA347362 | 14 | 14 | 9.514 | 9.008 | 0.005 | 0.314 |
| unnamed protein product [Tetraodon nigroviridis] | BX311200 | 14 | 14 | 5.574 | 5.903 | 0.005 | 0.314 |
| Zinc finger protein 502 | BX912311 | 14 | 14 | 11.837 | 11.695 | 0.005 | 0.314 |
| Eukaryotic initiation factor 4A-I (eIF4A-I) (eIF-4A-I) | TC86782 | 14 | 14 | 12.318 | 12.737 | 0.005 | 0.314 |
| Pro-neuregulin-1 precursor (Pro-NRG1) [Contains: | TC76125 | 14 | 14 | 9.263 | 9.740 | 0.005 | 0.314 |
| Unknown (protein for MGC:63842); wu:fb01d12 [Danio rerio] | CA370180 | 14 | 14 | 7.514 | 7.951 | 0.005 | 0.314 |
| Host cell factor C1 (HCF) (VP16 accessory protein) (HFC1) | CX029989 | 14 | 14 | 5.471 | 5.753 | 0.005 | 0.314 |
| Tumor protein D54 (hD54) (D52-like 2) | TC78808 | 14 | 14 | 7.210 | 7.417 | 0.005 | 0.314 |
| SI:zK76P14.4 (novel protein similar to human and rodent SRY (sex | BX304336 | 14 | 14 | 12.683 | 13.203 | 0.005 | 0.314 |
| hypothetical protein [Homo sapiens] | TC82589 | 14 | 14 | 12.654 | 13.100 | 0.005 | 0.314 |
| unnamed protein product [Tetraodon nigroviridis] | CA367011 | 14 | 14 | 6.654 | 6.897 | 0.005 | 0.314 |
| Growth arrest and DNA-damage-inducible protein GADD45 beta | CA369202 | 14 | 14 | 12.168 | 11.589 | 0.005 | 0.314 |
| Breakpoint cluster region protein (EC 2.7.1.-) | CR373407 | 14 | 14 | 13.639 | 14.262 | 0.005 | 0.314 |
| Transcription factor BTEB4 (Basic transcription element | CA377027 | 14 | 14 | 7.721 | 7.491 | 0.005 | 0.314 |
| transcription repressor p66 beta component of the MeCP1 complex [Homo | TC73931 | 14 | 14 | 9.204 | 9.590 | 0.005 | 0.314 |
| Golgi autoantigen, golgin subfamily A member 3 (Golgin-160) | CA356204 | 14 | 14 | 8.763 | 8.338 | 0.005 | 0.314 |
| PREDICTED: similar to cullin 4B [Gallus gallus] | CX043017 | 14 | 14 | 8.324 | 8.743 | 0.005 | 0.314 |
| Neural-cadherin precursor (N-cadherin) (Cadherin-2) | CA375357 | 14 | 14 | 7.610 | 8.084 | 0.005 | 0.314 |
| angiomotin like 1; junction-enriched and associated protein [Homo | BX873601 | 14 | 14 | 10.395 | 11.147 | 0.005 | 0.314 |
| Delta-like protein 4 precursor (Drosophila Delta homolog | CR375062 | 14 | 14 | 7.404 | 7.775 | 0.005 | 0.314 |
| Y+L amino acid transporter 1 (y(+)L-type amino acid | TC91734 | 14 | 14 | 9.334 | 8.946 | 0.005 | 0.314 |
| Beta-glucuronidase precursor (EC 3.2.1.31) | TC92330 | 14 | 14 | 9.906 | 9.461 | 0.005 | 0.314 |
| hypothetical protein [Gallus gallus] | TC73072 | 14 | 14 | 11.286 | 11.587 | 0.005 | 0.314 |
| ADP-ribosylation factor binding protein GGA3 | CA375275 | 14 | 14 | 12.846 | 12.623 | 0.005 | 0.314 |
| B-cell lymphoma 3-encoded protein (BCL-3 protein) | TC82369 | 14 | 14 | 10.131 | 10.515 | 0.005 | 0.314 |
| Regulator of G-protein signaling 1 (RGS1) (Early response | TC94714 | 14 | 14 | 11.180 | 10.627 | 0.005 | 0.314 |
| CDP-diacylglycerol--glycerol-3-phosphate | CA381649 | 14 | 14 | 7.381 | 7.672 | 0.005 | 0.314 |
| unnamed protein product [Tetraodon nigroviridis] | TC87686 | 14 | 14 | 8.826 | 8.363 | 0.005 | 0.314 |
| Retinoblastoma-associated protein (PP110) (P105-RB) (RB) | TC76532 | 14 | 14 | 5.508 | 5.781 | 0.005 | 0.314 |

**Table S3.** *Cont.*

| **(e) Kidney: Brienzlig deformed (Pop1) *vs.* Brienzlig normal (Pop2)** |  |  |  |  |  |  |  |
| --- | --- | --- | --- | --- | --- | --- | --- |
| **Gene ID** | **Tigr ID** | **N_1_** | **N_2_** | **x_1_** | **x_2_** | ***P*** | **FDR** |
| Mitogen-activated protein kinase kinase kinase 1 (EC | CA354801 | 14 | 14 | 9.273 | 9.725 | 0.005 | 0.314 |
| Ribonucleases P/MRP protein subunit POP1 (EC 3.1.26.5) | TC72640 | 14 | 14 | 11.836 | 12.427 | 0.005 | 0.314 |
| Cytosolic phospholipase A2 (CPLA2) [Includes: | CA342138 | 14 | 14 | 9.124 | 9.544 | 0.005 | 0.314 |
| P3 protein | TC88860 | 14 | 14 | 9.108 | 8.674 | 0.005 | 0.314 |
| Dual specificity mitogen-activated protein kinase kinase 7 | CA363096 | 14 | 14 | 6.544 | 6.028 | 0.005 | 0.314 |
| melatonin receptor [Oncorhynchus mykiss] | AF178538 | 14 | 14 | 6.481 | 6.799 | 0.005 | 0.314 |
| Guanine nucleotide-binding protein beta subunit 5 | TC81487 | 14 | 14 | 5.579 | 5.868 | 0.005 | 0.314 |
| MGC68567 protein [Xenopus laevis] | TC82533 | 14 | 14 | 8.188 | 7.764 | 0.006 | 0.314 |
| Ring canal kelch protein [Contains: Kelch short protein] | CA359161 | 14 | 14 | 10.771 | 11.128 | 0.006 | 0.314 |
| Protein-tyrosine phosphatase alpha precursor (EC 3.1.3.48) | TC94580 | 14 | 14 | 6.740 | 7.006 | 0.006 | 0.314 |
| unnamed protein product [Tetraodon nigroviridis] | TC70037 | 14 | 14 | 5.525 | 5.911 | 0.006 | 0.314 |
| Zinc finger protein 135 | CX027259 | 14 | 14 | 5.338 | 5.125 | 0.006 | 0.314 |
| Vacuolar protein sorting 26 (Vesicle protein sorting 26) | TC88058 | 14 | 14 | 9.353 | 9.116 | 0.006 | 0.314 |
| Cystine/glutamate transporter (Amino acid transport system | CA376717 | 14 | 14 | 10.088 | 10.577 | 0.006 | 0.314 |
| Angiopoietin 1 receptor precursor (EC 2.7.1.112) | TC85237 | 14 | 14 | 5.187 | 5.596 | 0.006 | 0.314 |
| AMP deaminase 2 (EC 3.5.4.6) (AMP deaminase isoform L) | TC82718 | 14 | 14 | 11.406 | 11.868 | 0.006 | 0.314 |
| M1 protein [Infectious hematopoietic necrosis virus] | AY673685 | 14 | 14 | 8.088 | 8.565 | 0.006 | 0.314 |
| Lymphocyte cytosolic protein 2 (SH2 domain-containing | TC91107 | 14 | 14 | 10.703 | 10.433 | 0.006 | 0.314 |
| ATP synthase B chain, mitochondrial precursor (EC | TC86548 | 14 | 14 | 8.434 | 8.873 | 0.006 | 0.314 |
| Retinoic acid-binding protein, cellular (CRABP) | TC80629 | 14 | 14 | 11.071 | 11.528 | 0.006 | 0.314 |
| Serine/threonine-protein kinase Sgk1 (EC 2.7.1.37) | AB011387 | 14 | 14 | 7.905 | 7.193 | 0.006 | 0.314 |
| transcription elongation regulator foggy; etID10129.15; etID37101.15 | TC70683 | 14 | 14 | 9.452 | 9.796 | 0.006 | 0.314 |
| Cytochrome P450 4B1 (EC 1.14.14.1) (CYPIVB1) (P450-isozyme | NP544121 | 14 | 14 | 10.088 | 10.465 | 0.006 | 0.314 |
| Tubby-like protein 4 (Tubby superfamily protein) | CA355514 | 14 | 14 | 8.252 | 8.862 | 0.006 | 0.314 |
| Splicing factor, arginine/serine-rich 7 (Splicing factor | TC79381 | 14 | 14 | 8.002 | 8.534 | 0.006 | 0.314 |
| TRAF-interacting protein | CX032230 | 14 | 14 | 7.413 | 7.590 | 0.006 | 0.314 |
| PREDICTED: similar to CXYorf1-related protein [Gallus gallus] | CA342436 | 14 | 14 | 9.215 | 9.780 | 0.006 | 0.314 |
| Growth hormone secretagogue receptor type 1 (GHS-R) | BX911634 | 14 | 14 | 9.293 | 9.748 | 0.006 | 0.314 |
| cAMP-specific 3',5'-cyclic phosphodiesterase 4B (EC | TC85319 | 14 | 14 | 8.953 | 9.559 | 0.006 | 0.314 |
| cardiac morphogenesis [Gallus gallus] | CA376994 | 14 | 14 | 10.214 | 10.884 | 0.006 | 0.314 |
| DNA cross-link repair 1A, PSO2 homolog; SNM1-like; DNA cross-link | TC84685 | 14 | 14 | 9.338 | 9.997 | 0.006 | 0.314 |
| Transcription factor xGATA-2 (GATA binding factor-2) | TC92316 | 14 | 14 | 6.459 | 7.061 | 0.006 | 0.314 |
| T-cell leukemia translocation-associated gene protein | TC81717 | 14 | 14 | 5.782 | 6.087 | 0.006 | 0.314 |
| Endothelin-converting enzyme 2 (EC 3.4.24.71) (ECE-2) | CR371854 | 14 | 14 | 8.740 | 9.179 | 0.006 | 0.314 |
| Trafficking protein particle complex subunit 6B | TC88493 | 14 | 14 | 6.102 | 5.376 | 0.006 | 0.314 |
| Ependymin precursor (EPD) | CA364579 | 14 | 14 | 14.250 | 13.685 | 0.006 | 0.314 |
| carnitine palmitoyl transferase I [Oncorhynchus mykiss] | AF327058 | 14 | 14 | 9.579 | 10.006 | 0.006 | 0.314 |
| Kinesin heavy chain isoform 5C (Kinesin heavy chain | TC74726 | 14 | 14 | 9.014 | 9.520 | 0.006 | 0.314 |
| aryl hydrocarbon receptor beta [Oncorhynchus mykiss] | AF065138 | 14 | 14 | 10.035 | 10.481 | 0.006 | 0.314 |
| Abhydrolase domain containing protein 2 (Protein PHPS1-2) | TC91044 | 14 | 14 | 5.954 | 5.740 | 0.006 | 0.314 |
| Kruppel-like factor 2 (Lung kruppel-like factor) | TC93427 | 14 | 14 | 11.533 | 11.026 | 0.006 | 0.314 |
| CC chemokine with stalk CK2 [Oncorhynchus mykiss] | AF418561 | 14 | 14 | 12.958 | 13.574 | 0.006 | 0.314 |

**Table S3.** *Cont.*

| **(e) Kidney: Brienzlig deformed (Pop1) *vs.* Brienzlig normal (Pop2)** |  |  |  |  |  |  |  |
| --- | --- | --- | --- | --- | --- | --- | --- |
| **Gene ID** | **Tigr ID** | **N_1_** | **N_2_** | **x_1_** | **x_2_** | ***P*** | **FDR** |
| Retrovirus-related Pol polyprotein from transposon 17.6 | TC92043 | 14 | 14 | 6.269 | 6.483 | 0.006 | 0.314 |
| Collagen alpha 1(XVI) chain precursor | TC84311 | 14 | 14 | 8.752 | 9.189 | 0.006 | 0.314 |
| Transposable element TCB1 transposase (Transposable | CB492059 | 14 | 14 | 14.150 | 14.420 | 0.006 | 0.314 |
| Dihydropyrimidinase related protein-2 (DRP-2) (Neural | TC70142 | 14 | 14 | 6.837 | 7.123 | 0.006 | 0.314 |
| Histone-lysine N-methyltransferase, H3 lysine-9 specific 5 | TC82516 | 14 | 14 | 11.388 | 11.878 | 0.006 | 0.314 |
| Calcineurin B subunit isoform 1 (Protein phosphatase 2B | TC72132 | 14 | 14 | 7.706 | 7.286 | 0.006 | 0.314 |
| Vacuolar protein sorting-associated protein 45 (rvps45) | TC83783 | 14 | 14 | 11.314 | 11.839 | 0.006 | 0.314 |
| myc homolog [Oncorhynchus mykiss] | S79770 | 14 | 14 | 9.634 | 9.248 | 0.006 | 0.314 |
| Achaete-scute homolog 1 (HASH1) | TC94407 | 14 | 14 | 6.665 | 7.069 | 0.006 | 0.314 |
| Hoxa-11b [Danio aequipinnatus] | AY567792 | 14 | 14 | 8.047 | 8.534 | 0.006 | 0.314 |
| Reticulon 1 (Neuroendocrine-specific protein) (S-rex) | TC87852 | 14 | 14 | 13.175 | 13.751 | 0.006 | 0.314 |
| S-adenosylmethionine synthetase gamma form (EC 2.5.1.6) | CX029922 | 14 | 14 | 8.884 | 9.448 | 0.006 | 0.314 |
| similar to Semaphorin 4C precursor (Semaphorin I) (Sema I) | CA345755 | 14 | 14 | 10.123 | 9.477 | 0.006 | 0.314 |
| Ras association domain family 1 isoform C; Ras association domain | CA375582 | 14 | 14 | 8.260 | 7.826 | 0.006 | 0.314 |
| Protein disulfide isomerase A3 precursor (EC 5.3.4.1) | BX301328 | 14 | 14 | 6.640 | 6.848 | 0.006 | 0.314 |
| Dermatomyositis associated with cancer putative | TC72261 | 14 | 14 | 10.188 | 9.970 | 0.006 | 0.314 |
| Calcium/calmodulin-dependent protein kinase type II gamma | TC87890 | 14 | 14 | 8.521 | 8.151 | 0.006 | 0.314 |
| 5-hydroxytryptamine 1A-alpha receptor (5-HT-1A-alpha) | CA378395 | 14 | 14 | 9.677 | 10.321 | 0.006 | 0.314 |
| H-2 class II histocompatibility antigen, A-Q alpha chain | CR376525 | 14 | 14 | 9.544 | 8.846 | 0.006 | 0.314 |
| Transcription factor jun-B | TC77803 | 14 | 14 | 9.517 | 8.791 | 0.006 | 0.314 |
| Maintenance of ploidy protein mob1 | TC91626 | 14 | 14 | 10.474 | 10.062 | 0.006 | 0.314 |
| Diaphanous protein homolog 1 (Diaphanous-related formin 1) | CX027034 | 14 | 14 | 6.026 | 5.566 | 0.006 | 0.314 |
| Furin precursor (EC 3.4.21.75) (Paired basic amino acid | BX864224 | 14 | 14 | 6.687 | 7.091 | 0.006 | 0.314 |
| Unknown (protein for MGC:98862) [Xenopus laevis] | TC73412 | 14 | 14 | 10.304 | 10.721 | 0.006 | 0.314 |
| NAD-dependent deacetylase sirtuin 2 (EC 3.5.1.-) | TC81509 | 14 | 14 | 7.949 | 7.477 | 0.006 | 0.314 |
| similar to WD repeat domain 11 protein [Rattus norvegicus] | CA355437 | 14 | 14 | 6.715 | 6.953 | 0.006 | 0.314 |
| Postreplication repair protein RAD18 (hRAD18) (hHR18) | BX868977 | 14 | 14 | 5.568 | 5.861 | 0.006 | 0.314 |
| Ependymin precursor (EPD) | CA359989 | 14 | 14 | 13.926 | 13.372 | 0.006 | 0.314 |
| nudE nuclear distribution gene E homolog like 1 (A. nidulans) B | TC73567 | 14 | 14 | 9.419 | 10.166 | 0.006 | 0.314 |
| unnamed protein product [Tetraodon nigroviridis] | TC71315 | 14 | 14 | 11.127 | 10.701 | 0.006 | 0.314 |
| PREDICTED: similar to RIKEN cDNA 2410129H14 [Homo sapiens] | TC90479 | 14 | 14 | 12.873 | 13.184 | 0.006 | 0.314 |
| Vascular endothelial growth factor A precursor (VEGF-A) | NP1132134 | 14 | 14 | 7.968 | 8.328 | 0.006 | 0.314 |
| Growth/differentiation factor 9 precursor (GDF-9) | TC83094 | 14 | 14 | 10.655 | 11.289 | 0.006 | 0.314 |
| myosin heavy chain [Oncorhynchus mykiss] | Z48794 | 14 | 14 | 6.562 | 6.876 | 0.006 | 0.314 |
| Fatty acid-binding protein, intestinal (I-FABP) (FABPI) | TC86688 | 14 | 14 | 7.109 | 6.104 | 0.006 | 0.314 |
| CC chemokine [Triakis scyllium] | CA371157 | 14 | 14 | 10.168 | 10.668 | 0.006 | 0.314 |
| NADP-dependent malic enzyme, mitochondrial precursor (EC | BX865089 | 14 | 14 | 7.942 | 8.408 | 0.007 | 0.314 |
| Nicotinate-nucleotide pyrophosphorylase [carboxylating] | TC75160 | 14 | 14 | 9.313 | 9.952 | 0.007 | 0.314 |
| Nuclear receptor ROR-beta (Nuclear receptor RZR-beta) | BX864215 | 14 | 14 | 7.132 | 7.619 | 0.007 | 0.314 |
| Dipeptidyl-peptidase II precursor (EC 3.4.14.2) (DPP II) | TC79683 | 14 | 14 | 11.481 | 10.963 | 0.007 | 0.314 |
| Oligophrenin 1 | BX320269 | 14 | 14 | 8.485 | 8.150 | 0.007 | 0.314 |
| prickle-like 1; REST (RE-1 silencing transcription factor)/NRSF | CA375840 | 14 | 14 | 5.717 | 6.017 | 0.007 | 0.314 |
| unnamed protein product [Tetraodon nigroviridis] | BX886442 | 14 | 14 | 6.849 | 7.348 | 0.007 | 0.314 |

**Table S3.** *Cont.*

| **(e) Kidney: Brienzlig deformed (Pop1) *vs.* Brienzlig normal (Pop2)** |  |  |  |  |  |  |  |
| --- | --- | --- | --- | --- | --- | --- | --- |
| **Gene ID** | **Tigr ID** | **N_1_** | **N_2_** | **x_1_** | **x_2_** | ***P*** | **FDR** |
| unnamed protein product [Tetraodon nigroviridis] | AY772711 | 14 | 14 | 8.129 | 8.720 | 0.007 | 0.314 |
| TPA: transposase [Rana pipiens] | CR370567 | 14 | 14 | 9.876 | 9.562 | 0.007 | 0.314 |
| Fibroleukin precursor (Fibrinogen-like protein 2) (pT49) | TC79840 | 14 | 14 | 9.395 | 8.647 | 0.007 | 0.314 |
| unnamed protein product [Tetraodon nigroviridis] | CA382997 | 14 | 14 | 8.554 | 9.020 | 0.007 | 0.314 |
| Polypyrimidine tract-binding protein 1 (PTB) | TC70383 | 14 | 14 | 10.828 | 11.309 | 0.007 | 0.314 |
| N-acylsphingosine amidohydrolase-like precursor (EC | TC72360 | 14 | 14 | 10.463 | 9.986 | 0.007 | 0.314 |
| hypothetical protein PP1665 [Homo sapiens] | TC81986 | 14 | 14 | 5.534 | 5.808 | 0.007 | 0.314 |
| DNA-directed RNA polymerase II largest subunit (EC 2.7.7.6) | CX136714 | 14 | 14 | 8.634 | 9.274 | 0.007 | 0.314 |
| unnamed protein product [Tetraodon nigroviridis] | TC72947 | 14 | 14 | 7.682 | 8.005 | 0.007 | 0.314 |
| Excitatory amino acid transporter 2 (Sodium-dependent | TC81908 | 14 | 14 | 9.428 | 9.989 | 0.007 | 0.314 |
| Laminin beta-3 chain precursor (Laminin 5 beta 3) (Laminin | TC92241 | 14 | 14 | 5.848 | 6.104 | 0.007 | 0.314 |
| Putative RNA-binding protein 15 (RNA binding motif protein | CA384725 | 14 | 14 | 7.240 | 6.967 | 0.007 | 0.314 |
| Thyroid hormone receptor-associated protein complex 230 kDa | TC92996 | 14 | 14 | 10.583 | 10.877 | 0.007 | 0.314 |
| SBBI26 protein; kelch/BTB; kelch (Drosophila)-like 6 [Homo sapiens] | TC77068 | 14 | 14 | 7.339 | 7.704 | 0.007 | 0.314 |
| Paxillin | BX310814 | 14 | 14 | 11.522 | 12.265 | 0.007 | 0.314 |
| C5a receptor [Oncorhynchus mykiss] | AY366354 | 14 | 14 | 7.422 | 7.871 | 0.007 | 0.314 |
| PREDICTED: similar to KSHV latent nuclear antigen interacting | TC77316 | 14 | 14 | 7.916 | 8.236 | 0.007 | 0.314 |
| PREDICTED: similar to Solute carrier family 22 (organic cation | TC82324 | 14 | 14 | 9.389 | 9.672 | 0.007 | 0.314 |
| wbscr21-like [Danio rerio] | BX311974 | 14 | 14 | 11.730 | 12.306 | 0.007 | 0.314 |
| Cystathionine beta-synthase (EC 4.2.1.22) (Serine | BX868751 | 14 | 14 | 8.294 | 7.762 | 0.007 | 0.314 |
| Ephrin type-B receptor 3 precursor (EC 2.7.1.112) | CA343874 | 14 | 14 | 5.825 | 6.089 | 0.007 | 0.314 |
| PREDICTED: similar to FLJ45273 protein [Gallus gallus] | BX874052 | 14 | 14 | 9.785 | 9.396 | 0.007 | 0.314 |
| Protein tyrosine phosphatase, non-receptor type 14 (EC | BX309617 | 14 | 14 | 9.132 | 9.781 | 0.007 | 0.314 |
| Alpha-2-macroglobulin precursor (Alpha-2-M) | TC76049 | 14 | 14 | 9.262 | 9.694 | 0.007 | 0.314 |
| CUG triplet repeat RNA-binding protein 1 (CUG-BP1) | CA379033 | 14 | 14 | 6.681 | 6.233 | 0.007 | 0.314 |
| Aquaporin 4 (WCH4) (Mercurial-insensitive water channel) | BX880542 | 14 | 14 | 11.636 | 12.298 | 0.007 | 0.314 |
| 26S proteasome non-ATPase regulatory subunit 1 (26S | CA384390 | 14 | 14 | 11.042 | 11.647 | 0.007 | 0.314 |
| BTB/POZ domain containing protein 9 | CR373080 | 14 | 14 | 14.187 | 14.522 | 0.007 | 0.314 |
| Jumonji domain containing protein 2A | TC85843 | 14 | 14 | 5.813 | 5.508 | 0.007 | 0.314 |
| gremlin [Gallus gallus] | BX863439 | 14 | 14 | 7.972 | 8.439 | 0.007 | 0.314 |
| Cathepsin L2 precursor (EC 3.4.22.43) (Cathepsin V) | CF753036 | 14 | 14 | 9.960 | 9.357 | 0.007 | 0.314 |
| A kinase anchor protein 1, mitochondrial precursor (Protein | TC81058 | 14 | 14 | 11.828 | 12.525 | 0.007 | 0.314 |
| Baculoviral IAP repeat-containing protein 4 (Inhibitor of | TC74466 | 14 | 14 | 6.806 | 7.021 | 0.007 | 0.314 |
| Vesicle-associated membrane protein 3 (VAMP-3) | BX073534 | 14 | 14 | 10.921 | 10.399 | 0.007 | 0.314 |
| unnamed protein product [Tetraodon nigroviridis] | TC87541 | 14 | 14 | 9.949 | 9.562 | 0.007 | 0.314 |
| smoothelin, like [Danio rerio] | CA381032 | 14 | 14 | 8.577 | 9.119 | 0.007 | 0.314 |
| Epithelial protein lost in neoplasm | CX030074 | 14 | 14 | 7.483 | 7.943 | 0.007 | 0.314 |
| unnamed protein product [Tetraodon nigroviridis] | TC85723 | 14 | 14 | 7.428 | 7.057 | 0.007 | 0.314 |
| NO145 protein [Xenopus laevis] | TC76100 | 14 | 14 | 8.029 | 8.575 | 0.007 | 0.314 |
| Zonadhesin precursor | CA368785 | 14 | 14 | 9.299 | 9.853 | 0.007 | 0.314 |
| PREDICTED: similar to serine/threonine protein kinase MASK; | TC89158 | 14 | 14 | 10.418 | 10.139 | 0.007 | 0.314 |
| Procollagen C-proteinase enhancer protein precursor (PCPE) | TC89456 | 14 | 14 | 7.893 | 8.415 | 0.007 | 0.314 |
| Putative sodium/calcium exchanger 7 precursor | CA387416 | 14 | 14 | 10.304 | 9.874 | 0.007 | 0.314 |

**Table S3.** *Cont.*

| **(e) Kidney: Brienzlig deformed (Pop1) *vs.* Brienzlig normal (Pop2)** |  |  |  |  |  |  |  |
| --- | --- | --- | --- | --- | --- | --- | --- |
| **Gene ID** | **Tigr ID** | **N_1_** | **N_2_** | **x_1_** | **x_2_** | ***P*** | **FDR** |
| Forkhead box protein F2 (Forkhead-related protein FKHL6) | TC77941 | 14 | 14 | 5.377 | 5.670 | 0.007 | 0.314 |
| ADAM 9 precursor (EC 3.4.24.-) (A disintegrin and | TC80951 | 14 | 14 | 8.951 | 9.614 | 0.007 | 0.314 |
| Copine III | CA365302 | 14 | 14 | 9.222 | 9.883 | 0.007 | 0.314 |
| Thyroid transcription factor 1 (Thyroid nuclear factor 1) | BX913961 | 14 | 14 | 8.950 | 9.555 | 0.007 | 0.314 |
| PREDICTED: similar to NN8-4AG [Gallus gallus] | BX873494 | 14 | 14 | 6.995 | 7.341 | 0.007 | 0.314 |
| Integrin beta-1* precursor | TC91714 | 14 | 14 | 10.335 | 10.728 | 0.007 | 0.314 |
| Solute carrier family 2, facilitated glucose transporter, | CA380232 | 14 | 14 | 11.984 | 12.510 | 0.007 | 0.314 |
| Zona pellucida sperm-binding protein 3 precursor (Zona | TC78501 | 14 | 14 | 7.748 | 8.328 | 0.007 | 0.314 |
| GalNAc alpha 2,6-sialyltransferase [Oncorhynchus mykiss] | AB097943 | 14 | 14 | 7.256 | 7.611 | 0.007 | 0.314 |
| Nuclear factor erythroid 2 related factor 2 (NF-E2 related | TC72590 | 14 | 14 | 10.163 | 9.668 | 0.007 | 0.314 |
| zgc:92407 [Danio rerio] | TC91473 | 14 | 14 | 9.730 | 10.416 | 0.007 | 0.314 |
| Ependymin precursor (EPD) | CA349886 | 14 | 14 | 9.433 | 8.866 | 0.007 | 0.314 |
| Claudin-15 | TC69835 | 14 | 14 | 9.195 | 9.562 | 0.007 | 0.314 |
| Acyl-protein thioesterase 2 (EC 3.1.2.-) | TC70460 | 14 | 14 | 9.895 | 10.468 | 0.007 | 0.314 |
| Bifunctional UDP-N-acetylglucosamine | TC75783 | 14 | 14 | 8.029 | 7.610 | 0.007 | 0.314 |
| Pyridoxine-5'-phosphate oxidase (EC 1.4.3.5) | BX866992 | 14 | 14 | 8.636 | 8.174 | 0.007 | 0.314 |
| Cystic fibrosis transmembrane conductance regulator (CFTR) | BX079994 | 14 | 14 | 8.953 | 9.606 | 0.007 | 0.314 |
| zgc:91805 [Danio rerio] | TC84452 | 14 | 14 | 5.895 | 6.140 | 0.007 | 0.314 |
| Ependymin precursor (EPD) | CA367528 | 14 | 14 | 14.080 | 13.526 | 0.007 | 0.314 |
| Brain mitochondrial carrier protein-1 (BMCP-1) | CX034155 | 14 | 14 | 5.591 | 5.332 | 0.007 | 0.314 |
| Sentrin-specific protease 6 (EC 3.4.22.-) | CR369733 | 14 | 14 | 9.724 | 10.288 | 0.007 | 0.314 |
| hypothetical protein zgc:85938 [Danio rerio] | TC78703 | 14 | 14 | 11.867 | 12.438 | 0.007 | 0.314 |
| Fibroblast growth factor-23 precursor (FGF-23) | BX307861 | 14 | 14 | 8.612 | 9.067 | 0.007 | 0.314 |
| Oxysterol binding protein-related protein 9 (OSBP-related | TC89775 | 14 | 14 | 8.010 | 7.737 | 0.007 | 0.314 |
| unnamed protein product [Tetraodon nigroviridis] | CA356364 | 14 | 14 | 9.649 | 10.171 | 0.007 | 0.314 |
| Probable Bax inhibitor-1 (BI-1) | CA360485 | 14 | 14 | 10.773 | 10.411 | 0.007 | 0.314 |
| Bovine leukemia virus cell receptor precursor (BLV-R) | BX885137 | 14 | 14 | 8.434 | 8.915 | 0.007 | 0.314 |
| Aristaless related homeobox protein (ARX) | TC92707 | 14 | 14 | 5.302 | 5.551 | 0.007 | 0.314 |
| Pancreasin precursor (EC 3.4.21.-) (Marapsin) | TC72728 | 14 | 14 | 9.162 | 9.765 | 0.008 | 0.314 |
| Ubiquitin carboxyl-terminal hydrolase 10 (EC 3.1.2.15) | CX029364 | 14 | 14 | 8.221 | 8.773 | 0.008 | 0.314 |
| Phosphatidylinositol 3-kinase regulatory alpha subunit | TC84829 | 14 | 14 | 7.202 | 7.626 | 0.008 | 0.314 |
| carbonic anhydrase VIII [Homo sapiens] | AY860422 | 14 | 14 | 8.226 | 9.050 | 0.008 | 0.314 |
| unnamed protein product [Tetraodon nigroviridis] | TC81970 | 14 | 14 | 7.186 | 7.677 | 0.008 | 0.314 |
| Ependymin precursor (EPD) | CA350572 | 14 | 14 | 9.407 | 8.853 | 0.008 | 0.314 |
| NF-kappaB essential modulator (NEMO) (NF-kappaB essential | TC88788 | 14 | 14 | 9.604 | 9.258 | 0.008 | 0.314 |
| inositol polyphosphate-5-phosphatase D; Inositol | CA383331 | 14 | 14 | 9.154 | 9.580 | 0.008 | 0.314 |
| PREDICTED: similar to RIKEN cDNA 8030451K01 [Gallus gallus] | BX298729 | 14 | 14 | 10.183 | 9.763 | 0.008 | 0.314 |
| Ependymin precursor (EPD) | CX139056 | 14 | 14 | 14.141 | 13.589 | 0.008 | 0.314 |
| Ig heavy chain V region MOPC 141 precursor | TC88582 | 14 | 14 | 5.315 | 5.708 | 0.008 | 0.314 |
| PREDICTED: similar to testis specific leucine rich repeat protein | CR371929 | 14 | 14 | 8.738 | 9.391 | 0.008 | 0.314 |
| cartilage acidic protein 1; chondrocyte expressed protein 68 kDa | TC85515 | 14 | 14 | 11.041 | 11.615 | 0.008 | 0.314 |
| Transcription initiation factor TFIID subunit 5 | TC72706 | 14 | 14 | 9.554 | 10.046 | 0.008 | 0.314 |
| Kinesin-like protein KIF3A (Microtubule plus end-directed | CX040668 | 14 | 14 | 5.984 | 5.725 | 0.008 | 0.314 |

**Table S3.** *Cont.*

| **(e) Kidney: Brienzlig deformed (Pop1) *vs.* Brienzlig normal (Pop2)** |  |  |  |  |  |  |  |
| --- | --- | --- | --- | --- | --- | --- | --- |
| **Gene ID** | **Tigr ID** | **N_1_** | **N_2_** | **x_1_** | **x_2_** | ***P*** | **FDR** |
| IL-8 receptor [Oncorhynchus mykiss] | AF260961 | 14 | 14 | 7.953 | 8.467 | 0.008 | 0.314 |
| Receptor-type protein-tyrosine phosphatase N2 precursor (EC | CA354615 | 14 | 14 | 11.581 | 12.205 | 0.008 | 0.314 |
| Molybdenum cofactor synthesis protein 2 large subunit | TC89039 | 14 | 14 | 8.381 | 8.893 | 0.008 | 0.314 |
| Ryanodine receptor 3 (Brain-type ryanodine receptor) (RyR3) | CA372174 | 14 | 14 | 9.579 | 10.066 | 0.008 | 0.314 |
| Deltex protein 3 (Deltex-3) (Deltex3) | BX886178 | 14 | 14 | 8.513 | 7.807 | 0.008 | 0.314 |
| Dual specificity protein kinase CLK2 (EC 2.7.1.37) (EC | CA361831 | 14 | 14 | 5.761 | 5.436 | 0.008 | 0.314 |
| DGCR8 protein (DiGeorge syndrome critical region 8) | TC72907 | 14 | 14 | 6.170 | 5.891 | 0.008 | 0.314 |
| Transcription initiation factor TFIID subunit 12 | TC78781 | 14 | 14 | 13.183 | 12.933 | 0.008 | 0.314 |
| src associated in mitosis, 68 kDa [Rattus norvegicus] | CA365731 | 14 | 14 | 8.522 | 8.924 | 0.008 | 0.314 |
| PREDICTED: similar to hypothetical protein FLJ22169, partial [Gallus | TC90298 | 14 | 14 | 7.703 | 8.127 | 0.008 | 0.314 |
| zgc:91859 [Danio rerio] | TC71199 | 14 | 14 | 7.681 | 8.081 | 0.008 | 0.314 |
| PREDICTED: similar to vimentin-type intermediate filament | TC80395 | 14 | 14 | 7.261 | 7.060 | 0.008 | 0.314 |
| annexin A13; annexin 13; ANX XIII [Danio rerio] | CA347209 | 14 | 14 | 7.936 | 8.503 | 0.008 | 0.314 |
| X box binding protein-1 (XBP-1) (TREB5 protein) | TC81242 | 14 | 14 | 8.600 | 7.742 | 0.008 | 0.314 |
| 5-day ovary-specific transcript 1 [Mus musculus] | CX029561 | 14 | 14 | 7.161 | 7.583 | 0.008 | 0.314 |
| vascular endothelial growth factor precursor [Oncorhynchus mykiss] | AJ717302 | 14 | 14 | 7.927 | 8.279 | 0.008 | 0.314 |
| Gamma adducin (Adducin-like protein 70) | CA359925 | 14 | 14 | 5.875 | 5.512 | 0.008 | 0.314 |
| similar to TMEFF2 [Rattus norvegicus] | CA369432 | 14 | 14 | 7.884 | 8.471 | 0.008 | 0.314 |
| Inositol 1,4,5-trisphosphate receptor type 3 (Type 3 | TC72886 | 14 | 14 | 6.990 | 7.293 | 0.008 | 0.314 |
| unnamed protein product [Tetraodon nigroviridis] | TC81410 | 14 | 14 | 5.880 | 6.222 | 0.008 | 0.314 |
| unnamed protein product [Tetraodon nigroviridis] | CA351432 | 14 | 14 | 12.022 | 11.716 | 0.008 | 0.314 |
| PREDICTED: similar to Arrdc1 protein [Gallus gallus] | BX296913 | 14 | 14 | 8.409 | 8.828 | 0.008 | 0.314 |
| unnamed protein product [Tetraodon nigroviridis] | TC89930 | 14 | 14 | 12.177 | 12.757 | 0.008 | 0.314 |
| Similar to hypothetical protein, estradiol-induced; wu:fd51d02 | BX301157 | 14 | 14 | 11.115 | 11.749 | 0.008 | 0.314 |
| TGF-beta receptor type II precursor (EC 2.7.1.37) (TGFR-2) | TC91098 | 14 | 14 | 5.811 | 6.240 | 0.008 | 0.314 |
| high mobility group AT-hook 1-like 4; PAP-1 binding protein [Homo | TC89791 | 14 | 14 | 11.782 | 12.110 | 0.008 | 0.314 |
| Multidrug resistance-associated protein 1 | CA381297 | 14 | 14 | 9.011 | 9.501 | 0.008 | 0.314 |
| Lysophosphatidic acid receptor Edg-2 (LPA receptor 1) | BX865598 | 14 | 14 | 6.674 | 7.179 | 0.008 | 0.314 |
| Vacuolar ATP synthase 16 kDa proteolipid subunit (EC | CA360789 | 14 | 14 | 12.415 | 11.994 | 0.008 | 0.314 |
| EGF-containing fibulin-like extracellular matrix protein 1 | TC79770 | 14 | 14 | 8.910 | 9.328 | 0.008 | 0.314 |
| GRB2-associated binding protein 2 (GRB2-associated | CA372416 | 14 | 14 | 8.553 | 9.177 | 0.008 | 0.314 |
| Dynein light intermediate chain 1, cytosolic (LIC57/59) | TC77535 | 14 | 14 | 8.117 | 8.648 | 0.008 | 0.314 |
| RAS guanyl releasing protein 3 (Calcium and DAG-regulated | TC75017 | 14 | 14 | 7.150 | 6.697 | 0.008 | 0.314 |
| Regulator of G-protein signaling 1 (RGS1) | TC72219 | 14 | 14 | 13.936 | 13.083 | 0.008 | 0.314 |
| unnamed protein product [Tetraodon nigroviridis] | BX910766 | 14 | 14 | 8.164 | 8.396 | 0.008 | 0.314 |
| unnamed protein product [Tetraodon nigroviridis] | TC82411 | 14 | 14 | 7.973 | 8.344 | 0.008 | 0.314 |
| zgc:92798 [Danio rerio] | BX316182 | 14 | 14 | 7.334 | 7.781 | 0.008 | 0.314 |
| unnamed protein product [Tetraodon nigroviridis] | TC83858 | 14 | 14 | 9.559 | 10.118 | 0.008 | 0.314 |
| unnamed protein product [Tetraodon nigroviridis] | TC92919 | 14 | 14 | 6.270 | 6.707 | 0.008 | 0.314 |
| 26S proteasome non-ATPase regulatory subunit 3 (26S | CX034469 | 14 | 14 | 9.424 | 9.848 | 0.008 | 0.314 |
| Ependymin precursor (EPD) | CA384886 | 14 | 14 | 13.194 | 12.623 | 0.008 | 0.314 |

**Table S3.** *Cont.*

| **(e) Kidney: Brienzlig deformed (Pop1) *vs.* Brienzlig normal (Pop2)** |  |  |  |  |  |  |  |
| --- | --- | --- | --- | --- | --- | --- | --- |
| **Gene ID** | **Tigr ID** | **N_1_** | **N_2_** | **x_1_** | **x_2_** | ***P*** | **FDR** |
| Sulfate transporter (Diastrophic dysplasia protein) | CA362175 | 14 | 14 | 8.261 | 8.837 | 0.008 | 0.314 |
| Ankyrin repeat and SAM domain containing protein 1 | TC80211 | 14 | 14 | 12.196 | 12.673 | 0.009 | 0.314 |
| unnamed protein product [Tetraodon nigroviridis] | TC82538 | 14 | 14 | 11.062 | 11.378 | 0.009 | 0.314 |
| Ig heavy chain V-I region HG3 precursor | BX877126 | 14 | 14 | 9.058 | 9.537 | 0.009 | 0.314 |
| unnamed protein product [Tetraodon nigroviridis] | CA384709 | 14 | 14 | 9.838 | 10.194 | 0.009 | 0.314 |
| Cystathionine beta-synthase (EC 4.2.1.22) (Serine | CA384193 | 14 | 14 | 8.306 | 7.757 | 0.009 | 0.314 |
| Metastasis associated protein MTA2 (Metastasis-associated | TC87106 | 14 | 14 | 8.666 | 9.117 | 0.009 | 0.314 |
| Gamma-aminobutyric-acid receptor alpha-4 subunit precursor | CX036461 | 14 | 14 | 6.457 | 6.817 | 0.009 | 0.314 |
| Ankyrin 1 (Erythrocyte ankyrin) (Ankyrin R) | TC87071 | 14 | 14 | 11.133 | 11.530 | 0.009 | 0.314 |
| Serine palmitoyltransferase 1 (EC 2.3.1.50) (Long chain base | TC87684 | 14 | 14 | 9.714 | 9.459 | 0.009 | 0.314 |
| Myosin light chain 3, skeletal muscle isoform (A2 | TC70049 | 14 | 14 | 6.960 | 7.418 | 0.009 | 0.314 |
| matrix metalloproteinase 14 (membrane-inserted) beta; zMT-MMP-beta | CF752733 | 14 | 14 | 9.642 | 10.232 | 0.009 | 0.314 |
| Apoptosis regulator NR-13 | TC71646 | 14 | 14 | 12.149 | 11.769 | 0.009 | 0.314 |
| Baculoviral IAP repeat-containing protein 6 | BX080890 | 14 | 14 | 14.379 | 14.723 | 0.009 | 0.314 |
| Ependymin precursor (EPD) | BX082806 | 14 | 14 | 13.921 | 13.287 | 0.009 | 0.314 |
| Pum1-A protein [Xenopus laevis] | AB211230 | 14 | 14 | 8.811 | 9.349 | 0.009 | 0.314 |
| Sorting nexin 27 | CA347540 | 14 | 14 | 7.218 | 6.762 | 0.009 | 0.314 |
| procathepsin B [Oncorhynchus mykiss] | AF358667 | 14 | 14 | 10.600 | 11.200 | 0.009 | 0.314 |
| Heparin sulfate N-deacetylase/N-sulfotransferase (EC | TC91060 | 14 | 14 | 8.424 | 8.971 | 0.009 | 0.314 |
| RNA-binding region containing protein 1 (ssDNA binding | TC88694 | 14 | 14 | 11.922 | 12.551 | 0.009 | 0.314 |
| 60S ribosomal protein L31 | TC69753 | 14 | 14 | 5.850 | 6.108 | 0.009 | 0.314 |
| Transmembrane protease, serine 6 (EC 3.4.21.-) | BX889676 | 14 | 14 | 6.840 | 7.333 | 0.009 | 0.314 |
| Probable GTP-binding protein engB | TC91909 | 14 | 14 | 10.609 | 11.315 | 0.009 | 0.314 |
| Fructose-1,6-bisphosphatase isozyme 2 (EC 3.1.3.11) | TC78043 | 14 | 14 | 8.353 | 8.866 | 0.009 | 0.314 |
| Zinc finger protein 288 (Dendritic-derived BTB/POZ zinc | CA363456 | 14 | 14 | 6.377 | 5.957 | 0.009 | 0.314 |
| TBC1 domain family member 4 | TC72343 | 14 | 14 | 7.799 | 8.315 | 0.009 | 0.314 |
| Zinc finger protein 28 homolog (Zfp-28) (Kruppel-like zinc | BX318271 | 14 | 14 | 9.456 | 9.068 | 0.009 | 0.314 |
| Adrenal medulla 50 kDa protein | TC73424 | 14 | 14 | 8.887 | 9.198 | 0.009 | 0.314 |
| PREDICTED: similar to tomosyn [Gallus gallus] | CA376445 | 14 | 14 | 12.918 | 13.342 | 0.009 | 0.314 |
| Epoxide hydrolase 1 (EC 3.3.2.3) (Microsomal epoxide | TC71090 | 14 | 14 | 11.210 | 11.585 | 0.009 | 0.314 |
| unnamed protein product [Homo sapiens] | BX869771 | 14 | 14 | 9.108 | 9.564 | 0.009 | 0.314 |
| Adiponutrin | BX318924 | 14 | 14 | 10.897 | 11.473 | 0.009 | 0.314 |
| MYST histone acetyltransferase 2 [Mus musculus] | CA346235 | 14 | 14 | 5.475 | 5.227 | 0.009 | 0.314 |
| Rho guanine nucleotide exchange factor 6 (PAK-interacting | CA386072 | 14 | 14 | 5.238 | 5.505 | 0.009 | 0.314 |
| Clathrin coat assembly protein AP180 (Clathrin coat | TC72173 | 14 | 14 | 11.792 | 12.319 | 0.009 | 0.314 |
| Retrovirus-related Pol polyprotein from transposon opus | CX142445 | 14 | 14 | 8.268 | 8.755 | 0.009 | 0.314 |
| PREDICTED: similar to Zinc finger protein Rlf (Rearranged L-myc | TC92073 | 14 | 14 | 12.098 | 12.688 | 0.009 | 0.314 |
| Splicing factor 3A subunit 3 (Spliceosome associated protein | TC70438 | 14 | 14 | 10.944 | 10.705 | 0.009 | 0.314 |
| Tankyrase 2 (EC 2.4.2.30) (TANK2) (Tankyrase II) (TNKS-2) | CX039917 | 14 | 14 | 6.090 | 6.296 | 0.009 | 0.314 |
| Zgc:100929 protein [Danio rerio] | CA354146 | 14 | 14 | 11.695 | 12.300 | 0.009 | 0.314 |
| Small nuclear ribonucleoprotein Sm D2 (snRNP core protein | TC87012 | 14 | 14 | 10.514 | 11.012 | 0.009 | 0.314 |
| Keratin, type I cytoskeletal 18 (Cytokeratin 18) (K18) (CK | CA352546 | 14 | 14 | 6.929 | 7.460 | 0.009 | 0.314 |
| zgc:56071 [Danio rerio] | CR364597 | 14 | 14 | 9.438 | 9.867 | 0.009 | 0.314 |

**Table S3.** *Cont.*

| **(e) Kidney: Brienzlig deformed (Pop1) *vs.* Brienzlig normal (Pop2)** |  |  |  |  |  |  |  |
| --- | --- | --- | --- | --- | --- | --- | --- |
| **Gene ID** | **Tigr ID** | **N_1_** | **N_2_** | **x_1_** | **x_2_** | ***P*** | **FDR** |
| PREDICTED: similar to transcription factor IIB [Gallus gallus] | CX034551 | 14 | 14 | 11.357 | 11.923 | 0.009 | 0.314 |
| Transposable element TC1 transposase | TC91574 | 14 | 14 | 6.448 | 6.816 | 0.009 | 0.314 |
| Microtubule-associated protein 1A (MAP 1A) (Fragment) | BX863284 | 14 | 14 | 5.568 | 6.008 | 0.009 | 0.314 |
| hypothetical protein CG003 [Homo sapiens] | BX313343 | 14 | 14 | 6.665 | 7.113 | 0.009 | 0.314 |
| chromosome 14 open reading frame 45 [Homo sapiens] | TC76712 | 14 | 14 | 7.521 | 8.025 | 0.009 | 0.314 |
| histidine triad nucleotide binding protein 3; HIT-like protein | CR374828 | 14 | 14 | 7.734 | 8.163 | 0.009 | 0.314 |
| Collagen alpha 1(IV) chain precursor | CX038308 | 14 | 14 | 9.206 | 9.853 | 0.009 | 0.314 |
| Zgc:73343 protein [Danio rerio] | CA385919 | 14 | 14 | 10.450 | 9.807 | 0.009 | 0.314 |
| transitin [Gallus gallus] | CA358514 | 14 | 14 | 9.373 | 9.721 | 0.009 | 0.314 |
| Hepatocyte nuclear factor 1-beta (HNF-1beta) (HNF-1B) | CA354659 | 14 | 14 | 11.668 | 12.317 | 0.009 | 0.314 |
| heparanase [Rattus norvegicus] | TC86032 | 14 | 14 | 6.432 | 6.215 | 0.009 | 0.314 |
| Homeobox protein Nkx-3.2 (Bagpipe homeobox protein homolog | BX866037 | 14 | 14 | 9.245 | 10.037 | 0.009 | 0.314 |
| Diablo homolog, mitochondrial precursor (Second | TC90159 | 14 | 14 | 8.521 | 8.014 | 0.009 | 0.314 |
| IFT172 [Danio rerio] | TC86063 | 14 | 14 | 9.136 | 9.781 | 0.009 | 0.314 |
| PREDICTED: similar to bA207C16.2 (novel protein similar to C. elegans | CA374581 | 14 | 14 | 8.624 | 9.144 | 0.009 | 0.314 |
| Collagen alpha 1(V) chain precursor | TC88418 | 14 | 14 | 10.129 | 10.585 | 0.009 | 0.314 |
| PREDICTED: similar to apoptosis-inducing factor (AIF)-like | BX857198 | 14 | 14 | 11.262 | 10.891 | 0.009 | 0.314 |
| MGC84244 protein [Xenopus laevis] | TC82309 | 14 | 14 | 8.578 | 9.095 | 0.009 | 0.314 |
| WD-repeat protein 13 | BX077103 | 14 | 14 | 7.166 | 7.474 | 0.009 | 0.314 |
| Regulator of G-protein signaling 1 (RGS1) | CA378631 | 14 | 14 | 6.103 | 5.715 | 0.009 | 0.314 |
| hypothetical protein MGC77803 [Danio rerio] | TC80299 | 14 | 14 | 8.243 | 8.760 | 0.009 | 0.314 |
| CC chemokine CK-2.1 [Oncorhynchus mykiss] | AY372431 | 14 | 14 | 11.920 | 12.506 | 0.009 | 0.314 |
| Testis-specific gene A2 (Male meiotic metaphase | TC76347 | 14 | 14 | 8.446 | 8.980 | 0.009 | 0.314 |
| similar to nudix (nucleotide diphosphate linked moiety X)-type | TC90354 | 14 | 14 | 8.711 | 8.188 | 0.009 | 0.314 |
| Transcription factor E3 | CA371133 | 14 | 14 | 11.064 | 11.635 | 0.009 | 0.314 |
| Serine/threonine phosphatase 4 regulatory subunit 1 | TC82396 | 14 | 14 | 10.890 | 11.470 | 0.009 | 0.314 |
| Integrin beta-1* precursor | CA382877 | 14 | 14 | 9.159 | 8.823 | 0.009 | 0.314 |
| Potassium voltage-gated channel subfamily A member 2 | TC93697 | 14 | 14 | 10.999 | 11.445 | 0.010 | 0.314 |
| Renal sodium-dependent phosphate transport protein 2 | TC90153 | 14 | 14 | 5.816 | 6.241 | 0.010 | 0.314 |
| SI:bZ1D10.1.1 (novel protein similar to human matrin 3 (MATR3)) | CA367384 | 14 | 14 | 6.516 | 6.998 | 0.010 | 0.314 |
| Transcriptional activator protein PUR-alpha (Purine-rich | TC89264 | 14 | 14 | 9.965 | 9.591 | 0.010 | 0.314 |
| HLA class II histocompatibility antigen, gamma chain | TC70179 | 14 | 14 | 14.092 | 14.871 | 0.010 | 0.314 |
| mitochondrial ribosomal protein L22 [Homo sapiens] | TC72563 | 14 | 14 | 6.659 | 7.198 | 0.010 | 0.314 |
| Platelet-derived growth factor, A chain precursor (PDGF | CA378413 | 14 | 14 | 6.772 | 7.507 | 0.010 | 0.314 |
| Tuberin (Tuberous sclerosis 2 homolog protein) | TC79928 | 14 | 14 | 12.968 | 13.554 | 0.010 | 0.314 |
| Heterogeneous nuclear ribonucleoprotein L (hnRNP L) | TC75457 | 14 | 14 | 9.813 | 10.390 | 0.010 | 0.314 |
| Neurogenic locus notch homolog protein 2 precursor (Notch 2) | BX877388 | 14 | 14 | 8.853 | 9.333 | 0.010 | 0.314 |
| Ubiquitin-like protein FUBI | TC86521 | 14 | 14 | 9.194 | 9.827 | 0.010 | 0.314 |
| PREDICTED: similar to hypothetical protein FLJ20397 [Gallus gallus] | BX867062 | 14 | 14 | 8.511 | 9.080 | 0.010 | 0.314 |
| solute carrier family 40 (iron-regulated transporter), member 1; | TC88597 | 14 | 14 | 10.290 | 10.863 | 0.010 | 0.314 |
| Pleckstrin homology domain-containing protein family A | CA352959 | 14 | 14 | 8.959 | 9.435 | 0.010 | 0.314 |
| Cysteine-rich protein 2 (CRP2) (ESP1 protein) | TC89097 | 14 | 14 | 9.288 | 9.700 | 0.010 | 0.314 |

**Table S3.** *Cont.*

| **(e) Kidney: Brienzlig deformed (Pop1) *vs.* Brienzlig normal (Pop2)** |  |  |  |  |  |  |  |
| --- | --- | --- | --- | --- | --- | --- | --- |
| **Gene ID** | **Tigr ID** | **N_1_** | **N_2_** | **x_1_** | **x_2_** | ***P*** | **FDR** |
| hepatocyte nuclear factor 4, alpha [Danio rerio] | TC92741 | 14 | 14 | 12.670 | 13.238 | 0.010 | 0.314 |
| Class I histocompatibility antigen, F10 alpha chain | TC86585 | 14 | 14 | 10.433 | 8.717 | 0.010 | 0.314 |
| Trypsin II precursor (EC 3.4.21.4) (Fragment) | BX077431 | 14 | 14 | 8.160 | 8.569 | 0.010 | 0.314 |
| Interleukin-1 receptor-associated kinase 1 (EC 2.7.1.-) | TC83959 | 14 | 14 | 8.393 | 8.938 | 0.010 | 0.314 |
| Hydroxymethylglutaryl-CoA synthase, cytoplasmic (EC | CA354171 | 14 | 14 | 10.101 | 10.688 | 0.010 | 0.314 |
| Parvalbumin alpha | TC70551 | 14 | 14 | 7.045 | 7.491 | 0.010 | 0.314 |
| Nuclear factor NF-kappa-B p105 subunit [Contains: Nuclear | TC78814 | 14 | 14 | 7.834 | 8.138 | 0.010 | 0.314 |
| Beta-1,3-galactosyl-O-glycosyl-glycoprotein | CA366541 | 14 | 14 | 6.282 | 6.644 | 0.010 | 0.314 |
| mKIAA1606 protein [Mus musculus] | TC83742 | 14 | 14 | 8.552 | 8.319 | 0.010 | 0.314 |
| Alpha-1D adrenergic receptor (Alpha 1D-adrenoceptor) | TC76508 | 14 | 14 | 7.713 | 8.163 | 0.010 | 0.314 |
| PREDICTED: similar to cobl-related 1 [Gallus gallus] | TC80260 | 14 | 14 | 10.444 | 11.110 | 0.010 | 0.314 |
| Glycine amidinotransferase, mitochondrial precursor (EC | TC87087 | 14 | 14 | 9.915 | 10.403 | 0.010 | 0.314 |
| Fibrinogen alpha chain [Contains: Fibrinopeptide A] | BX879025 | 14 | 14 | 5.689 | 6.150 | 0.010 | 0.314 |
| Tetratricopeptide repeat protein 9 (TPR repeat protein 9) | TC80615 | 14 | 14 | 12.126 | 12.807 | 0.010 | 0.314 |
| unnamed protein product [Tetraodon nigroviridis] | TC87465 | 14 | 14 | 8.083 | 7.509 | 0.010 | 0.314 |
| Similar to hypothetical protein, estradiol-induced; wu:fd51d02 | CX040292 | 14 | 14 | 10.894 | 11.510 | 0.010 | 0.314 |
| unnamed protein product [Tetraodon nigroviridis] | CX026684 | 14 | 14 | 9.803 | 10.315 | 0.010 | 0.314 |
| Splicing factor, arginine/serine-rich 1 (pre-mRNA splicing | TC79288 | 14 | 14 | 9.059 | 9.437 | 0.010 | 0.314 |
| endothelial cell adhesion molecule [Rattus norvegicus] | CX034363 | 14 | 14 | 12.313 | 12.837 | 0.010 | 0.314 |
| growth hormone receptor isoform 2 [Oncorhynchus kisutch] | AY663793 | 14 | 14 | 9.562 | 10.161 | 0.010 | 0.314 |
|  |  |  |  |  |  |  |  |
| **(f) Kidney: all deformed (pooled Albock and Brienzlig) *vs.* all normal** |  |  |  |  |  |  |  |
| **Gene ID** | **Tigr ID** | **N_1_** | **N_2_** | **x_1_** | **x_2_** | ***P*** | **FDR** |
| Ictacalcin | BX914141 | 28 | 28 | 12.520 | 11.715 | 0.000 | 0.952 |
| Myosin light polypeptide 6 (Myosin light chain alkali 3) | TC70741 | 28 | 28 | 11.252 | 10.717 | 0.000 | 0.952 |
| Ictacalcin | CX026130 | 28 | 28 | 12.419 | 11.683 | 0.001 | 0.952 |
| unnamed protein product [Tetraodon nigroviridis] | CA372555 | 28 | 28 | 5.728 | 5.933 | 0.001 | 0.952 |
| TBC1 domain family protein C20orf140 | BX878540 | 28 | 28 | 8.143 | 7.999 | 0.001 | 0.952 |
| Similar to hypothetical protein FLJ10856 [Danio rerio] | TC88607 | 28 | 28 | 8.599 | 8.052 | 0.001 | 0.952 |
| unnamed protein product [Tetraodon nigroviridis] | TC82829 | 28 | 28 | 6.419 | 5.472 | 0.001 | 0.952 |
| Forkhead box protein J3 | TC84177 | 28 | 28 | 12.816 | 13.086 | 0.001 | 0.952 |
| NAD(P)H dehydrogenase [quinone] 1 (EC 1.6.99.2) (Quinone | TC70300 | 28 | 28 | 10.553 | 10.864 | 0.001 | 0.952 |
| Cytochrome b | TC69298 | 28 | 28 | 14.198 | 14.433 | 0.001 | 0.952 |
| UDP-glucuronosyltransferase 1-5 precursor, microsomal (EC | CA381172 | 28 | 28 | 7.787 | 7.987 | 0.001 | 0.952 |
| Polyhomeotic-like protein 1 (Early development regulator | TC87270 | 28 | 28 | 10.645 | 10.911 | 0.001 | 0.952 |
| Brush border 61.9 kDa protein precursor | CA354102 | 28 | 28 | 9.052 | 9.496 | 0.001 | 0.952 |
| Nedd-4-like ubiquitin-protein ligase WWP2 (EC 6.3.2.-) (WW | TC93189 | 28 | 28 | 5.459 | 5.601 | 0.001 | 0.952 |
| unnamed protein product [Tetraodon nigroviridis] | BX082162 | 28 | 28 | 11.902 | 12.293 | 0.002 | 0.952 |
| Frizzled-related protein precursor (Frzb-1) (Frezzled) | TC93175 | 28 | 28 | 5.754 | 5.450 | 0.002 | 0.952 |
| unnamed protein product [Tetraodon nigroviridis] | BX315942 | 28 | 28 | 8.863 | 8.610 | 0.002 | 0.952 |
| Dedicator of cytokinesis protein 10 (Protein zizimin 3) | CF753039 | 28 | 28 | 6.180 | 6.303 | 0.002 | 0.952 |
| TPA: transposase [Rana pipiens] | CA367179 | 28 | 28 | 13.716 | 13.878 | 0.002 | 0.952 |
| PREDICTED: similar to angiotensin II receptor-associated protein; | TC91925 | 28 | 28 | 9.658 | 9.196 | 0.002 | 0.952 |

**Table S3.** *Cont.*

| **(f) Kidney: all deformed (pooled Albock and Brienzlig) *vs.* all normal** |  |  |  |  |  |  |  |
| --- | --- | --- | --- | --- | --- | --- | --- |
| **Gene ID** | **Tigr ID** | **N_1_** | **N_2_** | **x_1_** | **x_2_** | ***P*** | **FDR** |
| Brush border 61.9 kDa protein precursor | CA378216 | 28 | 28 | 8.281 | 8.914 | 0.003 | 0.952 |
| PREDICTED: hypothetical protein XP_420093 [Gallus gallus] | BX878213 | 28 | 28 | 14.692 | 14.851 | 0.003 | 0.952 |
| Mitochondrial 28S ribosomal protein S36 (S36mt) (MRP-S36) | TC87060 | 28 | 28 | 10.043 | 9.831 | 0.003 | 0.952 |
| Integrin beta pat-3 precursor | CA369883 | 28 | 28 | 7.989 | 7.302 | 0.003 | 0.952 |
| Anthrax toxin receptor 1 precursor (Tumor endothelial | BX866999 | 28 | 28 | 8.345 | 8.661 | 0.003 | 0.952 |
| Transposable element TCB1 transposase (Transposable | CA384381 | 28 | 28 | 15.160 | 15.289 | 0.003 | 0.952 |
| BTB/POZ domain containing protein 9 | CR373080 | 28 | 28 | 14.131 | 14.399 | 0.003 | 0.952 |
| Brush border 61.9 kDa protein precursor | BX088051 | 28 | 28 | 8.153 | 9.017 | 0.004 | 0.952 |
| PREDICTED: similar to WW domain-containing binding protein 4; | CA357832 | 28 | 28 | 6.449 | 5.972 | 0.004 | 0.952 |
| unnamed protein product [Tetraodon nigroviridis] | TC87269 | 28 | 28 | 6.872 | 6.592 | 0.004 | 0.952 |
| Protein phosphatase 2C alpha isoform (EC 3.1.3.16) | TC84962 | 28 | 28 | 8.445 | 8.269 | 0.004 | 0.952 |
| unnamed protein product [Tetraodon nigroviridis] | TC80726 | 28 | 28 | 11.258 | 11.099 | 0.004 | 0.952 |
| unnamed protein product [Tetraodon nigroviridis] | BX086590 | 28 | 28 | 10.040 | 9.871 | 0.004 | 0.952 |
| GPI-anchor transamidase precursor (EC 3.-.-.-) (GPI | TC81404 | 28 | 28 | 8.895 | 8.461 | 0.004 | 0.952 |
| caspase 8 associated protein 2 [Mus musculus] | BX076447 | 28 | 28 | 7.316 | 7.649 | 0.004 | 0.952 |
| Type I inositol-1,4,5-trisphosphate 5-phosphatase (EC | CA351545 | 28 | 28 | 5.587 | 5.402 | 0.004 | 0.952 |
| Dapper 1 (XDpr) | TC89983 | 28 | 28 | 5.989 | 6.282 | 0.004 | 0.952 |
| TPA: transposase [Rana pipiens] | TC81801 | 28 | 28 | 15.428 | 15.553 | 0.005 | 0.952 |
| Claudin-6 (Skullin 2) (UNQ757/PRO1488) | TC88355 | 28 | 28 | 5.687 | 6.101 | 0.005 | 0.952 |
| unnamed protein product [Tetraodon nigroviridis] | TC72840 | 28 | 28 | 6.413 | 6.113 | 0.005 | 0.952 |
| Protein STRAIT11499 homolog | TC73512 | 28 | 28 | 11.176 | 10.460 | 0.005 | 0.952 |
| Keratin associated protein 4-5 (Keratin associated protein | BX885050 | 28 | 28 | 15.533 | 15.661 | 0.005 | 0.952 |
| CREB-binding protein (EC 2.3.1.48) | TC89694 | 28 | 28 | 6.293 | 6.678 | 0.005 | 0.952 |
| unnamed protein product [Tetraodon nigroviridis] | TC84301 | 28 | 28 | 5.831 | 7.164 | 0.005 | 0.952 |
| HLA class II histocompatibility antigen, gamma chain | TC70179 | 28 | 28 | 14.512 | 15.151 | 0.005 | 0.952 |
| Mid-1-related chloride channel 1 [Mus musculus] | CA355526 | 28 | 28 | 8.551 | 8.264 | 0.005 | 0.952 |
| PREDICTED: similar to RIKEN cDNA 1110001J12 [Gallus gallus] | TC93033 | 28 | 28 | 5.757 | 5.866 | 0.005 | 0.952 |
| Pyridoxine-5'-phosphate oxidase (EC 1.4.3.5) | TC81189 | 28 | 28 | 8.923 | 8.532 | 0.005 | 0.952 |
| Maintenance of ploidy protein mob1 | TC91626 | 28 | 28 | 10.520 | 10.258 | 0.005 | 0.952 |
| PREDICTED: similar to Chain L, Crystal Structure Of The Fab | TC78002 | 28 | 28 | 11.340 | 9.869 | 0.005 | 0.952 |
| Serine protease HTRA1 precursor (EC 3.4.21.-) (L56) | TC71882 | 28 | 28 | 7.280 | 6.609 | 0.005 | 0.952 |
| Filamin-binding LIM protein-1 (FBLP-1) (Mitogen-inducible 2 | TC80130 | 28 | 28 | 5.579 | 5.411 | 0.005 | 0.952 |
| Baculoviral IAP repeat-containing protein 6 | CB491639 | 28 | 28 | 16.194 | 16.357 | 0.006 | 0.952 |
| 4-methyl-5(B-hydroxyethyl)-thiazole monophosphate | TC78648 | 28 | 28 | 9.556 | 9.315 | 0.006 | 0.952 |
| Coilin (p80) | TC80733 | 28 | 28 | 5.528 | 5.699 | 0.006 | 0.952 |
| Transposable element TCB1 transposase (Transposable | CA383269 | 28 | 28 | 11.806 | 11.944 | 0.006 | 0.952 |
| GPI-anchored protein p137 (p137GPI) | CA386677 | 28 | 28 | 6.711 | 6.876 | 0.006 | 0.952 |
| Tetratricopeptide repeat protein 9 (TPR repeat protein 9) | TC71416 | 28 | 28 | 10.917 | 10.794 | 0.006 | 0.952 |
| Von Hippel-Lindau disease tumor suppressor (pVHL) | TC85757 | 28 | 28 | 6.946 | 6.763 | 0.006 | 0.952 |
| Transposable element TCB2 transposase | BX081012 | 28 | 28 | 11.142 | 11.261 | 0.006 | 0.952 |
| unnamed protein product [Tetraodon nigroviridis] | TC78605 | 28 | 28 | 6.725 | 7.024 | 0.006 | 0.952 |
| Aquaporin 8 | TC92050 | 28 | 28 | 13.997 | 13.538 | 0.006 | 0.952 |
| Spectrin beta chain, brain 1 (Spectrin, non-erythroid beta | TC89216 | 28 | 28 | 6.032 | 6.211 | 0.006 | 0.952 |
| Serine hydrolase-like protein (EC 3.1.-.-) | BX320980 | 28 | 28 | 8.044 | 8.205 | 0.006 | 0.952 |

**Table S3.** *Cont.*

| **(f) Kidney: all deformed (pooled Albock and Brienzlig) *vs.* all normal** |  |  |  |  |  |  |  |
| --- | --- | --- | --- | --- | --- | --- | --- |
| **Gene ID** | **Tigr ID** | **N_1_** | **N_2_** | **x_1_** | **x_2_** | ***P*** | **FDR** |
| NifU-like protein | TC78409 | 28 | 28 | 14.441 | 14.114 | 0.007 | 0.952 |
| 3-oxoacyl-[acyl-carrier-protein] synthase II (EC 2.3.1.41) | BX084635 | 28 | 28 | 5.440 | 5.192 | 0.007 | 0.952 |
| unnamed protein product [Tetraodon nigroviridis] | TC89193 | 28 | 28 | 10.771 | 10.618 | 0.007 | 0.952 |
| Rho guanine nucleotide exchange factor 5 (Guanine | BX870290 | 28 | 28 | 5.938 | 5.694 | 0.007 | 0.952 |
| unnamed protein product [Tetraodon nigroviridis] | TC93109 | 28 | 28 | 6.511 | 6.718 | 0.007 | 0.952 |
| unnamed protein product [Tetraodon nigroviridis] | TC86217 | 28 | 28 | 8.585 | 8.164 | 0.007 | 0.952 |
| unnamed protein product [Tetraodon nigroviridis] | TC70359 | 28 | 28 | 12.904 | 12.538 | 0.007 | 0.952 |
| PREDICTED: similar to meningioma expressed antigen 5 (hyaluronidase) | BX912183 | 28 | 28 | 6.401 | 6.617 | 0.007 | 0.952 |
| Brush border 61.9 kDa protein precursor | BX088050 | 28 | 28 | 9.577 | 10.493 | 0.008 | 0.952 |
| Transposable element TCB1 transposase (Transposable | TC73311 | 28 | 28 | 14.811 | 14.965 | 0.008 | 0.952 |
| Vesicle-associated membrane protein 3 (VAMP-3) | BX073534 | 28 | 28 | 10.807 | 10.452 | 0.008 | 0.952 |
| Pyridoxine-5'-phosphate oxidase (EC 1.4.3.5) | BX866992 | 28 | 28 | 8.553 | 8.274 | 0.008 | 0.952 |
| DNA (cytosine-5)-methyltransferase 1 (EC 2.1.1.37) (Dnmt1) | CX034411 | 28 | 28 | 6.593 | 6.754 | 0.008 | 0.952 |
| wu:fb11h03; lipocalin-type prostaglandin D synthase-like; | CA358499 | 28 | 28 | 7.901 | 7.329 | 0.008 | 0.952 |
| NADH-ubiquinone oxidoreductase 18 kDa subunit, | TC70031 | 28 | 28 | 13.389 | 13.252 | 0.008 | 0.952 |
| Fibrinogen alpha chain [Contains: Fibrinopeptide A] | BX879025 | 28 | 28 | 5.736 | 6.064 | 0.008 | 0.952 |
| Prp-8-prov protein [Xenopus laevis] | TC77402 | 28 | 28 | 9.371 | 9.527 | 0.008 | 0.952 |
| invariant chain INVX [Oncorhynchus mykiss] | AY065837 | 28 | 28 | 14.261 | 14.892 | 0.008 | 0.952 |
| Transposable element TCB2 transposase | CA353291 | 28 | 28 | 13.419 | 13.596 | 0.008 | 0.952 |
| unnamed protein product [Tetraodon nigroviridis] | TC74075 | 28 | 28 | 9.725 | 9.410 | 0.008 | 0.952 |
| hypothetical protein [Homo sapiens] | TC75092 | 28 | 28 | 14.749 | 15.270 | 0.008 | 0.952 |
| Forkhead box protein P4 (Fork head-related protein like A) | BX867531 | 28 | 28 | 6.728 | 6.860 | 0.008 | 0.952 |
| RNA helicase-like protein DB10 | TC81064 | 28 | 28 | 10.488 | 10.023 | 0.008 | 0.952 |
| NAD-dependent deacetylase sirtuin 6 (EC 3.5.1.-) | CX038407 | 28 | 28 | 6.773 | 6.922 | 0.008 | 0.952 |
| tumour necrosis factor receptor associated factor 3 [Oncorhynchus | AJ517803 | 28 | 28 | 5.798 | 5.640 | 0.009 | 0.952 |
| KIAA1040 protein [Homo sapiens] | CA371127 | 28 | 28 | 9.553 | 9.688 | 0.009 | 0.952 |
| RIKEN cDNA 1810011O10 [Mus musculus] | CA358909 | 28 | 28 | 8.557 | 8.142 | 0.009 | 0.952 |
| ADAM 22 precursor (A disintegrin and metalloproteinase | TC94058 | 28 | 28 | 8.487 | 8.689 | 0.009 | 0.952 |
| Pyridoxine-5'-phosphate oxidase (EC 1.4.3.5) | TC75464 | 28 | 28 | 8.128 | 7.781 | 0.009 | 0.952 |
| Microtubule-associated protein 1A (MAP 1A) (Fragment) | BX863284 | 28 | 28 | 5.627 | 5.907 | 0.009 | 0.952 |
| Diacylglycerol kinase, delta (EC 2.7.1.107) (Diglyceride | CA351948 | 28 | 28 | 5.364 | 5.512 | 0.009 | 0.952 |
| similar to RIKEN cDNA C230094B15 [Rattus norvegicus] | CA345414 | 28 | 28 | 7.827 | 8.122 | 0.010 | 0.952 |
| Pellino protein homolog 3 (Pellino 3) | CA355560 | 28 | 28 | 8.753 | 8.063 | 0.010 | 0.952 |
| PREDICTED: similar to angiotensin II receptor-associated protein; | TC82599 | 28 | 28 | 8.099 | 7.698 | 0.010 | 0.952 |
| Transposable element TCB1 transposase (Transposable | TC69526 | 28 | 28 | 16.666 | 16.813 | 0.010 | 0.952 |
| Tetratricopeptide repeat protein 9 (TPR repeat protein 9) | TC71414 | 28 | 28 | 9.360 | 9.237 | 0.010 | 0.952 |
| Microfibril-associated glycoprotein 4 precursor | TC88635 | 28 | 28 | 7.029 | 6.232 | 0.010 | 0.952 |
| Delta-like protein 4 precursor (Drosophila Delta homolog | CA382148 | 28 | 28 | 6.060 | 6.209 | 0.010 | 0.952 |

**Table S4.** Gene Ontology (GO) categories that comprised genes that showed significant enrichment of low *P*-value ranking among all genes at the 1% level for liver (a,b) and head kidney (c,d) between whitefish gonadal phenotypes (e.g., Albock normal (nor) *vs.* Albock deformed (def)). *P*-values and corresponding FDR were determined by the program FUNC (Prufer *et al*. 2007). Significant GO categories after refinement analysis are indicated with asterisks and for GO term with number of genes < 200 shown in bold (according to Table 2).

| **(a)** | **Liver: Albock nor *vs.* def** | |  |  |  |  |
| --- | --- | --- | --- | --- | --- | --- |
|  | **GO root node** | **Name** | **GO ID** | **Genes** | ***P*** | **FDR** |
|  | biological_process | **immune response*** | GO:0006955 | 70 | 0.000 | 0.002 |
|  | biological_process | **humoral immune response*** | GO:0006959 | 22 | 0.001 | 0.042 |
|  | biological_process | **innate immune response*** | GO:0045087 | 25 | 0.001 | 0.071 |
|  | biological_process | immune system process | GO:0002376 | 107 | 0.001 | 0.071 |
|  | biological_process | **activation of immune response*** | GO:0002253 | 22 | 0.002 | 0.099 |
|  | biological_process | positive regulation of response to stimulus | GO:0048584 | 24 | 0.003 | 0.099 |
|  | biological_process | **proteolysis*** | GO:0006508 | 160 | 0.004 | 0.116 |
|  | biological_process | positive regulation of immune response | GO:0050778 | 23 | 0.004 | 0.116 |
|  | biological_process | positive regulation of immune system process | GO:0002684 | 25 | 0.004 | 0.116 |
|  | biological_process | regulation of immune system process | GO:0002682 | 30 | 0.007 | 0.346 |
|  | cellular_component | **integral to plasma membrane*** | GO:0005887 | 60 | 0.009 | 0.346 |
|  | cellular_component | intrinsic to plasma membrane | GO:0031226 | 60 | 0.009 | 0.346 |
|  | biological_process | **immune effector process*** | GO:0002252 | 32 | 0.009 | 0.482 |
| **(b)** | **Liver: Brienzlig nor *vs.* def** | |  |  |  |  |
|  | **GO root node** | **Name** | **GO ID** | **Genes** | ***P*** | **FDR** |
|  | cellular_component | **extracellular region*** | GO:0005576 | 197 | 0.002 | 0.041 |
|  | cellular_component | **proteinaceous extracellular matrix*** | GO:0005578 | 31 | 0.002 | 0.041 |
|  | cellular_component | extracellular matrix | GO:0031012 | 31 | 0.002 | 0.041 |
|  | cellular_component | extracellular region part | GO:0044421 | 78 | 0.005 | 0.067 |
|  | biological_process | **immune system process*** | GO:0002376 | 107 | 0.006 | 0.602 |
|  | biological_process | **cell adhesion*** | GO:0007155 | 85 | 0.009 | 0.726 |
|  | biological_process | biological adhesion | GO:0022610 | 85 | 0.009 | 0.726 |
| **(c)** | **Head kidney: Albock nor *vs.* def** | |  |  |  |  |
|  | **GO root node** | **Name** | **GO ID** | **Genes** | ***P*** | **FDR** |
|  | biological_process | **regulation of cell proliferation*** | GO:0042127 | 114 | 0.000 | 0.014 |
|  | biological_process | cell proliferation | GO:0008283 | 155 | 0.000 | 0.014 |
|  | biological_process | **positive regulation of cell proliferation*** | GO:0008284 | 54 | 0.000 | 0.014 |
|  | biological_process | **embryonic development*** | GO:0009790 | 86 | 0.000 | 0.017 |
|  | biological_process | **protein import into nucleus*** | GO:0006606 | 23 | 0.001 | 0.050 |
|  | biological_process | cell motion | GO:0006928 | 72 | 0.001 | 0.050 |
|  | biological_process | localization of cell | GO:0051674 | 72 | 0.001 | 0.050 |
|  | molecular_function | **ATP-dependent helicase activity*** | GO:0008026 | 20 | 0.001 | 0.050 |
|  | molecular_function | purine NTP-dependent helicase activity | GO:0070035 | 20 | 0.001 | 0.050 |
|  | biological_process | **cell migration*** | GO:0016477 | 52 | 0.001 | 0.050 |
|  | biological_process | cell motility | GO:0048870 | 52 | 0.001 | 0.050 |
|  | biological_process | nuclear import | GO:0051170 | 24 | 0.002 | 0.061 |
|  | biological_process | **embryonic development ending in birth or  egg hatching*** | GO:0009792 | 58 | 0.002 | 0.068 |
|  | biological_process | organ development* | GO:0048513 | 254 | 0.003 | 0.103 |

**Table S4.** *Cont.*

| **(c)** | **Head kidney: Albock nor *vs.* def** | |  |  |  |  |
| --- | --- | --- | --- | --- | --- | --- |
|  | **GO root node** | **Name** | **GO ID** | **Genes** | ***P*** | **FDR** |
|  | molecular_function | **small GTPase regulator activity*** | GO:0005083 | 49 | 0.004 | 0.123 |
|  | biological_process | **regulation of Ras protein signal transduction*** | GO:0046578 | 27 | 0.004 | 0.123 |
|  | biological_process | locomotion | GO:0040011 | 72 | 0.006 | 0.170 |
|  | biological_process | protein import | GO:0017038 | 31 | 0.007 | 0.180 |
|  | biological_process | nucleocytoplasmic transport | GO:0006913 | 32 | 0.008 | 0.186 |
|  | biological_process | nuclear transport | GO:0051169 | 32 | 0.008 | 0.186 |
|  | biological_process | Ras protein signal transduction | GO:0007265 | 45 | 0.009 | 0.186 |
|  | biological_process | **cellular protein complex assembly*** | GO:0043623 | 41 | 0.009 | 0.186 |
|  | biological_process | **regulation of immune system process*** | GO:0002682 | 45 | 0.009 | 0.186 |
|  | biological_process | **central nervous system development*** | GO:0007417 | 45 | 0.010 | 0.186 |
| **(d)** | **Head kidney: Brienzlig nor *vs.* def** | |  |  |  |  |
|  | **GO root node** | **Name** | **GO ID** | **Genes** | ***P*** | **FDR** |
|  | biological_process | regulation of biological process | GO:0050789 | 1109 | 0.000 | 0.028 |
|  | biological_process | regulation of cellular process | GO:0050794 | 1058 | 0.000 | 0.028 |
|  | biological_process | cell communication | GO:0007154 | 560 | 0.000 | 0.028 |
|  | biological_process | signal transduction* | GO:0007165 | 506 | 0.001 | 0.047 |
|  | biological_process | biological regulation | GO:0065007 | 1191 | 0.001 | 0.047 |
|  | molecular_function | **GTPase activator activity*** | GO:0005096 | 36 | 0.003 | 0.301 |
|  | molecular_function | enzyme activator activity | GO:0008047 | 58 | 0.004 | 0.391 |
|  | biological_process | hemopoiesis | GO:0030097 | 51 | 0.005 | 0.467 |
|  | biological_process | **leukocyte differentiation*** | GO:0002521 | 32 | 0.006 | 0.467 |
|  | biological_process | **gamete generation*** | GO:0007276 | 40 | 0.006 | 0.467 |
|  | biological_process | regulation of metabolic process | GO:0019222 | 510 | 0.008 | 0.467 |
|  | biological_process | **T cell activation*** | GO:0042110 | 31 | 0.008 | 0.467 |
|  | cellular_component | **soluble fraction*** | GO:0005625 | 32 | 0.009 | 0.467 |
|  | biological_process | muscle system process | GO:0003012 | 29 | 0.010 | 0.571 |
|  | biological_process | **muscle contraction*** | GO:0006936 | 28 | 0.010 | 0.571 |
|  | biological_process | regulation of macromolecule metabolic process* | GO:0060255 | 485 | 0.010 | 0.571 |

**Figure S1.** Evaluation of preliminary array experiment for use of whitefish samples on an *in situ*-synthesized oligonucleotide array originally designed for rainbow trout. Log2 transformed raw signal intensity frequency distribution of 21’492 features originating from rainbow trout EST libraries. (**a**) rainbow trout liver; (**b**) whitefish liver; (**c**) rainbow trout head kidney; and (**d**) whitefish head kidney.
